# Associations of Albuminuria and Metabolic Syndrome Traits With Fracture Risk in Patients With Type 2 Diabetes: A Population‐Based Cohort Study

**DOI:** 10.1002/jcsm.70215

**Published:** 2026-02-02

**Authors:** Xi Xiong, David T. W. Lui, Chengsheng Ju, Xiaodong Liu, Li Wei, Manju Chandran, Carlos K. H. Wong

**Affiliations:** ^1^ Centre for Safe Medication Practice and Research, Department of Pharmacology and Pharmacy, Li Ka Shing Faculty of Medicine The University of Hong Kong Hong Kong SAR China; ^2^ Laboratory of Data Discovery for Health (D^2^4H) Hong Kong Science and Technology Park Hong Kong SAR China; ^3^ Research Department of Practice and Policy, School of Pharmacy University College London London UK; ^4^ Department of Non‐Communicable Disease Epidemiology, Faculty of Epidemiology and Population Health London School of Hygiene and Tropical Medicine London UK; ^5^ Department of Medicine, School of Clinical Medicine, Li Ka Shing Faculty of Medicine The University of Hong Kong Hong Kong SAR China; ^6^ Asia Pacific Consortium on Osteoporosis (APCO) Hong Kong SAR China; ^7^ Department of Surgery, School of Clinical Medicine, Li Ka Shing Faculty of Medicine The University of Hong Kong Hong Kong SAR China; ^8^ Centre for Medicines Optimization Research and Education University College London Hospitals NHS Foundation Trust London UK; ^9^ Osteoporosis and Bone Metabolism Unit, Department of Endocrinology Singapore General Hospital Singapore Singapore; ^10^ DUKE NUS Medical School Singapore Singapore; ^11^ School of Public Health, Li Ka Shing Faculty of Medicine The University of Hong Kong Hong Kong SAR China; ^12^ Department of Infectious Disease Epidemiology & Dynamics, Faculty of Epidemiology and Population Health London School of Hygiene and Tropical Medicine London UK

**Keywords:** albuminuria, fracture, hip fracture, metabolic syndrome, obesity, type 2 diabetes

## Abstract

**Background:**

Type 2 diabetes is associated with an increased risk of fragility fractures. While obesity may protect against fractures, individuals with type 2 diabetes often exhibit other metabolic syndrome (MetS) traits and albuminuria. We evaluated their roles and synergistic implications on incident fractures, stratified by obesity status.

**Methods:**

Patients with type 2 diabetes were identified from territory‐wide electronic health records in Hong Kong (2000–2018). MetS‐related traits included albuminuria and individual MetS traits (obesity, hypertension, low HDL‐cholesterol and hypertriglyceridemia). Outcomes were hip and major osteoporotic fractures (MOF). Patients were followed until fracture, death or 31 December 2020. Adjusted hazard ratios (aHRs) were estimated using multivariable Cox models.

**Results:**

Among 165 289 patients with type 2 diabetes (median age: 60.0 years; 54.2% men), 1583 (0.96%) experienced hip fractures, and 3393 (2.05%) had MOF over a median follow‐up of 5.3 years. Albuminuria was the strongest risk factor for hip fractures (obese: aHR 1.33, 95% CI 1.11–1.60; non‐obese: 1.54, 1.33–1.78) and MOF (obese: 1.13, 1.01–1.26; non‐obese: 1.28, 1.15–1.43). Hypertension was a significant risk factor only in non‐obese patients. In the non‐obese group, each additional MetS‐related trait was associated with an increased risk of hip fracture and MOF. When stratified by diabetes duration, albuminuria remained a significant risk factor across different diabetes durations, while suboptimal glycaemic control became a significant risk factor particularly when diabetes duration ≥ 5 years.

**Conclusions:**

In this large population‐based cohort of patients with type 2 diabetes predominantly of Asian descent from Hong Kong, albuminuria emerged as an important predictor of fracture risk. MetS traits compound this risk, especially in non‐obese individuals. These findings could be instrumental in shaping screening initiatives for fracture risk optimization in type 2 diabetes.

## 1 Introduction

Type 2 diabetes (T2D) is a significant global health problem, with a prevalence of 10.5% [1]. It is associated with various microvascular and macrovascular complications, especially in poor glycaemic control [2]. With an ageing population, osteoporosis and its complications—fragility fractures—emerge as another global health problem [3]. Fragility fractures are increasingly recognized as the complications of T2D with multiple studies showing the increased risk of fragility fractures in T2D [4]. It is suggested that the accumulation of advanced glycation end‐products leads to impaired bone material properties, while the microvascular disease leads to increased cortical porosity [5]. Thus, glycaemia‐related factors (including hyperglycaemia and hypoglycaemia) are key contributors to bone fragility in T2D [5]. The increased fall risk in patients with T2D, contributed in part by the presence of diabetic complications, adds to the fracture risks [5].

Among the major osteoporotic fractures, hip fractures are the most devastating, bringing about significant morbidities and mortality. About half of the patients who have sustained hip fracture may not regain the pre‐fracture level of mobility, and the one‐year mortality rate could be up to 20% [6]. The presence of diabetes is associated with 1.4–1.7 times the risk of hip fractures [7]. It compounds the post‐fracture complication rates and mortality [8], which is the ‘doubled burden’ of diabetic bone disease [9].

Patients with T2D often have co‐existing albuminuria and additional components of metabolic syndrome (MetS), encompassing a cluster of interrelated cardiometabolic abnormalities, including central obesity, hypertension and dyslipidaemia. A recent systematic review and meta‐analysis summarizes 20 studies on the association between MetS and fracture risk, yielding inconsistent results likely because most studies considered MetS as a dichotomous entity [10]. Among these components, obesity (primarily when defined by body mass index, BMI) has been consistently shown to be protective against hip fractures [11]. Our study aims to fill the knowledge gap by dissecting the various components of MetS and albuminuria in relation to fracture risk in patients with T2D, who are already at an increased risk of fragility fractures. Furthermore, it remains to be elucidated, among people with T2D, whether the increase in the number of MetS traits, together with albuminuria, would be associated with even higher fracture risks, and which specific trait or albuminuria holds the strongest association with the fracture risks. Such results would highlight the importance of addressing albuminuria and MetS traits for the optimal management of bone fragility in diabetes patients and prioritizing albuminuria and MetS components for management.

Hence, we carried out a retrospective cohort study to examine the association between albuminuria and MetS traits with hip fracture risk, using the territory‐wide cohort of individuals with T2D in Hong Kong.

## 2 Methods

### 2.1 Data Source

Electronic medical records were obtained from the Hong Kong Hospital Authority (HA) database, which manages all public hospitals and ambulatory clinics in Hong Kong. This system serves over 7.4 million residents, covering approximately 80% of all routine hospital admissions. The centralized records include demographic information, death dates, drug prescriptions, diagnoses, procedures and laboratory tests. The HA database has been widely used in studies monitoring long‐term outcomes in patients with T2D [12, 13]. Patients with T2D treated in HA public clinics undergo regular screenings for diabetic complications. These screenings include comprehensive clinical evaluations and laboratory tests to record diabetes duration, monitor diabetes control, assess cardiovascular risk factors and identify complications such as diabetic kidney disease through systematic measurements of albuminuria and estimated glomerular filtration rate (eGFR) according to standard of care [14].

The study utilized an anonymized HA database, and no patient‐identifying information was used. Ethical approval was granted by the Institutional Review Board of the Hong Kong Hospital Authority (Ref No. UW 21–320).

### 2.2 Study Population

**FIGURE 1:**
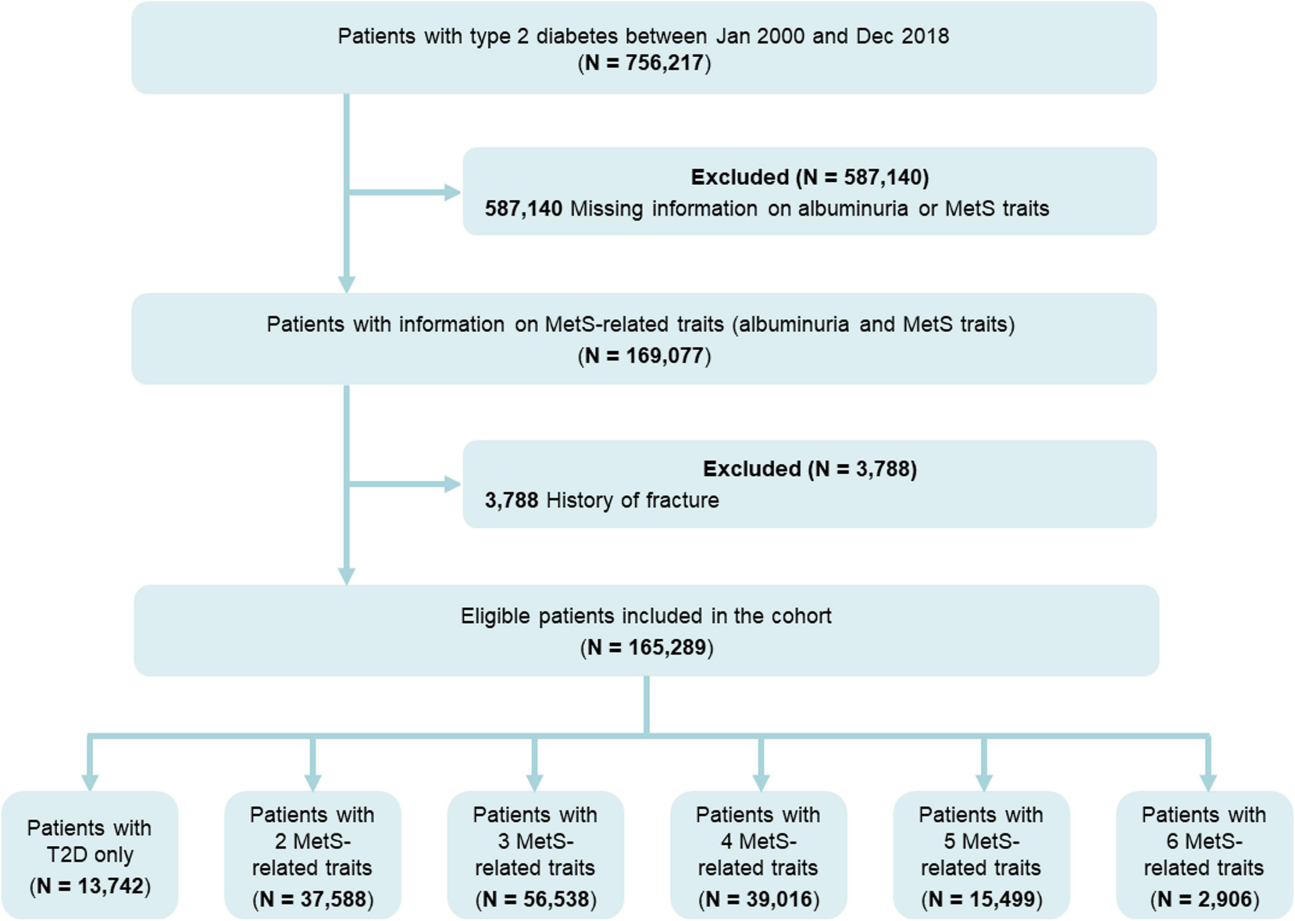
Study population flow diagram.

T2D patients aged 18 years or above were identified from the HA database between 1 January 2000 and 31 December 2018 in Hong Kong SAR, China. Diabetes was defined using International Classification of Diseases, Ninth Revision, Clinical Modification (ICD‐9‐CM) diagnosis codes (250), International Classification of Primary Care, 2nd edition (ICPC‐2) codes (T89 or T90), a glycated haemoglobin (HbA1c) level of ≥ 6.5% (48 mmol/mol) or a fasting glucose level of ≥ 7.0 mmol/L. Patients with a diabetes diagnosis but no record of type 1 diabetes (identified by ICD‐9‐CM codes [250.×1 or 250.×3] and ICPC‐2 codes [T89]) were classified as having T2D. To ensure comparability of albuminuria status and MetS traits, a 1‐year screening period after the T2D diagnosis was added for each patient to collect data on all relevant traits. The index date was defined as 1 year after T2D diagnosis. Patients were excluded if they (i) had missing information on one or more MetS traits or albuminuria or (ii) had a history of major osteoporotic fractures (clinical spine, hip, humerus and wrist—identified by validated ICD‐9‐CM codes [805, 812, 813, 814, 820]) [15] (Figure 1). Eligible patients were followed from the index date until death, the occurrence of outcomes or 31 December, 2020, whichever came first.

### 2.3 Definition of MetS Components

After reviewing widely used contemporary definitions of MetS, and given that all patients had T2D, we defined the MetS traits as hypertension, obesity, hypertriglyceridemia and low high‐density lipoprotein (HDL)‐cholesterol levels [16]. In this study, we used ‘MetS‐related traits’ to describe individual MetS traits together with albuminuria. Albuminuria was included in accordance with the World Health Organization (WHO) 1998 criteria [17, 18], although it is not part of most contemporary MetS definitions. The WHO 1998 criteria are considered more appropriate for populations with T2D than other available definitions, as they explicitly require the presence of T2D as a core component central to the pathophysiology of MetS. Hypertension was identified by a recorded diagnosis (ICD‐9‐CM 401‐405 or 437.2), a systolic blood pressure of 140 mmHg or higher, a diastolic blood pressure of 90 mmHg or higher or the prescription of antihypertensive medication. Obesity was defined as BMI ≥ 25 kg/m^2^ in line with the Asian cut‐off [19]. Elevated triglycerides were defined as plasma levels of ≥ 1.7 mmol/L. Low HDL‐cholesterol was defined as < 0.9 mmol/L for men and < 1.0 mmol/L for women. Albuminuria was diagnosed based on an albumin‐to‐creatinine ratio of ≥ 3 mg/mmol, or a urine albumin excretion rate of ≥ 20 μg/min. The total number of MetS‐related traits for each patient at baseline was calculated using the most recent data recorded prior to the index date. In the post hoc sensitivity analyses, we further examined the associations using a more contemporary definition of MetS based on the International Diabetes Federation (IDF) criteria [16] (Methods S1).

### 2.4 Definition of Baseline Covariates

The following covariates were retrieved based on data recorded at the index date: demographic and anthropometric parameters (age, sex, BMI, waist circumference and smoking status), glycemia‐related parameters (diabetic retinopathy, severe hypoglycaemia and HbA1c levels), key laboratory parameters (including eGFR), Charlson comorbidity index, significant comorbidities (cardiovascular diseases, hyperlipidaemia, chronic kidney disease, chronic obstructive pulmonary disease, liver disease, rheumatoid arthritis and other inflammatory polyarthropathies, history of falls, osteoporosis, hyperparathyroidism and dementia) and baseline use of antidiabetic medications and systemic glucocorticoids (defined by usage in the 6 months prior to the index date). Severe hypoglycaemia, conventionally referring to events requiring external assistance for recovery, was defined in our study as hypoglycaemic episodes requiring hospital attendance or admission [14]. The detailed definitions of disease diagnosis codes are listed in Table S1.

### 2.5 Definition of Outcomes

The study outcomes were incident hip fractures and major osteoporotic fractures. Hip fractures were identified using ICD‐9‐CM code 820, while major osteoporotic fractures were defined as a composite outcome of hip, clinical vertebral and upper limb fracture, identified using ICD‐9‐CM codes 805, 812, 813, 814 or 820. All outcomes were identified in both outpatient and inpatient settings. These diagnosis codes to identify our study outcomes have been validated previously using the same centralized EHRs of the HA with an overall positive predictive value of 96.8% [20].

### 2.6 Statistical Analysis

Baseline characteristics were presented as medians with interquartile ranges (IQR) or as frequencies with percentages, depending on the data type. Incidence rates of fractures (per 1000 person‐years) were calculated by dividing the number of events by the total person‐time at risk. Analyses were further categorized by the number of MetS‐related traits and by individual MetS‐related traits at baseline. Adjusted hazard ratios (aHRs) with 95% confidence intervals (CIs) for fractures were calculated using multivariate Cox regression models stratified by obesity status, accounting for age, sex, smoking status, glycemia‐related parameters, significant comorbidities and use of antidiabetic medications and systemic glucocorticoids. The proportional hazards assumption was examined using Schoenfeld residuals, and no violations were observed. Cause‐specific hazard ratios were calculated to account for competing risks, with individuals censored at the time of death from other causes. Subgroup analyses were stratified by age (≥ 50 vs. < 50 years), sex and baseline osteoporosis to assess the differential influence of albuminuria and MetS traits on the risk of fractures.

As an additional analysis, the index date was shifted to 5 years after the diagnosis of T2D to assess the relative significance of MetS‐related traits and glycaemic control on fracture risk among individuals with diabetes duration of at least 5 years.

Furthermore, nine prespecified and six post hoc sensitivity analyses were conducted to demonstrate the robustness of the results (Methods S1). First, to account for the development of additional MetS‐related traits over time, the number of MetS‐related traits developed within a 1‐month time window during follow‐up was treated as a time‐varying exposure. The 1‐month window was chosen to capture changes in albuminuria and MetS status while minimizing potential bias from irregular follow‐up intervals. Pooled logistic regression was used to explore the association between the number of MetS‐related traits and hip fractures, with corresponding adjusted odds ratios (ORs) used to approximate aHRs after adjusting for baseline covariates. Second, patients with a history of fracture were included. To differentiate incident fractures during follow‐up from ongoing care for prior fractures, a recurrent fracture was defined as the first qualifying fracture recorded after a clean period of at least 90 days without any fracture record following the preceding fracture event [21]. Baseline fracture history was additionally adjusted for in the multivariable models. Third, to account for potential selection bias resulting from the exclusion of patients with missing data, we conducted a sensitivity analysis using inverse probability weighting (IPW) [22]. The probability of having complete data on albuminuria and all MetS components was estimated via logistic regression, accounting for age, sex, pre‐existing comorbidities and use of medications. Weights were truncated at the 1st and 99th percentiles to mitigate undue influence of extreme values. IPW‐weighted Cox models were fitted among patients without missing data to estimate HRs and 95% CIs, with additional adjustment for the baseline characteristics as in the main analysis. Standardized mean differences (SMDs) were used to assess covariate balance, with SMD < 0.2 indicating sufficient balance after weighting [23]. Fourth, the Fine–Gray model was applied to estimate the cumulative incidence of fractures accounting for mortality as a competing risk. Fifth, to address potential misclassification of diabetes diagnosis based solely on a single elevated fasting glucose measurement, we performed a sensitivity analysis excluding the criterion of fasting glucose ≥ 7.0 mmol/L from the diabetes definition. Sixth, to account for heterogeneity in diabetes aetiology and to exclude forms of diabetes not typically associated with MetS, patients with post‐pancreatitis diabetes mellitus (PPDM) were excluded [24]. Seventh, waist circumference was used to define obesity in place of BMI. Eighth, patients with diagnosis of osteoporosis or those using systemic glucocorticoids were excluded to reduce potential confounding from underlying bone conditions or medications that may independently affect fracture risk. Ninth, fracture events accompanied by diagnostic codes for motor vehicle accidents (ICD‐9‐CM E800‐E848) or accidental falls from height (ICD‐9‐CM E880‐E884.1) were excluded to minimize outcome misclassification by removing traumatic fractures. To examine potential dilution of the association among older adults with T2D, we also conducted age‐stratified subgroup analyses (≥ 50 vs. < 50 years) after excluding these trauma‐related fractures.

All statistical analyses were performed using STATA version 16.0 (StataCorp LP, College Station, Texas). Significance was set at a two‐tailed *p*‐value of less than 0.05.

## 3 Results

### 3.1 Baseline Characteristics of the Cohort

A total of 165 289 patients with T2D and complete MetS‐related trait profiles were identified. Among them, 8.3% had only T2D, 22.7% had 2 MetS‐related traits, 34.2% had 3 MetS‐related traits, 23.6% had 4 MetS‐related traits, 9.4% had 5 MetS‐related traits and 1.8% had all 6 MetS‐related traits. Baseline characteristics for the overall cohort and by the number of MetS‐related traits are presented in Tables 1 and S2. The median age was 60.0 years (IQR: 53.0–68.0), and 54.2% were men. The median BMI was 25.8 kg/m^2^ (IQR: 23.3–28.6). The median Charlson Comorbidity Index was 3.0 (IQR: 2.0–4.0). Regarding albuminuria and MetS components, most (78.2%) had concomitant hypertension, 58.2% had obesity, 33.1% had hypertriglyceridemia, 26.7% had albuminuria and 12.0% had low HDL‐cholesterol levels.

**TABLE 1:** Baseline characteristics of study population.

Baseline characteristics	Complete case	Overall
Age at index (years), median (IQR) or *n* (%)	165 289	60.0 (53.0–68.0)
< 50		27 176 (16.4)
50–74		118 796 (71.9)
≥ 75		19 317 (11.7)
Male, *n* (%)	165 289	89 628 (54.2)
MetS‐related traits,^a^ *n* (%)		
Albuminuria	165 289	44 153 (26.7)
Hypertension	165 289	129 310 (78.0)
Hypertriglyceridemia	165 289	54 767 (33.1)
Low levels of HDL‐cholesterol	165 289	19 757 (12.0)
Obesity	165 289	96 251 (58.2)
Number of MetS‐related traits,^a^ *n* (%)	165 289	
1 (T2D)		13 742 (8.3)
2		37 588 (22.7)
3		56 538 (34.2)
4		39 016 (23.6)
5		15 499 (9.4)
6		2906 (1.8)
Lifestyle variables, median (IQR) or *n* (%)		
BMI (kg/m^2^ )	165 289	25.8 (23.3–28.6)
Waist circumference (cm)	94 205	94.0 (90.0–100.0)
Smoking	159 222	23 650 (14.9)
Charlson comorbidity index, median (IQR)	165 289	3.0 (2.0–4.0)
Pre‐existing comorbidities, median (IQR) or *n* (%)		
Cardiovascular disease	165 289	29 425 (17.8)
Hyperlipidaemia	165 289	86 561 (52.4)
Chronic kidney disease	165 289	15 102 (9.1)
Chronic obstructive pulmonary disease	165 289	2656 (1.6)
Liver disease	165 289	484 (0.3)
Rheumatoid arthritis and other inflammatory polyarthropathies	165 289	236 (0.1)
History of falls	165 289	5072 (3.1)
Osteoporosis	165 289	342 (0.2)
Hyperparathyroidism	165 289	120 (0.1)
Dementia	165 289	367 (0.2)
Diabetic retinopathy	165 289	2950 (1.8)
Severe hypoglycaemia	165 289	6933 (4.2)
Use of antidiabetic medications and systemic glucocorticoids, *n* (%)		
Insulin	165 289	5483 (3.3)
Metformin	165 289	105 881 (64.1)
Sulfonylurea	165 289	46 039 (27.9)
Thiazolidinedione	165 289	1152 (0.7)
Dipeptidyl peptidase 4 inhibitors	165 289	2582 (1.6)
SGLT2 inhibitors	165 289	389 (0.2)
Glucagon‐like peptide‐1 receptor agonist	165 289	70 (0.0)
α‐Glucosidase inhibitors	165 289	160 (0.1)
Systemic glucocorticoids	165 289	5514 (3.3)
Clinical parameters,^b^ median (IQR)		
eGFR (mL/min/1.73 m^2^ )	165 112	89.0 (75.1–99.4)
HbA1c (%)	164 599	6.6 (6.2–7.1)
HDL‐cholesterol (mmol/L)	165 289	1.2 (1.0–1.4)
Triglycerides (mmol/L)	165 289	1.3 (1.0–1.9)
Urine albumin‐to‐creatinine ratio (mg/mmol)	165 289	1.3 (0.7–3.2)

Abbreviations: BMI = body mass index; eGFR = estimated glomerular filtration rate; HbA1c = glycated haemoglobin; HDL = high‐density lipoprotein; IQR = interquartile range; MetS = metabolic syndrome; SGLT2 = sodium–glucose cotransporter 2; T2D = type 2 diabetes.

^a^ MetS‐related traits include albuminuria and individual MetS traits (obesity, hypertension, low HDL‐cholesterol and hypertriglyceridemia).

^b^ Clinical parameters were obtained using the most recent measurement prior to the index date.

### 3.2 Incidence of Hip and Major Osteoporotic Fractures

Upon median follow‐up of 5.3 years, 1583 individuals (0.96%) developed incident hip fractures, translating to an incidence rate of 1.81 per 1000 person‐years. The median time to hip fracture was 4.0 years (IQR: 2.1–6.7). Upon median follow‐up of 5.2 years, 3393 individuals (2.05%) developed major osteoporotic fractures, translating to an incidence rate of 3.72 per 1000 person‐years. The median time to major osteoporotic fracture was 3.8 years (IQR: 1.9–6.3).

### 3.3 MetS‐Related Traits and Risk of Fractures

In multivariable analyses, albuminuria and hypertension were associated with an increased risk of hip and major osteoporotic fractures. In contrast, obesity was associated with a significantly lower risk of hip fracture (aHR: 0.51, 95% CI: 0.46–0.57) and major osteoporotic fracture (aHR: 0.71, 95% CI: 0.66–0.77) (Table S3). Therefore, subsequent analyses were stratified by obesity status.

### 3.4 MetS‐Related Traits and Risk of Fractures Stratified by Obesity Status

Among patients without obesity, the presence of additional MetS‐related traits (vs. patients with only T2D) was associated with a significantly increased risk of incident hip fractures (aHR: 1.96, 95% CI: 1.47–2.61) and major osteoporotic fractures (aHR: 1.45, 95% CI: 1.23–1.72) (Table 2). Consistently, each additional MetS‐related trait was associated with an aHR of 1.17 (95% CI: 1.09–1.26) for hip fractures and an aHR of 1.10 (95% CI: 1.04–1.16) for major osteoporotic fractures. Regarding individual MetS‐related traits, albuminuria and hypertension were significant risk factors for hip fractures, with aHRs of 1.54 (95% CI: 1.33–1.78) and 1.36 (95% CI: 1.09–1.69), respectively. Similarly, albuminuria and hypertension were positively associated with major osteoporotic fractures, with aHRs of 1.28 (95% CI: 1.15–1.43) and 1.24 (95% CI: 1.07–1.43), respectively.

**TABLE 2:** Incidences rates and HRs for fractures associated with albuminuria and MetS traits present at baseline in non‐obese patients with T2D.

Outcome	Non‐obese (*N* = 69 038)							
	Event	Incidence rate (per 1000 person‐years)	Crude HR	95% CI	*p*	Adjusted HR^a^	95% CI	*p*
**Hip fracture**								
***Number of MetS‐related traits*** ^b^								
1 (T2D only)	63	0.80	Reference			Reference		
2	368	2.27	2.864	(2.192, 3.741)	< 0.001	1.800	(1.337, 2.423)	< 0.001
3	384	3.67	4.594	(3.519, 5.996)	< 0.001	2.147	(1.588, 2.903)	< 0.001
4	128	4.01	4.966	(3.673, 6.714)	< 0.001	2.191	(1.561, 3.074)	< 0.001
5	23	4.08	5.077	(3.149, 8.184)	< 0.001	1.881	(1.122, 3.151)	0.016
≥ 2^**c**^	903	2.97	3.726	(2.886, 4.811)	< 0.001	1.960	(1.469, 2.614)	< 0.001
Continuous	NA		1.507	(1.415, 1.604)	< 0.001	1.171	(1.087, 1.262)	< 0.001
***Individual MetS‐related traits*** ^b^								
No albuminuria	531	1.82	Reference			Reference		
Albuminuria	435	4.84	2.262	(1.987, 2.575)	< 0.001	1.540	(1.334, 1.778)	< 0.001
No hypertension	121	1.05	Reference			Reference		
Hypertension	845	3.16	2.499	(2.057, 3.035)	< 0.001	1.356	(1.090, 1.686)	0.006
No hypertriglyceridemia	717	2.51	Reference			Reference		
Hypertriglyceridemia	249	2.57	0.950	(0.820, 1.102)	0.498	1.012	(0.864, 1.185)	0.886
No low HDL‐C level	883	2.55	Reference			Reference		
Low HDL‐C level	83	2.35	0.839	(0.666, 1.057)	0.136	0.797	(0.625, 1.017)	0.068
**Major osteoporotic fracture**								
***Number of MetS‐related traits*** ^b^								
1 (T2D only)	180	2.31	Reference			Reference		
2	718	4.47	1.951	(1.657, 2.298)	< 0.001	1.389	(1.164, 1.658)	< 0.001
3	649	6.26	2.723	(2.308, 3.212)	< 0.001	1.570	(1.307, 1.885)	< 0.001
4	201	6.35	2.741	(2.242, 3.352)	< 0.001	1.413	(1.130, 1.767)	0.002
5	41	7.36	3.191	(2.273, 4.480)	< 0.001	1.442	(0.998, 2.085)	0.051
≥ 2^**c**^	1609	5.34	2.324	(1.992, 2.711)	< 0.001	1.452	(1.226, 1.721)	< 0.001
Continuous	NA		1.348	(1.286, 1.413)	< 0.001	1.096	(1.038, 1.158)	0.001
***Individual MetS‐related traits*** ^b^								
No albuminuria	1120	3.86	Reference			Reference		
Albuminuria	669	7.51	1.714	(1.554, 1.891)	< 0.001	1.280	(1.148, 1.428)	< 0.001
No hypertension	299	2.62	Reference			Reference		
Hypertension	1490	5.62	1.921	(1.692, 2.181)	< 0.001	1.238	(1.074, 1.426)	0.003
No hypertriglyceridemia	1320	4.66	Reference			Reference		
Hypertriglyceridemia	469	4.89	0.998	(0.896, 1.112)	0.977	1.016	(0.905, 1.140)	0.791
No low HDL‐C level	1634	4.75	Reference			Reference		
Low HDL‐C level	155	4.43	0.864	(0.730, 1.023)	0.091	0.810	(0.676, 0.970)	0.022

Abbreviations: CI = confidence interval; HbA1c = glycated haemoglobin; HDL = high‐density lipoprotein; HR = hazard ratio; MetS = metabolic syndrome; NA = not available; T2D = type 2 diabetes.

^a^ The models were adjusted by age, sex, smoking status, cardiovascular disease, hyperlipidaemia, chronic kidney disease, chronic obstructive pulmonary disease, liver disease, rheumatoid arthritis and other inflammatory polyarthropathies, history of falls (as a proxy indicator for frailty), osteoporosis, hyperparathyroidism, dementia, diabetic retinopathy, severe hypoglycaemia, use of antidiabetic medications and systemic glucocorticoids and baseline HbA1c level.

^b^ MetS‐related traits include albuminuria and individual MetS traits (obesity, hypertension, low HDL‐cholesterol and hypertriglyceridemia).

^c^ ≥ 2 MetS‐related traits indicate the presence of at least one additional MetS‐related trait beyond T2D.

Among patients with obesity, the relationship between the number of MetS‐related traits and fracture risk was less pronounced (Table 3). There was still a significant trend of increasing hip fracture risk with a higher cumulative number of MetS‐related traits, resulting in an aHR of 1.11 (95% CI: 1.004–1.220) per additional MetS‐related trait. On the other hand, there was no significant trend of increasing major osteoporotic fracture risk with a higher cumulative number of MetS‐related traits. When considering individual MetS‐related traits, albuminuria remained the only significant risk factor for hip fractures (aHR: 1.33, 95% CI: 1.11–1.60) and major osteoporotic fractures (aHR: 1.13, 95% CI: 1.01–1.26).

**TABLE 3:** Incidence rates and HRs for fractures associated with albuminuria and MetS traits present at baseline in obese patients with T2D.

Outcome	Obese (*N* = 96 251)							
	Event	Incidence rate (per 1000 person‐years)	Crude HR	95% CI	*p*	Adjusted HR^a^	95% CI	*p*
**Hip fracture**								
***Number of MetS‐related traits*** ^b^								
2 (T2D + obese)	24	0.51	Reference			Reference		
3	196	0.94	1.887	(1.235, 2.883)	0.003	1.159	(0.709, 1.894)	0.557
4	262	1.41	2.774	(1.826, 4.214)	< 0.001	1.494	(0.916, 2.435)	0.108
5	107	1.31	2.522	(1.620, 3.927)	< 0.001	1.275	(0.758, 2.144)	0.359
6	28	1.77	3.490	(2.023, 6.021)	< 0.001	1.712	(0.909, 3.224)	0.096
≥ 3^**c**^	593	1.21	2.384	(1.585, 3.585)	< 0.001	1.317	(0.817, 2.125)	0.258
Continuous	NA		1.259	(1.161, 1.366)	< 0.001	1.106	(1.004, 1.220)	0.042
***Individual MetS‐related traits*** ^b^								
No albuminuria	351	0.91	Reference			Reference		
Albuminuria	266	1.75	1.738	(1.479, 2.043)	< 0.001	1.332	(1.111, 1.596)	0.002
No hypertension	43	0.47	Reference			Reference		
Hypertension	574	1.29	2.527	(1.848, 3.456)	< 0.001	1.246	(0.872, 1.781)	0.228
No hypertriglyceridemia	391	1.20	Reference			Reference		
Hypertriglyceridemia	226	1.07	0.834	(0.705, 0.987)	0.034	0.904	(0.750, 1.091)	0.292
No low HDL‐C level	530	1.15	Reference			Reference		
Low HDL‐C level	87	1.12	0.982	(0.778, 1.238)	0.876	1.113	(0.863, 1.435)	0.410
**Major osteoporotic fracture**								
***Number of MetS‐related traits*** ^b^								
2 (T2D + obese)	100	2.14	Reference			Reference		
3	576	2.79	1.318	(1.066, 1.629)	0.011	0.925	(0.737, 1.160)	0.498
4	622	3.38	1.583	(1.281, 1.955)	< 0.001	1.022	(0.814, 1.284)	0.849
5	250	3.07	1.427	(1.132, 1.799)	0.003	0.925	(0.719, 1.190)	0.546
6	56	3.56	1.672	(1.205, 2.319)	0.002	0.999	(0.695, 1.437)	0.997
≥ 3^**c**^	1504	3.09	1.448	(1.182, 1.773)	< 0.001	0.963	(0.774, 1.197)	0.734
Continuous	NA		1.104	(1.049, 1.162)	< 0.001	1.010	(0.953, 1.071)	0.735
***Individual MetS‐related traits*** ^b^								
No albuminuria	1040	2.71	Reference			Reference		
Albuminuria	564	3.74	1.310	(1.180, 1.453)	< 0.001	1.128	(1.007, 1.264)	0.037
No hypertension	181	1.98	Reference			Reference		
Hypertension	1423	3.22	1.562	(1.336, 1.827)	< 0.001	0.973	(0.820, 1.155)	0.757
No hypertriglyceridemia	1009	3.12	Reference			Reference		
Hypertriglyceridemia	595	2.83	0.891	(0.803, 0.988)	0.029	0.934	(0.835, 1.045)	0.234
No low HDL‐C level	1392	3.05	Reference			Reference		
Low HDL‐C level	212	2.74	0.912	(0.786, 1.057)	0.221	1.006	(0.858, 1.179)	0.941

Abbreviations: CI = confidence interval; HbA1c = glycated haemoglobin; HDL = high‐density lipoprotein; HR = hazard ratio; MetS = metabolic syndrome; NA = not available; T2D = type 2 diabetes.

^a^ The models were adjusted by age, sex, smoking status, cardiovascular disease, hyperlipidaemia, chronic kidney disease, chronic obstructive pulmonary disease, liver disease, rheumatoid arthritis and other inflammatory polyarthropathies, history of falls (as a proxy indicator for frailty), osteoporosis, hyperparathyroidism, dementia, diabetic retinopathy, severe hypoglycaemia, use of antidiabetic medications and systemic glucocorticoids and baseline HbA1c level.

^b^ MetS‐related traits include albuminuria and individual MetS traits (obesity, hypertension, low HDL‐cholesterol and hypertriglyceridemia).

^c^ ≥ 3 MetS‐related traits indicate at least one additional MetS‐related trait beyond T2D and obesity.

### 3.5 Subgroup Analyses

Analyses stratified by age (Tables S4 and S5), sex (Tables S6 and S7) and baseline osteoporosis (Table S8) were generally consistent with the main analyses, except for younger adults, where results were not significant likely due to the small sample size. The presence of additional MetS‐related traits was significantly associated with an increased risk of incident hip and major osteoporotic fractures among patients without obesity, as shown in the sex‐stratified analysis. Albuminuria was a significant predictor of hip fractures across all patient groups, except among obese female patients.

### 3.6 Additional Analyses

We evaluated the differential effects of albuminuria and glycaemic control by shifting the index date to 5 years after the diagnosis of T2D (Table S9). Regardless of the duration of diabetes, albuminuria remained a significant risk factor for incident hip and major osteoporotic fractures. On the other hand, suboptimal glycaemic control (defined as HbA1c ≥ 9%) was less consistent as a risk factor for incident hip and major osteoporotic fractures. When we considered the entire cohort, suboptimal glycaemic control was associated with a higher incident major osteoporotic fracture risk but not hip fracture. It was when we considered the subcohort with duration of diabetes ≥ 5 years that suboptimal glycaemic control was consistently associated with increased risk of incident hip fractures (aHR: 1.30, 95% CI: 1.12–1.50) and incident major osteoporotic fractures (aHR 1.20, 95% CI: 1.09–1.34).

We further examined the association between MetS‐related traits and incident fractures while accounting for the development of additional MetS‐related traits during follow‐up. The results remained consistent, showing an increased aHR for incident hip fractures and major osteoporotic fractures mainly in the non‐obese group (Table S10). To assess potential selection bias due to missing data, we applied IPW. After weighting, all SMDs were < 0.2, indicating that baseline characteristics were balanced (Table S11). The IPW‐weighted estimates were consistent with those from the main analysis (Table S12). Additional sensitivity analyses yielded results largely consistent with the main findings. These included (i) inclusion of patients with a history of fracture; (ii) accounting for death as a competing risk using the Fine–Gray model; (iii) removal of the criterion of fasting glucose ≥ 7.0 mmol/L from the diabetes definition; (iv) exclusion of patients with PPDM; (v) redefinition of obesity using waist circumference instead of BMI; (vi) exclusion of patients with osteoporosis or those using systemic glucocorticoids; and (vii) exclusion of fracture events accompanied by diagnostic codes for motor vehicle accidents or falls from height, and further stratifying analyses by age (Tables S13–S19). The results of a further six post hoc sensitivity analyses supported the robustness of the findings (Tables S20–S26).

## 4 Discussion

We evaluated a population‐based cohort of 165 289 patients with T2D who were well‐characterized for albuminuria status and MetS traits and were at the early stage of diabetes diagnosis (median duration of diabetes of 1 year). MetS traits carry a differential impact on patients who did and did not have comorbid obesity. Nonetheless, regardless of the presence of concomitant obesity, albuminuria was the most consistent risk factor for incident hip fractures and major osteoporotic fractures. More importantly, we identified that in the early stage of diabetes diagnosis, albuminuria—but not suboptimal glycaemic control—was the independent predictor of incident hip fractures. In the era of increasing awareness of the cardiovascular‐kidney‐metabolic syndrome overlap [25], our study sheds light on the importance of addressing albuminuria and various MetS components to optimize bone health in patients with T2D. Our study also highlighted the potential key role of screening for albuminuria for fracture risk stratification upon diagnosis of diabetes, akin to screening for albuminuria for heart failure or kidney risk stratification in individuals with T2D [26].

### 4.1 Clinical Implications of Study Findings

Individuals with T2D have an elevated risk of fractures at all sites, but the fracture risks tend to be underestimated by the currently available tools such as the FRAX and the bone mineral density (BMD) measurements by dual‐energy x‐ray absorptiometry (DXA) [4]. Several strategies to improve the fracture risk stratification in T2D have been proposed: (i) adjustment of certain factors, such as increasing the age input, the inclusion of ‘rheumatoid arthritis’ as proxy and downward adjustment of BMD T‐score; or (ii) inclusion of trabecular bone score (an indirect index of bone microarchitecture, which can be obtained from DXA images). Moreover, the release of FRAXplus beta‐version [27] represents the collaborative effort of the scientific community to improve fracture risk stratification in T2D by including several diabetes‐related factors, such as the presence of T2D and the duration of T2D. Our study sheds light on the relevance of albuminuria and MetS traits for fracture risks in individuals with T2D, as we analysed both individual MetS‐related traits and their cumulative number in relation to the risk of fracture, demonstrating the potential for specific MetS‐related traits to further refine fracture risk stratification in T2D beyond established risk factors such as age, sex and prior fracture history. Given that the pandemic likely affected healthcare utilization [28], and our prior work indicates people with diabetes and COVID‐19 may have an elevated risk of major osteoporotic fractures and falls [29], we conducted a sensitivity analysis excluding the pandemic period; results were largely consistent with the main analysis, supporting the robustness of our conclusions.

### 4.2 Comparison With Existing Studies on MetS and Fracture Risk

Existing studies mainly focused on the MetS and fracture risk in the general population. A recent systematic review and meta‐analysis of 20 studies (cross‐sectional and cohort) of the associations between MetS and fracture risk showed a lack of association in the general population, likely because MetS is not a single pathologic entity but a cluster of components with possibly opposing effects on bone health [10]. Our study supports this concept by demonstrating a differential impact of albuminuria and individual MetS traits on fracture risks.

For example, obesity was associated with a lower risk of fractures, which may reflect both biomechanical factors, such as greater soft tissue padding that dissipates impact forces, and the higher BMD often seen in obesity, temporarily reducing fracture susceptibility. Differences in fat distribution may also contribute, as greater subcutaneous fat and muscle mass could mitigate fall impact and provide mechanical protection [11]. Because BMI reflects overall body mass rather than central adiposity, reliance on BMI alone may have insufficiently captured central fat distribution and misclassified some higher‐risk patients as non‐obese. Accordingly, we conducted a sensitivity analysis defining obesity by waist circumference, and the results indicated that central adiposity may be more strongly associated with metabolic dysregulation and skeletal fragility than overall body mass, particularly among non‐obese patients. Additionally, competing risks, such as higher mortality among obese patients with severe metabolic abnormalities, may have contributed to this attenuation, as evidenced by our competing risk analyses in which the associations were further weakened. This pattern is consistent with the so‐called ‘obesity paradox’ in fracture epidemiology, where adiposity appears to mitigate the adverse skeletal effects of metabolic disturbances. However, this likely reflects effect modification through biomechanical and hormonal pathways rather than a true protective effect of obesity. Nonetheless, obesity may still impair bone quality through inflammation and insulin resistance, and its apparent protective effect should therefore be interpreted with caution.

In contrast, albuminuria, a marker of microvascular and renal complications, was associated with increased fracture risk, likely reflecting impaired bone quality. Stratifying the cohort by obesity status provided additional insights, as individuals without obesity may be more immediately exposed to the deleterious skeletal effects of metabolic disturbances such as albuminuria and other diabetes‐related complications, highlighting the value of evaluating albuminuria and individual MetS traits, rather than MetS as a whole, for predicting fracture risk in individuals with T2D.

### 4.3 Graded Increase in Fracture Risk With Increasing Number of MetS‐Related Traits

Our study demonstrated a graded increase in fracture risk with the cumulative number of MetS‐related traits, which was more prominent among individuals without obesity. Although not every MetS‐related trait was associated with a statistically significant increase in fracture risk, each likely carried additive effects. In our cohort, the most significant factor for fractures among the MetS‐related traits in individuals with T2D was albuminuria. The positive association between albuminuria and fracture risk is likely explained by albuminuria being a marker of microvascular disease, which plays an important role in bone physiology [30, 31]. The other significant MetS‐related trait for fracture was hypertension, especially among individuals without obesity. This is consistent with a recent systematic review and meta‐analysis of 28 studies reporting increased odds of osteoporotic fractures with hypertension (pooled OR = 1.33) [32]. A postulated mechanism is the associated abnormalities in calcium metabolism such as an increase in urinary calcium excretion. Although some individual MetS‐related traits, namely, triglycerides and low HDL‐cholesterol, did not show a statistically significant association with fracture risk, the higher fracture risk with an increasing number of MetS‐related traits could suggest an increasing degree of insulin resistance, which in turn is associated with an increase in fracture risk via a complex interaction impairing the bone quality and altering the bone material properties [33]. Last but not least, the fact that the presence of obesity attenuated—but not nullified—the association between MetS‐related traits and fracture suggested that the assessment and management of other MetS‐related traits are still crucial in optimizing bone health among individuals with obesity.

### 4.4 Role of Albuminuria in Fracture Risk in T2D

Our study revealed that albuminuria remained a consistent and significant risk factor for both hip and major osteoporotic fractures early in the diagnosis of T2D, in contrast to glycaemic control, which became more of a consistent risk factor for fractures later in the disease course. This may be because albuminuria reflects cumulative microvascular and endothelial injury that develops early in diabetes and directly affects skeletal integrity [34]. Elevated urinary albumin excretion indicates systemic microvascular dysfunction, inflammation and oxidative stress, which can impair bone perfusion and remodelling and are often correlated with neuropathy, sarcopenia and frailty, all important determinants of fall‐related fractures [34]. In sensitivity analyses where albuminuria was classified by severity (micro‐ and macroalbuminuria vs. normoalbuminuria), a dose–response relationship between albuminuria severity and fracture risk was observed. This pattern aligns with a biological pathway linking albuminuria to skeletal fragility. By contrast, hyperglycaemia contributes to skeletal fragility more gradually through the accumulation of advanced glycation end‐products, altered collagen cross‐linking and reduced osteoblast activity, supported by our finding that elevated HbA1c predicted fractures after 5 years of diabetes [35]. This is in line with recent studies, which suggest no significant increase in fracture risk in those with recently diagnosed diabetes [36]. Future studies incorporating repeated HbA1c measurements could help clarify its time‐varying effects on the associations of albuminuria and MetS traits with fracture risk. The key message is that upon diagnosis of T2D, patients should strive to maintain optimal glycaemic control to minimize the impact of persistent hyperglycaemia on the bone microarchitecture and material properties.

On the other hand, our results highlighted the potential relevance of early screening of albuminuria in fracture risk stratification, adding to the list of benefits of albuminuria screening [37]. Albuminuria may be incorporated into a FRAX‐like fracture risk prediction model to enhance risk prediction in patients with T2D. When we applied the contemporary IDF definition of MetS (which does not include albuminuria as one of the MetS traits), the associations between MetS traits and fracture risk were attenuated. Reintroducing albuminuria as a MetS‐related trait within the IDF framework yielded associations consistent with the main analysis, highlighting the pivotal role of albuminuria in linking metabolic dysregulation to skeletal fragility in T2D. These findings suggested that defining MetS without albuminuria might underestimate the impact of metabolic dysfunction on bone health in patients with T2D, among whom albuminuria was both common and clinically relevant.

### 4.5 Strengths and Limitations

In this large population‐based cohort, we revealed the predictive ability of individual MetS‐related traits and their cumulative number for fracture risks in T2D, potentially refining fracture risk stratification in this at‐risk population. The strength of our study lies in the large sample size, which provides sufficient power to study the association of albuminuria and MetS traits with incident hip fractures and major osteoporotic fractures. We observed that hip fractures accounted for a higher proportion of fractures than typically reported in people with T2D, likely reflecting the nature of electronic health record data, where hip fractures are more reliably captured [38]. However, this study has a few limitations. First, the participants included were predominantly ethnic Chinese and older adults (≥ 60 years), which may limit generalizability. Differences in the phenotype of T2D in Asians compared to Caucasians and ethnic differences in the fracture epidemiology [39], together with the higher comorbidity burden, frailty and fall rates in older adults, mean that the observed associations may primarily reflect risk patterns in older Asian populations. While this age distribution reflects the general T2D population in Hong Kong, future studies that include a broader age range or diverse ethnic groups are warranted. Second, BMD measurements were unavailable, representing a key limitation as BMD is a major determinant of fracture risk. Without BMD data, we could not assess whether the observed associations were mediated through changes in bone mass or bone quality. To partially address bone‐related confounding, we additionally adjusted for vitamin D and calcium supplement use in sensitivity analysis, yielding results consistent with the main analysis. Third, despite our efforts to include a wide range of baseline comorbidities, laboratory results and medication use in the multivariable analyses, potential residual confounding factors could not be entirely excluded. For example, direct measurements of vitamin D and parathyroid hormone were not available, limiting the assessment of calcium–phosphate homeostasis abnormalities that may affect bone health. Although secondary hyperparathyroidism was identified using diagnostic codes, it is likely under‐ascertained in routine clinical data. Moreover, information on physical activity was not available, and a history of falls was therefore considered as a proxy instead. Future studies incorporating densitometric parameters (e.g., BMD), vitamin D levels and physical activity would help to elucidate underlying mechanisms and provide more robust estimates of the associations of interest. Fourth, we could not reliably distinguish other forms of adult‐onset diabetes not typically associated with MetS, such as latent autoimmune diabetes in adults (LADA); however, excluding patients with PPDM did not alter the results.

## 5 Conclusion

In this large population‐based cohort of patients with T2D predominantly of Asian descent from Hong Kong, albuminuria emerged as an important predictor of fracture risk. The presence of MetS traits compounds the risk, especially in non‐obese patients with T2D. These findings could be instrumental in shaping screening initiatives for fracture risk optimization in T2D.

## Conflicts of Interest

The authors declare no conflicts of interest.

## Supporting information


**Methods S1:** Post hoc sensitivity analyses.

**Table S1:** Definition of disease diagnosis coding.
**Table S2:** Baseline characteristics of the study population by the number of metabolic syndrome‐related traits.
**Table S3:** Incidence rates and HRs for fractures associated with albuminuria and MetS traits present at baseline in patients with T2D.
**Table S4:** Incidence rates and HRs for fractures associated with albuminuria and MetS traits present at baseline in obese and non‐obese older adults with T2D.
**Table S5:** Incidence rates and HRs for fractures associated with albuminuria and MetS traits present at baseline in obese and non‐obese young adults with T2D.
**Table S6:** Incidence rates and HRs for fractures associated with albuminuria and MetS traits present at baseline in obese and non‐obese male patients with T2D.
**Table S7:** Incidence rates and HRs for fractures associated with albuminuria and MetS traits present at baseline in obese and non‐obese female patients with T2D.
**Table S8:** Incidence rates and HRs for fractures associated with albuminuria and MetS traits present at baseline in obese and non‐obese patients with T2D, stratified by osteoporosis status.
**Table S9:** Incidence rates and HRs for fractures associated with individual MetS‐related traits by time since T2D diagnosis.
**Table S10:** Risk of fractures evaluated using pooled logistic regression by obese status, accounting for the development of additional MetS‐related traits over time.
**Table S11:** Baseline characteristics of patients with complete and incomplete data before and after weighting.
**Table S12:** Incidence rates and HRs for fractures associated with albuminuria and MetS traits present at baseline in obese and non‐obese patients with T2D, weighted by inverse probability of having complete baseline MetS data.
**Table S13:** Incidence rates and HRs for fractures associated with albuminuria and MetS traits present at baseline in obese and non‐obese patients with T2D, including patients with prior fracture history.
**Table S14:** Competing risk regression analyses for fractures associated with the albuminuria and MetS traits present at baseline in obese and non‐obese patients with T2D.
**Table S15:** Incidence rates and HRs for fractures associated with albuminuria and MetS traits present at baseline in obese and non‐obese patients with T2D, excluding patients diagnosed solely by a fasting glucose ≥ 7.0 mmol/L.
**Table S16:** Incidence rates and HRs for fractures associated with albuminuria and MetS traits present at baseline in obese and non‐obese patients with T2D, excluding those with post‐pancreatitis diabetes mellitus.
**Table S17:** Incidence rates and HRs for fractures associated with albuminuria and MetS traits present at baseline in obese and non‐obese patients with T2D, where obesity is defined by waist circumference.
**Table S18:** Incidence rates and HRs for fractures associated with albuminuria and MetS traits present at baseline in obese and non‐obese patients with T2D, excluding those with a diagnosis of osteoporosis or systemic glucocorticoid use.
**Table S19:** Incidence rates and HRs for fractures associated with albuminuria and MetS traits present at baseline in obese and non‐obese patients with T2D, overall and stratified by age, excluding fracture events accompanied by diagnostic codes for motor vehicle accidents or accidental falls from height.
**Table S20:** Incidence rates and HRs for fractures associated with albuminuria and MetS traits present at baseline in obese and non‐obese patients with T2D, excluding the COVID‐19 pandemic period.
**Table S21:** Incidence rates and HRs for fractures associated with albuminuria and MetS traits present at baseline in obese and non‐obese patients with T2D, with additional adjustment for diabetes duration.
**Table S22:** Incidence rates and HRs for fractures associated with albuminuria and MetS traits present at baseline in obese and non‐obese patients with T2D, with additional adjustment for neuropathy and the use of calcium and vitamin D supplements.
**Table S23:** Incidence rates and HRs for fractures associated with albuminuria and MetS traits present at baseline in obese and non‐obese patients with T2D, using the definition of MetS proposed by the International Diabetes Federation.
**Table S24:** Incidence rates and HRs for fractures associated with albuminuria and MetS traits present at baseline in obese and non‐obese patients with T2D, using the definition of MetS proposed by the International Diabetes Federation and adding albuminuria as an additional component of MetS.
**Table S25:** Incidence rates and HRs for fractures associated with albuminuria and MetS traits present at baseline in obese and non‐obese patients with T2D, with albuminuria defined by the two most recent urine measurements within 1 year before the index date.
**Table S26:** Incidence rates and HRs for fractures associated with albuminuria and MetS traits present at baseline in obese and non‐obese patients with T2D, categorized by albuminuria severity.

## Data Availability

The data that support the findings of this study were provided by the Hong Kong HA. Restrictions apply to the availability of these data, which were used under licence for this study.
