# Supplementary material for: Associations of Albuminuria and Metabolic Syndrome Traits With Fracture Risk in Patients With Type 2 Diabetes: A Population‐Based Cohort Study
Source: J Cachexia Sarcopenia Muscle. 2026 Feb 2;17(1):e70215. doi: 10.1002/jcsm.70215 (PMC12864539; doi:10.1002/jcsm.70215)
Supplement: Supplementary file 2 — Table S1: Definition of disease diagnosis coding. Table S2: Baseline characteristics of the study population by the number of metabolic syndrome‐related traits. Table S3: Incidence rates and HRs for fractures associated with albuminuria and MetS traits present at baseline in patients with T2D. Table S4: Incidence rates and HRs for fractures associated with albuminuria and MetS traits present at baseline in obese and non‐obese older adults with T2D. Table S5: Incidence rates and HRs for fractures associated with albuminuria and MetS traits present at baseline in obese and non‐obese young adults with T2D. Table S6: Incidence rates and HRs for fractures associated with albuminuria and MetS traits present at baseline in obese and non‐obese male patients with T2D. Table S7: Incidence rates and HRs for fractures associated with albuminuria and MetS traits present at baseline in obese and non‐obese female patients with T2D. Table S8: Incidence rates and HRs for fractures associated with albuminuria and MetS traits present at baseline in obese and non‐obese patients with T2D, stratified by osteoporosis status. Table S9: Incidence rates and HRs for fractures associated with individual MetS‐related traits by time since T2D diagnosis. Table S10: Risk of fractures evaluated using pooled logistic regression by obese status, accounting for the development of additional MetS‐related traits over time. Table S11: Baseline characteristics of patients with complete and incomplete data before and after weighting. Table S12: Incidence rates and HRs for fractures associated with albuminuria and MetS traits present at baseline in obese and non‐obese patients with T2D, weighted by inverse probability of having complete baseline MetS data. Table S13: Incidence rates and HRs for fractures associated with albuminuria and MetS traits present at baseline in obese and non‐obese patients with T2D, including patients with prior fracture history. Table S14: Competing risk regression analyses for fr [file JCSM-17-e70215-s001.docx]

**Full title:** Associations of Albuminuria and Metabolic Syndrome Traits with Fracture Risk in Patients with Type 2 Diabetes: A Population-Based Cohort Study

**Authors:** Xi Xiong^1,2,3,4,*^, David Tak Wai Lui^5,6,*,#^, Chengsheng Ju^3^, Xiaodong Liu^2,7^, Li Wei^2,3,8^, Manju Chandran^6,9,10^, Carlos King Ho Wong^2,11,12,#^

**Supplementary materials**

**Contents**

[Supplementary Methods. Post hoc sensitivity analyses. 3](#_Toc218435261)

[Supplementary Table 1. Definition of disease diagnosis coding. 4](#_Toc218435262)

[Supplementary Table 2. Baseline characteristics of the study population by the number of metabolic syndrome-related traits. 5](#_Toc218435263)

[Supplementary Table 3. Incidence rates and HRs for fractures associated with albuminuria and MetS traits present at baseline in patients with T2D. 7](#_Toc218435264)

[Supplementary Table 4. Incidence rates and HRs for fractures associated with albuminuria and MetS traits present at baseline in obese and non-obese older adults with T2D. 8](#_Toc218435265)

[Supplementary Table 5. Incidence rates and HRs for fractures associated with albuminuria and MetS traits present at baseline in obese and non-obese young adults with T2D. 10](#_Toc218435266)

[Supplementary Table 6. Incidence rates and HRs for fractures associated with albuminuria and MetS traits present at baseline in obese and non-obese male patients with T2D. 11](#_Toc218435267)

[Supplementary Table 7. Incidence rates and HRs for fractures associated with albuminuria and MetS traits present at baseline in obese and non-obese female patients with T2D. 13](#_Toc218435268)

[Supplementary Table 8. Incidence rates and HRs for fractures associated with albuminuria and MetS traits present at baseline in obese and non-obese patients with T2D, stratified by osteoporosis status. 15](#_Toc218435269)

[Supplementary Table 9. Incidence rates and HRs for fractures associated with individual MetS-related traits by time since T2D diagnosis. 18](#_Toc218435270)

[Supplementary Table 10. Risk of fractures evaluated using pooled logistic regression by obese status, accounting for the development of additional MetS-related traits over time. 20](#_Toc218435271)

[Supplementary Table 11. Baseline characteristics of patients with complete and incomplete data before and after weighting. 21](#_Toc218435272)

[Supplementary Table 12. Incidence rates and HRs for fractures associated with albuminuria and MetS traits present at baseline in obese and non-obese patients with T2D, weighted by inverse probability of having complete baseline MetS data. 22](#_Toc218435273)

[Supplementary Table 13. Incidence rates and HRs for fractures associated with albuminuria and MetS traits present at baseline in obese and non-obese patients with T2D, including patients with prior fracture history. 24](#_Toc218435274)

[Supplementary Table 14. Competing risk regression analyses for fractures associated with the albuminuria and MetS traits present at baseline in obese and non-obese patients with T2D. 26](#_Toc218435275)

[Supplementary Table 15. Incidence rates and HRs for fractures associated with albuminuria and MetS traits present at baseline in obese and non-obese patients with T2D, excluding patients diagnosed solely by a fasting glucose ≥7.0 mmol/L. 28](#_Toc218435276)

[Supplementary Table 16. Incidence rates and HRs for fractures associated with albuminuria and MetS traits present at baseline in obese and non-obese patients with T2D, excluding those with post-pancreatitis diabetes mellitus. 30](#_Toc218435277)

[Supplementary Table 17. Incidence rates and HRs for fractures associated with albuminuria and MetS traits present at baseline in obese and non-obese patients with T2D, where obesity is defined by waist circumference. 32](#_Toc218435278)

[Supplementary Table 18. Incidence rates and HRs for fractures associated with albuminuria and MetS traits present at baseline in obese and non-obese patients with T2D, excluding those with a diagnosis of osteoporosis or systemic glucocorticoid use. 34](#_Toc218435279)

[Supplementary Table 19. Incidence rates and HRs for fractures associated with albuminuria and MetS traits present at baseline in obese and non-obese patients with T2D, overall and stratified by age, excluding fracture events accompanied by diagnostic codes for motor vehicle accidents or accidental falls from height. 36](#_Toc218435280)

[Supplementary Table 20. Incidence rates and HRs for fractures associated with albuminuria and MetS traits present at baseline in obese and non-obese patients with T2D, excluding the COVID-19 pandemic period. 40](#_Toc218435281)

[Supplementary Table 21. Incidence rates and HRs for fractures associated with albuminuria and MetS traits present at baseline in obese and non-obese patients with T2D, with additional adjustment for diabetes duration. 42](#_Toc218435282)

[Supplementary Table 22. Incidence rates and HRs for fractures associated with albuminuria and MetS traits present at baseline in obese and non-obese patients with T2D, with additional adjustment for neuropathy and the use of calcium and vitamin D supplements. 44](#_Toc218435283)

[Supplementary Table 23. Incidence rates and HRs for fractures associated with albuminuria and MetS traits present at baseline in obese and non-obese patients with T2D, using the definition of MetS proposed by the International Diabetes Federation. 46](#_Toc218435284)

[Supplementary Table 24. Incidence rates and HRs for fractures associated with albuminuria and MetS traits present at baseline in obese and non-obese patients with T2D, using the definition of MetS proposed by the International Diabetes Federation and adding albuminuria as an additional component of MetS. 48](#_Toc218435285)

[Supplementary Table 25. Incidence rates and HRs for fractures associated with albuminuria and MetS traits present at baseline in obese and non-obese patients with T2D, with albuminuria defined by the two most recent urine measurements within one year before the index date. 50](#_Toc218435286)

[Supplementary Table 26. Incidence rates and HRs for fractures associated with albuminuria and MetS traits present at baseline in obese and non-obese patients with T2D, categorised by albuminuria severity. 52](#_Toc218435287)

## Supplementary Methods. Post hoc sensitivity analyses.

We conducted a series of post hoc sensitivity analyses to assess the robustness of our findings. First, to mitigate the unpredictable impact of COVID-19, we repeated the analyses with the end of follow-up date of December 31, 2019. Second, we additionally adjusted for T2D duration as a continuous variable. Third, we further adjusted for neuropathy and the use of calcium and vitamin D supplements. Fourth, we defined MetS traits using the International Diabetes Federation criteria [1] and incorporated albuminuria as a MetS-related trait in an exploratory analysis. Fifth, to account for variability, we restricted the analyses to patients with at least two urine measurements within one year before the index date, defining albuminuria when both of the two most recent measurements exceeded the corresponding threshold. Sixth, to address threshold considerations, we categorised albuminuria as macroalbuminuria (albumin-to-creatinine ratio [ACR] >34 mg/mmol or urine albumin excretion rate [AER] >200 μg/min), microalbuminuria (ACR ≥3 to ≤34 mg/mmol or AER ≥20 to ≤200 μg/min), or normoalbuminuria (ACR <3 mg/mmol or AER <20 μg/min), and repeated the analyses.

Reference:

1. Alberti KG, Zimmet P, Shaw J. The metabolic syndrome--a new worldwide definition. Lancet. 2005;366:1059-62. doi:10.1016/s0140-6736(05)67402-8

## Supplementary Table 1. Definition of disease diagnosis coding.

| **Diseases** | **ICD-9-CM**  **diagnosis codes** | **ICD-9-CM procedure codes** | **BNF codes or drug name** | **Clinical parameters** |
| --- | --- | --- | --- | --- |
| Cardiovascular disease | 398.91, 401-405, 410, 412, 425.4, 425.5, 425.7-425.9, 427.3, 428, 433, 434, 436-438, 453.8 |  |  |  |
| Hyperlipidemia | 272.0-272.4 |  |  |  |
| Chronic kidney disease | 403-404, 582, 585, 590.0 |  |  | Estimated glomerular filtration rate < 60mL/min/1.73m^2^ |
| Chronic obstructive pulmonary disease | 490-496, 500-505, 506.4 |  |  |  |
| Liver disease | 456.0-456.2, 571.2, 571.4-572.6, 572.2-572.4, 572.8 |  |  |  |
| Rheumatoid arthritis and other inflammatory polyarthropathies | 710.0, 710.1, 710.4, 714.0-714.2, 714.81, 725 |  |  |  |
| History of falls | 781.2, 781.3, 781.99, V15.88, E880-E888 |  |  |  |
| Osteoporosis | 733.0 |  | BNF: 6.6.2;  Drug name: raloxifene, denosumab, teriparatide, etidronate, clodronate, pamidronate (nitrogenous), alendronate, ibandronate, risedronate, zoledronate |  |
| Hyperparathyroidism |  | 06.81, 06.89 |  |  |
| Dementia | 290, 294.1, 294.2, 331.0, 331.1, 331.82 |  |  |  |
| Diabetic retinopathy | 249.5, 362.01-362.07 |  |  |  |
| Severe hypoglycemia | 250.3, 250.8, 251.0-251.2, 270.3, 775.0, 775.6, 962.3 |  |  |  |

Notes: ICD-9-CM = International Classification of Diseases, Ninth Revision, Clinical Modification; BNF = British National Formulary**.**

## Supplementary Table 2. Baseline characteristics of the study population by the number of metabolic syndrome-related traits.

| **Baseline characteristics** | **1 MetS-related trait (T2D)**^*^ | **2 MetS-related traits**^*^ | **3 MetS-related traits**^*^ | **4 MetS-related traits**^*^ | **5 MetS-related traits**^*^ | **6 MetS-related traits**^*^ |
| --- | --- | --- | --- | --- | --- | --- |
|  | **(N = 13,742)** | **(N = 37,588)** | **(N = 56,538)** | **(N = 39,016)** | **(N = 15,499)** | **(N = 2,906)** |
| Age at index (years), median (IQR) or n (%) | 57.0 (51.0–63.0) | 61.0 (54.0–68.0) | 61.0 (54.0–69.0) | 60.0 (53.0–68.0) | 59.0 (51.0–67.0) | 57.0 (48.0–66.0) |
| < 50 | 2,976 (21.7) | 5,457 (14.5) | 7,980 (14.1) | 6,553 (16.8) | 3,392 (21.9) | 818 (28.2) |
| 50-74 | 10,275 (74.8) | 27,611 (73.5) | 41,269 (73.0) | 27,530 (70.6) | 10,310 (66.5) | 1,801 (62.0) |
| ≥ 75 | 491 (3.6) | 4,520 (12.0) | 7,289 (12.9) | 4,933 (12.6) | 1,797 (11.6) | 287 (9.9) |
| Male, n (%) | 7,422 (54.0) | 20,536 (54.6) | 30,504 (54.0) | 20,961 (53.7) | 8,486 (54.8) | 1,719 (59.2) |
| MetS-related traits^*^, n (%) |  |  |  |  |  |  |
| Albuminuria | 0 (0.0) | 1,345 (3.6) | 11,180 (19.8) | 17,766 (45.5) | 10,956 (70.7) | 2,906 (100.0) |
| Hypertension | 0 (0.0) | 24,412 (64.9) | 49,852 (88.2) | 36,889 (94.5) | 15,251 (98.4) | 2,906 (100.0) |
| Hypertriglyceridemia | 0 (0.0) | 2,948 (7.8) | 11,945 (21.1) | 22,781 (58.4) | 14,187 (91.5) | 2,906 (100.0) |
| Low levels of HDL-cholesterol | 0 (0.0) | 610 (1.6) | 2,747 (4.9) | 6,379 (16.3) | 7,115 (45.9) | 2,906 (100.0) |
| Obesity | 0 (0.0) | 8,273 (22.0) | 37,352 (66.1) | 33,233 (85.2) | 14,487 (93.5) | 2,906 (100.0) |
| Lifestyle variables, median (IQR) or n (%) |  |  |  |  |  |  |
| BMI (kg/m^2^) | 22.4 (20.8–23.7) | 23.5 (21.9–24.8) | 26.2 (24.0–28.8) | 27.6 (25.7–30.1) | 28.4 (26.4–31.1) | 29.0 (26.9–31.8) |
| Waist circumference (cm) | 86.0 (82.0–91.0) | 90.0 (85.0–94.0) | 94.0 (90.0–100.0) | 96.0 (91.0–102.0) | 97.0 (92.0–103.0) | 98.0 (93.0–105.0) |
| Smoking | 2,184 (16.3) | 5,182 (14.2) | 7,326 (13.4) | 5,657 (15.1) | 2,684 (18.2) | 617 (22.1) |
| Charlson comorbidity index, median (IQR) | 2.0 (2.0–3.0) | 3.0 (2.0–4.0) | 3.0 (2.0–4.0) | 3.0 (2.0–4.0) | 3.0 (2.0–4.0) | 3.0 (2.0–4.0) |
| Pre-existing comorbidities, median (IQR) or n (%) | |  |  |  |  |  |
| Cardiovascular disease | 142 (1.0) | 4,844 (12.9) | 10,786 (19.1) | 8,888 (22.8) | 3,893 (25.1) | 872 (30.0) |
| Hyperlipidemia | 5,799 (42.2) | 19,077 (50.8) | 30,493 (53.9) | 21,401 (54.9) | 8,292 (53.5) | 1,499 (51.6) |
| Chronic kidney disease | 184 (1.3) | 2,052 (5.5) | 5,268 (9.3) | 4,670 (12.0) | 2,357 (15.2) | 571 (19.6) |
| Chronic obstructive pulmonary disease | 145 (1.1) | 633 (1.7) | 891 (1.6) | 673 (1.7) | 268 (1.7) | 46 (1.6) |
| Liver disease | 27 (0.2) | 134 (0.4) | 184 (0.3) | 97 (0.2) | 33 (0.2) | 9 (0.3) |
| Rheumatoid arthritis and other inflammatory polyarthropathies | 18 (0.1) | 55 (0.1) | 70 (0.1) | 58 (0.1) | 26 (0.2) | 9 (0.3) |
| History of falls | 303 (2.2) | 1,109 (3.0) | 1,740 (3.1) | 1,306 (3.3) | 521 (3.4) | 93 (3.2) |
| Osteoporosis | 16 (0.1) | 94 (0.3) | 109 (0.2) | 99 (0.3) | 17 (0.1) | 7 (0.2) |
| Hyperparathyroidism | 6 (0.0) | 21 (0.1) | 40 (0.1) | 33 (0.1) | 16 (0.1) | 4 (0.1) |
| Dementia | 9 (0.1) | 86 (0.2) | 132 (0.2) | 91 (0.2) | 37 (0.2) | 12 (0.4) |
| Diabetic retinopathy | 165 (1.2) | 460 (1.2) | 1,181 (2.1) | 777 (2.0) | 296 (1.9) | 71 (2.4) |
| Severe hypoglycemia | 630 (4.6) | 1,663 (4.4) | 2,337 (4.1) | 1,507 (3.9) | 610 (3.9) | 186 (6.4) |
| Use of antidiabetic medications and systemic glucocorticoids, n (%) | |  |  |  |  |  |
| Insulin | 524 (3.8) | 1,126 (3.0) | 1,783 (3.2) | 1,280 (3.3) | 585 (3.8) | 185 (6.4) |
| Metformin | 10,177 (74.1) | 24,817 (66.0) | 35,184 (62.2) | 23,923 (61.3) | 9,839 (63.5) | 1,941 (66.8) |
| Sulfonylurea | 4,528 (33.0) | 10,409 (27.7) | 14,807 (26.2) | 10,591 (27.1) | 4,640 (29.9) | 1,064 (36.6) |
| Thiazolidinedione | 91 (0.7) | 233 (0.6) | 373 (0.7) | 271 (0.7) | 157 (1.0) | 27 (0.9) |
| Dipeptidyl peptidase 4 inhibitors | 240 (1.7) | 495 (1.3) | 776 (1.4) | 648 (1.7) | 320 (2.1) | 103 (3.5) |
| SGLT2 inhibitors | 21 (0.2) | 63 (0.2) | 106 (0.2) | 114 (0.3) | 64 (0.4) | 21 (0.7) |
| Glucagon-like peptide-1 receptor agonist | 0 (0.0) | 6 (0.0) | 21 (0.0) | 25 (0.1) | 8 (0.1) | 10 (0.3) |
| α-Glucosidase inhibitors | 23 (0.2) | 26 (0.1) | 41 (0.1) | 43 (0.1) | 21 (0.1) | 6 (0.2) |
| Systemic glucocorticoids | 288 (2.1) | 1,145 (3.0) | 1,937 (3.4) | 1,451 (3.7) | 568 (3.7) | 125 (4.3) |
| Clinical parameters^†^, median (IQR) |  |  |  |  |  |  |
| eGFR (mL/min/1.73 m^2^) | 95.4 (86.1–103.6) | 90.4 (78.4–100.0) | 88.0 (74.5–98.4) | 86.7 (71.9–98.5) | 86.2 (69.6–99.0) | 84.8 (66.1–100.3) |
| HbA1c (%) | 6.6 (6.2–7.1) | 6.6 (6.2–7.0) | 6.6 (6.2–7.1) | 6.7 (6.3–7.2) | 6.8 (6.3–7.4) | 6.8 (6.4–7.6) |
| HDL-cholesterol (mmol/L) | 1.4 (1.2–1.6) | 1.3 (1.1–1.6) | 1.3 (1.1–1.5) | 1.1 (1.0–1.3) | 1.0 (0.8–1.2) | 0.8 (0.8–0.9) |
| Triglycerides (mmol/L) | 0.9 (0.7–1.2) | 1.1 (0.8–1.4) | 1.3 (1.0–1.6) | 1.8 (1.3–2.3) | 2.3 (1.9–3.0) | 2.7 (2.1–3.7) |
| Urine albumin-to-creatinine ratio (mg/mmol) | 0.8 (0.5–1.3) | 1.0 (0.6–1.6) | 1.2 (0.7–2.4) | 2.4 (1.0–6.7) | 5.1 (2.1–13.9) | 8.6 (4.8–22.4) |

Notes: MetS = Metabolic syndrome; T2D = Type 2 diabetes; IQR = Interquartile range; HDL = High-density lipoprotein; BMI = Body mass index; SGLT2 = Sodium–glucose cotransporter 2; eGFR = estimated glomerular filtration rate; HbA1c = Glycated hemoglobin.

* MetS-related traits included albuminuria and individual MetS-related traits (obesity, hypertension, low HDL-cholesterol, and hypertriglyceridemia).

† Clinical parameters were obtained using the most recent measurement prior to the index date.

## Supplementary Table 3. Incidence rates and HRs for fractures associated with albuminuria and MetS traits present at baseline in patients with T2D.

| Outcome | Event | Incidence rate (per 1,000 person-years) | Crude HR | 95% CI | P-value | Adjusted HR^*^ | 95% CI | P-value |
| --- | --- | --- | --- | --- | --- | --- | --- | --- |
| **Hip fracture** |  |  |  |  |  |  |  |  |
| ***Individual MetS-related traits***^†^ | |  |  |  |  |  |  |  |
| No albuminuria | 882 | 1.30 | Reference | | | Reference | | |
| Albuminuria | 701 | 2.90 | 2.037 | (1.841, 2.254) | <0.001 | 1.455 | (1.301, 1.628) | <0.001 |
| No hypertension | 164 | 0.79 | Reference | | | Reference | | |
| Hypertension | 1,419 | 1.99 | 2.540 | (2.153, 2.995) | <0.001 | 1.324 | (1.099, 1.593) | 0.003 |
| No hypertriglyceridemia | 1,108 | 1.81 | Reference | | | Reference | | |
| Hypertriglyceridemia | 475 | 1.54 | 0.894 | (0.800, 0.999) | 0.049 | 0.959 | (0.850, 1.082) | 0.496 |
| No low HDL-C level | 1,413 | 1.75 | Reference | | | Reference | | |
| Low HDL-C level | 170 | 1.50 | 0.907 | (0.770, 1.068) | 0.242 | 0.923 | (0.774, 1.100) | 0.369 |
| No obesity | 966 | 2.53 | Reference | | | Reference | | |
| Obesity | 617 | 1.15 | 0.411 | (0.371, 0.455) | <0.001 | 0.508 | (0.455, 0.568) | <0.001 |
| **Major osteoporotic fracture** |  |  |  |  |  |  |  |  |
| ***Individual MetS-related traits***^†^ | |  |  |  |  |  |  |  |
| No albuminuria | 2,160 | 3.21 | Reference | | | Reference | | |
| Albuminuria | 1,233 | 5.14 | 1.507 | (1.403, 1.618) | <0.001 | 1.208 | (1.117, 1.307) | <0.001 |
| No hypertension | 480 | 2.33 | Reference | | | Reference | | |
| Hypertension | 2,913 | 4.12 | 1.799 | (1.630, 1.986) | <0.001 | 1.142 | (1.024, 1.274) | 0.017 |
| No hypertriglyceridemia | 2,329 | 3.84 | Reference | | | Reference | | |
| Hypertriglyceridemia | 1,064 | 3.47 | 0.938 | (0.870, 1.012) | 0.097 | 0.970 | (0.895, 1.052) | 0.465 |
| No low HDL-C level | 3,026 | 3.78 | Reference | | | Reference | | |
| Low HDL-C level | 367 | 3.27 | 0.893 | (0.799, 0.998) | 0.047 | 0.914 | (0.811, 1.029) | 0.137 |
| No obesity | 1,789 | 4.72 | Reference | | | Reference | | |
| Obesity | 1,604 | 3.00 | 0.595 | (0.555, 0.637) | <0.001 | 0.711 | (0.661, 0.765) | <0.001 |

Notes: HR = Hazard ratio; CI = Confidence interval; MetS = Metabolic syndrome; T2D = Type 2 diabetes; HbA1c = Glycated hemoglobin; HDL = High-density lipoprotein.

* The models were adjusted by age, sex, smoking status, cardiovascular disease, hyperlipidemia, chronic kidney disease, chronic obstructive pulmonary disease, liver disease, rheumatoid arthritis and other inflammatory polyarthropathies, history of falls (as a proxy indicator for frailty), osteoporosis, hyperparathyroidism, dementia, diabetic retinopathy, severe hypoglycemia, use of antidiabetic medications and systemic glucocorticoids, and baseline HbA1c level.

† MetS-related traits include albuminuria and individual MetS-related traits (obesity, hypertension, low HDL-cholesterol, and hypertriglyceridemia).

## Supplementary Table 4. Incidence rates and HRs for fractures associated with albuminuria and MetS traits present at baseline in obese and non-obese older adults with T2D.

| Outcome | **Obese (N = 77,137)** | | | | | **Non-obese (N = 60,976)** | | | | |
| --- | --- | --- | --- | --- | --- | --- | --- | --- | --- | --- |
|  | Event | Incidence rate (per 1,000 person-years) | Adjusted HR^*^ | 95% CI | P-value | Event | Incidence rate (per 1,000 person-years) | Adjusted HR^*^ | 95% CI | P-value |
| **Hip fracture** |  |  |  |  |  |  |  |  |  |  |
| ***Number of MetS-related traits***^†^ |  |  |  |  |  |  |  |  |  |  |
| 1 (T2D only) | NA | | | | | 60 | 1.00 | Reference | | |
| 2 | 24 | 0.79 | Reference | | | 367 | 2.53 | 1.811 | (1.339, 2.449) | <0.001 |
| 3 | 196 | 1.15 | 1.109 | (0.678, 1.811) | 0.681 | 378 | 4.03 | 2.122 | (1.561, 2.883) | <0.001 |
| 4 | 256 | 1.71 | 1.394 | (0.855, 2.273) | 0.183 | 127 | 4.45 | 2.185 | (1.550, 3.080) | <0.001 |
| 5 | 103 | 1.65 | 1.164 | (0.690, 1.961) | 0.569 | 23 | 4.70 | 1.892 | (1.126, 3.179) | 0.016 |
| 6 | 28 | 2.52 | 1.655 | (0.878, 3.118) | 0.119 | NA | | | | |
| ≥3 or ≥2^‡^ | 583 | 1.48 | 1.238 | (0.768, 1.997) | 0.381 | 895 | 3.29 | 1.957 | (1.460, 2.623) | <0.001 |
| Continuous | NA | | 1.090 | (0.987, 1.203) | 0.089 | NA | | 1.165 | (1.080, 1.257) | <0.001 |
| ***Individual MetS-related traits***^†^ |  |  |  |  |  |  |  |  |  |  |
| No albuminuria | 349 | 1.15 | Reference | | | 524 | 2.08 | Reference | | |
| Albuminuria | 258 | 2.15 | 1.294 | (1.077, 1.554) | 0.006 | 431 | 5.38 | 1.533 | (1.327, 1.771) | <0.001 |
| No hypertension | 42 | 0.75 | Reference | | | 115 | 1.33 | Reference | | |
| Hypertension | 565 | 1.54 | 1.198 | (0.835, 1.718) | 0.328 | 840 | 3.42 | 1.371 | (1.099, 1.711) | 0.005 |
| No hypertriglyceridemia | 386 | 1.46 | Reference | | | 710 | 2.86 | Reference | | |
| Hypertriglyceridemia | 221 | 1.38 | 0.895 | (0.741, 1.082) | 0.254 | 245 | 2.94 | 1.007 | (0.859, 1.181) | 0.930 |
| No low HDL-C level | 522 | 1.41 | Reference | | | 875 | 2.89 | Reference | | |
| Low HDL-C level | 85 | 1.58 | 1.123 | (0.868, 1.453) | 0.376 | 80 | 2.77 | 0.776 | (0.606, 0.995) | 0.045 |
|  |  |  |  |  |  |  |  |  |  |  |
| **Major osteoporotic fracture** |  |  |  |  |  |  |  |  |  |  |
| ***Number of MetS-related traits***^†^ |  |  |  |  |  |  |  |  |  |  |
| 1 (T2D only) | NA | | | | | 161 | 2.72 | Reference | | |
| 2 | 92 | 3.05 | Reference | | | 705 | 4.90 | 1.424 | (1.183, 1.713) | <0.001 |
| 3 | 553 | 3.27 | 0.871 | (0.688, 1.103) | 0.253 | 625 | 6.72 | 1.556 | (1.285, 1.884) | <0.001 |
| 4 | 580 | 3.91 | 0.937 | (0.738, 1.188) | 0.591 | 197 | 6.97 | 1.424 | (1.130, 1.794) | 0.003 |
| 5 | 231 | 3.72 | 0.846 | (0.650, 1.101) | 0.214 | 40 | 8.29 | 1.445 | (0.992, 2.107) | 0.055 |
| 6 | 53 | 4.81 | 0.982 | (0.675, 1.429) | 0.925 | NA | | | | |
| ≥3 or ≥2^‡^ | 1,417 | 3.63 | 0.895 | (0.712, 1.123) | 0.338 | 1,567 | 5.80 | 1.468 | (1.229, 1.754) | <0.001 |
| Continuous | NA | | 0.997 | (0.937, 1.060) | 0.913 | NA | | 1.086 | (1.027, 1.149) | 0.004 |
| ***Individual MetS-related traits***^†^ |  |  |  |  |  |  |  |  |  |  |
| No albuminuria | 986 | 3.27 | Reference | | | 1,075 | 4.30 | Reference | | |
| Albuminuria | 523 | 4.39 | 1.104 | (0.982, 1.242) | 0.098 | 653 | 8.23 | 1.275 | (1.141, 1.424) | <0.001 |
| No hypertension | 155 | 2.79 | Reference | | | 269 | 3.13 | Reference | | |
| Hypertension | 1,354 | 3.71 | 0.944 | (0.787, 1.133) | 0.536 | 1,459 | 5.99 | 1.232 | (1.064, 1.426) | 0.005 |
| No hypertriglyceridemia | 961 | 3.67 | Reference | | | 1,278 | 5.18 | Reference | | |
| Hypertriglyceridemia | 548 | 3.45 | 0.916 | (0.816, 1.029) | 0.140 | 450 | 5.45 | 1.007 | (0.895, 1.133) | 0.905 |
| No low HDL-C level | 1,316 | 3.58 | Reference | | | 1,584 | 5.27 | Reference | | |
| Low HDL-C level | 193 | 3.61 | 1.028 | (0.871, 1.214) | 0.743 | 144 | 5.03 | 0.787 | (0.653, 0.950) | 0.012 |

Notes: HR = Hazard ratio; CI = Confidence interval; MetS = Metabolic syndrome; T2D = Type 2 diabetes; HbA1c = Glycated hemoglobin; HDL = High-density lipoprotein; NA = Not available.

* The models were adjusted by age, sex, smoking status, cardiovascular disease, hyperlipidemia, chronic kidney disease, chronic obstructive pulmonary disease, liver disease, rheumatoid arthritis and other inflammatory polyarthropathies, history of falls (as a proxy indicator for frailty), osteoporosis, hyperparathyroidism, dementia, diabetic retinopathy, severe hypoglycemia, use of antidiabetic medications and systemic glucocorticoids, and baseline HbA1c level.

† MetS-related traits include albuminuria and individual MetS-related traits (obesity, hypertension, low HDL-cholesterol, and hypertriglyceridemia).

‡ ≥2 MetS-related traits indicate the presence of at least one additional MetS-related trait beyond T2D, while ≥3 MetS-related traits indicate at least one additional MetS-related trait beyond T2D and obesity.

## Supplementary Table 5. Incidence rates and HRs for fractures associated with albuminuria and MetS traits present at baseline in obese and non-obese young adults with T2D.

| Outcome | **Obese (N = 19,114)** | | | | | **Non-obese (N = 8,062)** | | | | |
| --- | --- | --- | --- | --- | --- | --- | --- | --- | --- | --- |
|  | Event | Incidence rate (per 1,000 person-years) | Adjusted HR^*^ | 95% CI | P-value | Event | Incidence rate (per 1,000 person-years) | Adjusted HR^*^ | 95% CI | P-value |
| **Fracture** |  |  |  |  |  |  |  |  |  |  |
| ***Number of MetS-related traits***^†^ | |  |  |  |  |  |  |  |  |  |
| 1 (T2D only) | NA | | | | | 19 | 1.03 | Reference | | |
| 2 | 8 | 0.48 | Reference | | | 13 | 0.78 | 0.706 | (0.330, 1.513) | 0.371 |
| 3 | 23 | 0.62 | 1.235 | (0.547, 2.788) | 0.611 | 24 | 2.25 | 2.327 | (1.224, 4.425) | 0.010 |
| 4 | 42 | 1.19 | 2.058 | (0.946, 4.476) | 0.069 | 4 | 1.18 | 1.232 | (0.407, 3.730) | 0.712 |
| 5 | 19 | 0.99 | 1.816 | (0.775, 4.255) | 0.170 | 1 | 1.35 | 1.363 | (0.178, 10.451) | 0.766 |
| 6 | 3 | 0.64 | 0.751 | (0.157, 3.595) | 0.720 | NA | | | | |
| ≥3 or ≥2^‡^ | 87 | 0.90 | 1.616 | (0.772, 3.383) | 0.203 | 42 | 1.33 | 1.294 | (0.723, 2.319) | 0.385 |
| Continuous | NA | | 1.133 | (0.926, 1.388) | 0.225 | NA | | 1.277 | (0.992, 1.643) | 0.058 |
| ***Individual MetS-related traits***^†^ | |  |  |  |  |  |  |  |  |  |
| No albuminuria | 54 | 0.67 | Reference | | | 45 | 1.12 | Reference | | |
| Albuminuria | 41 | 1.29 | 1.600 | (1.006, 2.545) | 0.047 | 16 | 1.65 | 1.451 | (0.757, 2.780) | 0.262 |
| No hypertension | 26 | 0.72 | Reference | | | 30 | 1.06 | Reference | | |
| Hypertension | 69 | 0.90 | 1.015 | (0.609, 1.691) | 0.956 | 31 | 1.42 | 1.287 | (0.722, 2.295) | 0.393 |
| No hypertriglyceridemia | 48 | 0.78 | Reference | | | 42 | 1.15 | Reference | | |
| Hypertriglyceridemia | 47 | 0.91 | 1.077 | (0.693, 1.672) | 0.742 | 19 | 1.42 | 1.094 | (0.608, 1.970) | 0.765 |
| No low HDL-C level | 76 | 0.85 | Reference | | | 50 | 1.14 | Reference | | |
| Low HDL-C level | 19 | 0.79 | 0.886 | (0.517, 1.519) | 0.661 | 11 | 1.73 | 1.358 | (0.665, 2.777) | 0.401 |

Notes: HR = Hazard ratio; CI = Confidence interval; MetS = Metabolic syndrome; T2D = Type 2 diabetes; HbA1c = Glycated hemoglobin; HDL = High-density lipoprotein; NA = Not available. Results for hip fracture were omitted because the number of events for each category of MetS traits did not exceed 10.

* The models were adjusted by age, sex, smoking status, cardiovascular disease, hyperlipidemia, chronic kidney disease, chronic obstructive pulmonary disease, liver disease, rheumatoid arthritis and other inflammatory polyarthropathies, history of falls (as a proxy indicator for frailty), osteoporosis, hyperparathyroidism, dementia, diabetic retinopathy, severe hypoglycemia, use of antidiabetic medications and systemic glucocorticoids, and baseline HbA1c level.

† MetS-related traits include albuminuria and individual MetS-related traits (obesity, hypertension, low HDL-cholesterol, and hypertriglyceridemia).

‡ ≥2 MetS-related traits indicate the presence of at least one additional MetS-related trait beyond T2D, while ≥3 MetS-related traits indicate at least one additional MetS-related trait beyond T2D and obesity.

## Supplementary Table 6. Incidence rates and HRs for fractures associated with albuminuria and MetS traits present at baseline in obese and non-obese male patients with T2D.

| Outcome | **Obese (N = 52,148)** | | | | | **Non-obese (N = 37,480)** | | | | |
| --- | --- | --- | --- | --- | --- | --- | --- | --- | --- | --- |
|  | Event | Incidence rate (per 1,000 person-years) | Adjusted HR^*^ | 95% CI | P-value | Event | Incidence rate (per 1,000 person-years) | Adjusted HR^*^ | 95% CI | P-value |
| **Hip fracture** |  |  |  |  |  |  |  |  |  |  |
| ***Number of MetS-related traits***^†^ | |  |  |  |  |  |  |  |  |  |
| 1 (T2D only) | NA | | | | | 23 | 0.54 | Reference | | |
| 2 | 6 | 0.24 | Reference | | | 153 | 1.75 | 2.254 | (1.369, 3.713) | 0.001 |
| 3 | 63 | 0.57 | 1.622 | (0.645, 4.078) | 0.304 | 141 | 2.53 | 2.661 | (1.603, 4.420) | <0.001 |
| 4 | 93 | 0.94 | 2.308 | (0.925, 5.763) | 0.073 | 47 | 2.95 | 3.227 | (1.838, 5.666) | <0.001 |
| 5 | 44 | 0.99 | 2.262 | (0.875, 5.851) | 0.092 | 11 | 4.10 | 4.345 | (1.981, 9.530) | <0.001 |
| 6 | 10 | 1.09 | 2.622 | (0.844, 8.150) | 0.096 | NA | | | | |
| ≥3 or ≥2^‡^ | 210 | 0.80 | 2.009 | (0.818, 4.938) | 0.128 | 352 | 2.17 | 2.520 | (1.551, 4.095) | <0.001 |
| Continuous | NA | | 1.216 | (1.038, 1.425) | 0.015 | NA |  | 1.315 | (1.165, 1.484) | <0.001 |
| ***Individual MetS-related traits***^†^ | |  |  |  |  |  |  |  |  |  |
| No albuminuria | 109 | 0.52 | Reference | | | 205 | 1.30 | Reference | | |
| Albuminuria | 107 | 1.34 | 1.825 | (1.351, 2.467) | <0.001 | 170 | 3.62 | 1.686 | (1.340, 2.121) | <0.001 |
| No hypertension | 15 | 0.29 | Reference | | | 46 | 0.74 | Reference | | |
| Hypertension | 201 | 0.85 | 1.281 | (0.725, 2.264) | 0.394 | 329 | 2.32 | 1.617 | (1.133, 2.305) | 0.008 |
| No hypertriglyceridemia | 138 | 0.80 | Reference | | | 291 | 1.88 | Reference | | |
| Hypertriglyceridemia | 78 | 0.67 | 0.915 | (0.669, 1.251) | 0.578 | 84 | 1.71 | 1.086 | (0.835, 1.414) | 0.538 |
| No low HDL-C level | 181 | 0.75 | Reference | | | 338 | 1.83 | Reference | | |
| Low HDL-C level | 35 | 0.76 | 1.034 | (0.691, 1.547) | 0.871 | 37 | 1.91 | 0.951 | (0.660, 1.371) | 0.788 |
|  |  |  |  |  |  |  |  |  |  |  |
| **Major osteoporotic fracture** |  |  |  |  |  |  |  |  |  |  |
| ***Number of MetS-related traits***^†^ | |  |  |  |  |  |  |  |  |  |
| 1 (T2D only) | NA | | | | | 68 | 1.61 | Reference | | |
| 2 | 28 | 1.10 | Reference | | | 272 | 3.12 | 1.442 | (1.080, 1.926) | 0.013 |
| 3 | 178 | 1.62 | 1.141 | (0.756, 1.722) | 0.531 | 221 | 3.98 | 1.583 | (1.172, 2.139) | 0.003 |
| 4 | 224 | 2.27 | 1.473 | (0.979, 2.218) | 0.063 | 76 | 4.80 | 1.801 | (1.253, 2.590) | 0.001 |
| 5 | 86 | 1.93 | 1.181 | (0.754, 1.849) | 0.468 | 15 | 5.61 | 2.101 | (1.143, 3.862) | 0.017 |
| 6 | 18 | 1.97 | 1.138 | (0.600, 2.158) | 0.693 | NA | | | | |
| ≥3 or ≥2^‡^ | 506 | 1.93 | 1.269 | (0.855, 1.884) | 0.237 | 584 | 3.62 | 1.534 | (1.163, 2.023) | 0.002 |
| Continuous | NA | | 1.054 | (0.957, 1.162) | 0.285 | NA | | 1.175 | (1.071, 1.288) | <0.001 |
| ***Individual MetS-related traits***^†^ | |  |  |  |  |  |  |  |  |  |
| No albuminuria | 335 | 1.61 | Reference | | | 404 | 2.58 | Reference | | |
| Albuminuria | 199 | 2.51 | 1.255 | (1.033, 1.524) | 0.022 | 248 | 5.30 | 1.394 | (1.163, 1.671) | <0.001 |
| No hypertension | 60 | 1.16 | Reference | | | 119 | 1.92 | Reference | | |
| Hypertension | 474 | 2.01 | 1.208 | (0.901, 1.618) | 0.206 | 533 | 3.77 | 1.218 | (0.968, 1.532) | 0.093 |
| No hypertriglyceridemia | 328 | 1.91 | Reference | | | 494 | 3.20 | Reference | | |
| Hypertriglyceridemia | 206 | 1.78 | 0.968 | (0.800, 1.171) | 0.737 | 158 | 3.23 | 1.106 | (0.908, 1.347) | 0.318 |
| No low HDL-C level | 457 | 1.89 | Reference | | | 589 | 3.20 | Reference | | |
| Low HDL-C level | 77 | 1.68 | 0.856 | (0.659, 1.112) | 0.244 | 63 | 3.26 | 0.909 | (0.684, 1.208) | 0.510 |

Notes: HR = Hazard ratio; CI = Confidence interval; MetS = Metabolic syndrome; T2D = Type 2 diabetes; HbA1c = Glycated hemoglobin; HDL = High-density lipoprotein; NA = Not available.

* The models were adjusted by age, sex, smoking status, cardiovascular disease, hyperlipidemia, chronic kidney disease, chronic obstructive pulmonary disease, liver disease, rheumatoid arthritis and other inflammatory polyarthropathies, history of falls (as a proxy indicator for frailty), osteoporosis, hyperparathyroidism, dementia, diabetic retinopathy, severe hypoglycemia, use of antidiabetic medications and systemic glucocorticoids, and baseline HbA1c level.

† MetS-related traits include albuminuria and individual MetS traits (obesity, hypertension, low HDL-cholesterol, and hypertriglyceridemia).

‡ ≥2 MetS-related traits indicate the presence of at least one additional MetS-related trait beyond T2D, while ≥3 MetS-related traits indicate at least one additional MetS-related trait beyond T2D and obesity.

## Supplementary Table 7. Incidence rates and HRs for fractures associated with albuminuria and MetS traits present at baseline in obese and non-obese female patients with T2D.

| Outcome | **Obese (N = 44,103)** | | | | | **Non-obese (N = 31,558)** | | | | |
| --- | --- | --- | --- | --- | --- | --- | --- | --- | --- | --- |
|  | Event | Incidence rate (per 1,000 person-years) | Adjusted HR^*^ | 95% CI | P-value | Event | Incidence rate (per 1,000 person-years) | Adjusted HR^*^ | 95% CI | P-value |
| **Hip fracture** |  |  |  |  |  |  |  |  |  |  |
| ***Number of MetS-related traits***^†^ |  |  |  |  |  |  |  |  |  |  |
| 1 (T2D only) | NA | | | | | 40 | 1.12 | Reference | | |
| 2 | 18 | 0.84 | Reference | | | 215 | 2.90 | 1.522 | (1.050, 2.206) | 0.027 |
| 3 | 133 | 1.37 | 0.970 | (0.542, 1.737) | 0.920 | 243 | 4.98 | 1.834 | (1.259, 2.673) | 0.002 |
| 4 | 169 | 1.97 | 1.186 | (0.664, 2.120) | 0.564 | 81 | 5.06 | 1.710 | (1.117, 2.616) | 0.013 |
| 5 | 63 | 1.69 | 0.912 | (0.486, 1.709) | 0.773 | 12 | 4.06 | 1.150 | (0.575, 2.299) | 0.693 |
| 6 | 18 | 2.71 | 1.333 | (0.616, 2.884) | 0.465 | NA | | | | |
| ≥3 or ≥2^‡^ | 383 | 1.69 | 1.050 | (0.596, 1.850) | 0.865 | 551 | 3.88 | 1.641 | (1.146, 2.349) | 0.007 |
| Continuous | NA | | 1.040 | (0.918, 1.177) | 0.541 | NA | | 1.097 | (0.996, 1.207) | 0.059 |
| ***Individual MetS-related traits***^†^ |  |  |  |  |  |  |  |  |  |  |
| No albuminuria | 242 | 1.37 | Reference | | | 326 | 2.42 | Reference | | |
| Albuminuria | 159 | 2.21 | 1.109 | (0.882, 1.394) | 0.377 | 265 | 6.18 | 1.464 | (1.218, 1.760) | <0.001 |
| No hypertension | 28 | 0.70 | Reference | | | 75 | 1.43 | Reference | | |
| Hypertension | 373 | 1.79 | 1.217 | (0.769, 1.926) | 0.402 | 516 | 4.12 | 1.200 | (0.910, 1.581) | 0.196 |
| No hypertriglyceridemia | 253 | 1.65 | Reference | | | 426 | 3.27 | Reference | | |
| Hypertriglyceridemia | 148 | 1.55 | 0.882 | (0.697, 1.116) | 0.296 | 165 | 3.47 | 0.974 | (0.799, 1.187) | 0.793 |
| No low HDL-C level | 349 | 1.61 | Reference | | | 545 | 3.37 | Reference | | |
| Low HDL-C level | 52 | 1.63 | 1.175 | (0.845, 1.632) | 0.338 | 46 | 2.90 | 0.726 | (0.524, 1.007) | 0.055 |
|  |  |  |  |  |  |  |  |  |  |  |
| **Major osteoporotic fracture** |  |  |  |  |  |  |  |  |  |  |
| ***Number of MetS-related traits***^†^ |  |  |  |  |  |  |  |  |  |  |
| 1 (T2D only) | NA | | | | | 112 | 3.15 | Reference | | |
| 2 | 72 | 3.39 | Reference | | | 446 | 6.08 | 1.341 | (1.071, 1.677) | 0.010 |
| 3 | 398 | 4.13 | 0.806 | (0.614, 1.058) | 0.120 | 428 | 8.89 | 1.545 | (1.226, 1.947) | <0.001 |
| 4 | 398 | 4.67 | 0.815 | (0.619, 1.073) | 0.145 | 125 | 7.89 | 1.232 | (0.928, 1.637) | 0.149 |
| 5 | 164 | 4.44 | 0.789 | (0.582, 1.070) | 0.127 | 26 | 8.98 | 1.220 | (0.768, 1.939) | 0.399 |
| 6 | 38 | 5.78 | 0.896 | (0.576, 1.395) | 0.627 | NA | | | | |
| ≥3 or ≥2^‡^ | 998 | 4.44 | 0.809 | (0.623, 1.051) | 0.112 | 1,025 | 7.31 | 1.395 | (1.125, 1.729) | 0.002 |
| Continuous | NA | | 0.978 | (0.909, 1.053) | 0.562 | NA | | 1.058 | (0.988, 1.134) | 0.105 |
| ***Individual MetS-related traits***^†^ |  |  |  |  |  |  |  |  |  |  |
| No albuminuria | 705 | 4.03 | Reference | | | 716 | 5.36 | Reference | | |
| Albuminuria | 365 | 5.12 | 1.060 | (0.921, 1.220) | 0.415 | 421 | 9.95 | 1.234 | (1.077, 1.415) | 0.002 |
| No hypertension | 121 | 3.04 | Reference | | | 180 | 3.46 | Reference | | |
| Hypertension | 949 | 4.60 | 0.839 | (0.679, 1.036) | 0.103 | 957 | 7.73 | 1.243 | (1.038, 1.488) | 0.018 |
| No hypertriglyceridemia | 681 | 4.49 | Reference | | | 826 | 6.41 | Reference | | |
| Hypertriglyceridemia | 389 | 4.12 | 0.906 | (0.788, 1.041) | 0.163 | 311 | 6.62 | 0.969 | (0.840, 1.118) | 0.669 |
| No low HDL-C level | 935 | 4.36 | Reference | | | 1,045 | 6.52 | Reference | | |
| Low HDL-C level | 135 | 4.28 | 1.119 | (0.916, 1.366) | 0.272 | 92 | 5.88 | 0.766 | (0.606, 0.969) | 0.026 |

Notes: HR = Hazard ratio; CI = Confidence interval; MetS = Metabolic syndrome; T2D = Type 2 diabetes; HbA1c = Glycated hemoglobin; HDL = High-density lipoprotein; NA = Not available.

* The models were adjusted by age, sex, smoking status, cardiovascular disease, hyperlipidemia, chronic kidney disease, chronic obstructive pulmonary disease, liver disease, rheumatoid arthritis and other inflammatory polyarthropathies, history of falls (as a proxy indicator for frailty), osteoporosis, hyperparathyroidism, dementia, diabetic retinopathy, severe hypoglycemia, use of antidiabetic medications and systemic glucocorticoids, and baseline HbA1c level.

† MetS-related traits include albuminuria and individual MetS traits (obesity, hypertension, low HDL-cholesterol, and hypertriglyceridemia).

‡ ≥2 MetS-related traits indicate the presence of at least one additional MetS-related trait beyond T2D, while ≥3 MetS-related traits indicate at least one additional MetS-related trait beyond T2D and obesity.

## Supplementary Table 8. Incidence rates and HRs for fractures associated with albuminuria and MetS traits present at baseline in obese and non-obese patients with T2D, stratified by osteoporosis status.

| *Subgroup: patients with osteoporosis* | | | | | | | | |
| --- | --- | --- | --- | --- | --- | --- | --- | --- |
|  | **Obese (N =145)** | | **Non-obese (N =197)** | | **Obese (N =145)** | | **Non-obese (N =197)** | |
|  | Event | Incidence rate (per 1,000 person-years) | Event | Incidence rate (per 1,000 person-years) | Event | Incidence rate (per 1,000 person-years) | Event | Incidence rate (per 1,000 person-years) |
| ***Number of MetS-related traits***^†^ | **Hip fracture** | | | | **Major osteoporotic fracture** | | | |
| 1 (T2D only) | NA | | 2 | 34.53 | NA | | 2 | 34.53 |
| 2 | 0 | 0.00 | 3 | 9.47 | 0 | 0.00 | 4 | 12.78 |
| 3 | 4 | 20.99 | 1 | 3.87 | 4 | 20.99 | 6 | 23.77 |
| 4 | 3 | 12.66 | 0 | 0.00 | 4 | 16.88 | 1 | 9.93 |
| 5 | 0 | 0.00 | 0 | 0.00 | 1 | 20.60 | 0 | 0.00 |
| 6 | 0 | 0.00 | NA | | 0 | 0.00 | NA | |
| ≥3 or ≥2^‡^ | 7 | 13.78 | 4 | 5.81 | 9 | 17.76 | 11 | 16.28 |
| Continuous | NA | | NA | | NA | | NA | |
| ***Individual MetS-related traits***^†^ |  | | | |  | | | |
| No albuminuria | 5 | 14.90 | 5 | 9.81 | 6 | 17.89 | 7 | 13.92 |
| Albuminuria | 2 | 11.36 | 1 | 4.22 | 3 | 17.15 | 6 | 26.03 |
| No hypertension | 0 | 0.00 | 3 | 39.05 | 0 | 0.00 | 3 | 39.05 |
| Hypertension | 7 | 14.68 | 3 | 4.48 | 9 | 18.92 | 10 | 15.23 |
| No hypertriglyceridemia | 6 | 19.86 | 5 | 9.06 | 7 | 23.25 | 11 | 20.30 |
| Hypertriglyceridemia | 1 | 4.78 | 1 | 5.13 | 2 | 9.56 | 2 | 10.43 |
| No low HDL-C level | 7 | 16.02 | 6 | 9.04 | 8 | 18.31 | 12 | 18.33 |
| Low HDL-C level | 0 | 0.00 | 0 | 0.00 | 1 | 13.63 | 1 | 12.69 |

| *Subgroup: patients without osteoporosis* | | | | | | | | | | | |
| --- | --- | --- | --- | --- | --- | --- | --- | --- | --- | --- | --- |
| Outcome | **Obese (N =96,106)** | | | | | **Non-obese (N =68,841)** | | | | | |
|  | Event | Incidence rate (per 1,000 person-years) | Adjusted HR^*^ | 95% CI | P-value | Event | Incidence rate (per 1,000 person-years) | Adjusted HR^*^ | 95% CI | P-value |  |
| **Hip fracture** |  |  |  |  |  |  |  |  |  |  |  |
| ***Number of MetS-related traits***^†^ |  |  |  |  |  |  |  |  |  |  |  |
| 1 (T2D only) | NA | | | | | 61 | 0.78 | Reference | | | |
| 2 | 24 | 0.51 | Reference | | | 365 | 2.26 | 1.858 | (1.373, 2.514) | <0.001 |  |
| 3 | 192 | 0.93 | 1.154 | (0.706, 1.887) | 0.567 | 383 | 3.67 | 2.232 | (1.642, 3.033) | <0.001 |  |
| 4 | 259 | 1.40 | 1.521 | (0.933, 2.479) | 0.093 | 128 | 4.02 | 2.278 | (1.616, 3.211) | <0.001 |  |
| 5 | 107 | 1.31 | 1.296 | (0.771, 2.179) | 0.328 | 23 | 4.09 | 1.946 | (1.158, 3.270) | 0.012 |  |
| 6 | 28 | 1.77 | 1.758 | (0.933, 3.312) | 0.081 | NA | | | | | |
| ≥3 or ≥2^‡^ | 586 | 1.20 | 1.330 | (0.825, 2.145) | 0.242 | 899 | 2.97 | 2.030 | (1.514, 2.722) | <0.001 |  |
| Continuous | NA | | 1.117 | (1.013, 1.231) | 0.027 | NA | | 1.178 | (1.093, 1.269) | <0.001 |  |
| ***Individual MetS-related traits***^†^ |  |  |  |  |  |  |  |  |  |  |  |
| No albuminuria | 346 | 0.90 | Reference | | | 526 | 1.80 | Reference | | | |
| Albuminuria | 264 | 1.74 | 1.353 | (1.127, 1.624) | 0.001 | 434 | 4.84 | 1.548 | (1.340, 1.787) | <0.001 |  |
| No hypertension | 43 | 0.47 | Reference | | | 118 | 1.03 | Reference | | | |
| Hypertension | 567 | 1.27 | 1.241 | (0.868, 1.775) | 0.236 | 842 | 3.16 | 1.393 | (1.117, 1.736) | 0.003 |  |
| No hypertriglyceridemia | 385 | 1.18 | Reference | | | 712 | 2.50 | Reference | | | |
| Hypertriglyceridemia | 225 | 1.06 | 0.909 | (0.753, 1.097) | 0.319 | 248 | 2.57 | 1.010 | (0.862, 1.184) | 0.901 |  |
| No low HDL-C level | 523 | 1.14 | Reference | | | 877 | 2.53 | Reference | | | |
| Low HDL-C level | 87 | 1.12 | 1.128 | (0.874, 1.455) | 0.356 | 83 | 2.36 | 0.801 | (0.628, 1.022) | 0.074 |  |
|  |  |  |  |  |  |  |  |  |  |  |  |
| **Major osteoporotic fracture** |  |  |  |  |  |  |  |  |  |  |  |
| ***Number of MetS-related traits***^†^ |  |  |  |  |  |  |  |  |  |  |  |
| 1 (T2D only) | NA | | | | | 175 | 2.25 | Reference | | | |
| 2 | 99 | 2.12 | Reference | | | 709 | 4.42 | 1.423 | (1.189, 1.703) | <0.001 |  |
| 3 | 562 | 2.73 | 0.919 | (0.731, 1.155) | 0.470 | 639 | 6.18 | 1.605 | (1.334, 1.933) | <0.001 |  |
| 4 | 606 | 3.30 | 1.024 | (0.814, 1.288) | 0.837 | 199 | 6.30 | 1.451 | (1.158, 1.820) | 0.001 |  |
| 5 | 246 | 3.03 | 0.930 | (0.722, 1.198) | 0.574 | 39 | 7.00 | 1.449 | (0.997, 2.106) | 0.052 |  |
| 6 | 55 | 3.51 | 0.999 | (0.692, 1.442) | 0.996 | NA | | | | | |
| ≥3 or ≥2^‡^ | 1,469 | 3.02 | 0.962 | (0.773, 1.198) | 0.729 | 1,586 | 5.27 | 1.487 | (1.252, 1.766) | <0.001 |  |
| Continuous | NA | | 1.013 | (0.955, 1.075) | 0.661 | NA | | 1.099 | (1.040, 1.162) | <0.001 |  |
| ***Individual MetS-related traits***^†^ |  |  |  |  |  |  |  |  |  |  |  |
| No albuminuria | 1,015 | 2.65 | Reference | | | 1,104 | 3.81 | Reference | | | |
| Albuminuria | 553 | 3.67 | 1.138 | (1.015, 1.277) | 0.027 | 657 | 7.39 | 1.276 | (1.143, 1.424) | <0.001 |  |
| No hypertension | 180 | 1.97 | Reference | | | 293 | 2.57 | Reference | | | |
| Hypertension | 1,388 | 3.14 | 0.963 | (0.810, 1.144) | 0.666 | 1,468 | 5.55 | 1.256 | (1.089, 1.449) | 0.002 |  |
| No hypertriglyceridemia | 986 | 3.05 | Reference | | | 1,298 | 4.59 | Reference | | | |
| Hypertriglyceridemia | 582 | 2.77 | 0.935 | (0.835, 1.047) | 0.242 | 463 | 4.83 | 1.020 | (0.908, 1.146) | 0.742 |  |
| No low HDL-C level | 1,359 | 2.98 | Reference | | | 1,609 | 4.68 | Reference | | | |
| Low HDL-C level | 209 | 2.70 | 1.016 | (0.866, 1.192) | 0.842 | 152 | 4.35 | 0.810 | (0.675, 0.972) | 0.023 |  |

Notes: HR = Hazard ratio; CI = Confidence interval; MetS = Metabolic syndrome; T2D = Type 2 diabetes; HbA1c = Glycated hemoglobin; HDL = High-density lipoprotein; NA = Not available. HRs for patients with osteoporosis were omitted because the number of events in most MetS trait categories did not exceed 10.

* The models were adjusted by age, sex, smoking status, cardiovascular disease, hyperlipidemia, chronic kidney disease, chronic obstructive pulmonary disease, liver disease, rheumatoid arthritis and other inflammatory polyarthropathies, history of falls (as a proxy indicator for frailty), hyperparathyroidism, dementia, diabetic retinopathy, severe hypoglycemia, use of antidiabetic medications and systemic glucocorticoids, and baseline HbA1c level.

† MetS-related traits include albuminuria and individual MetS traits (obesity, hypertension, low HDL-cholesterol, and hypertriglyceridemia).

‡ ≥2 MetS-related traits indicate the presence of at least one additional MetS-related trait beyond T2D, while ≥3 MetS-related traits indicate at least one additional MetS-related trait beyond T2D and obesity.

## Supplementary Table 9. Incidence rates and HRs for fractures associated with individual MetS-related traits by time since T2D diagnosis.

| Outcome | Time since T2D diagnosis ≥ 1 year | | | | | Time since T2D diagnosis ≥ 5 years | | | | |
| --- | --- | --- | --- | --- | --- | --- | --- | --- | --- | --- |
|  | Event | Incidence rate (per 1,000 person-years) | Adjusted HR^*^ | 95% CI | P-value | Event | Incidence rate (per 1,000 person-years) | Adjusted HR^*^ | 95% CI | P-value |
| **Hip fracture** |  |  |  |  |  |  |  |  |  |  |
| ***Individual MetS-related traits***^†^ | |  |  |  |  |  |  |  |  |  |
| No albuminuria | 882 | 1.30 | Reference | | | 1,970 | 2.21 | Reference | | |
| Albuminuria | 701 | 2.90 | 1.455 | (1.301, 1.628) | <0.001 | 1,647 | 4.52 | 1.346 | (1.253, 1.446) | <0.001 |
| No hypertension | 164 | 0.79 | Reference | | | 192 | 1.14 | Reference | | |
| Hypertension | 1,419 | 1.99 | 1.324 | (1.099, 1.593) | 0.003 | 3,425 | 3.15 | 1.391 | (1.188, 1.629) | <0.001 |
| No hypertriglyceridemia | 1,108 | 1.81 | Reference | | | 2,599 | 3.04 | Reference | | |
| Hypertriglyceridemia | 475 | 1.54 | 0.959 | (0.850, 1.082) | 0.496 | 1,018 | 2.54 | 0.875 | (0.809, 0.947) | <0.001 |
| No low HDL-C level | 1,413 | 1.75 | Reference | | | 3,082 | 2.84 | Reference | | |
| Low HDL-C level | 170 | 1.50 | 0.923 | (0.774, 1.100) | 0.369 | 535 | 3.11 | 1.039 | (0.941, 1.147) | 0.453 |
| No obesity | 966 | 2.53 | Reference | | | 2,105 | 3.88 | Reference | | |
| Obesity | 617 | 1.15 | 0.508 | (0.455, 0.568) | <0.001 | 1,512 | 2.12 | 0.638 | (0.595, 0.684) | <0.001 |
|  |  |  |  |  |  |  |  |  |  |  |
| ***Other covariates at baseline*** | |  |  |  |  |  |  |  |  |  |
| HbA1c < 9% | 1,480 | 1.70 | Reference | | | 3,361 | 2.87 | Reference | | |
| HbA1c ≥ 9% | 103 | 2.02 | 1.096 | (0.866, 1.386) | 0.448 | 256 | 3.02 | 1.296 | (1.124, 1.495) | <0.001 |
|  |  |  |  |  |  |  |  |  |  |  |
| **Major osteoporotic fracture** | |  |  |  |  |  |  |  |  |  |
| ***Individual MetS-related traits***^†^ | |  |  |  |  |  |  |  |  |  |
| No albuminuria | 2,160 | 3.21 | Reference | | | 4,124 | 4.66 | Reference | | |
| Albuminuria | 1,233 | 5.14 | 1.208 | (1.117, 1.307) | <0.001 | 2,755 | 7.65 | 1.192 | (1.130, 1.257) | <0.001 |
| No hypertension | 480 | 2.33 | Reference | | | 488 | 2.91 | Reference | | |
| Hypertension | 2,913 | 4.12 | 1.142 | (1.024, 1.274) | 0.017 | 6,391 | 5.93 | 1.271 | (1.150, 1.405) | <0.001 |
| No hypertriglyceridemia | 2,329 | 3.84 | Reference | | | 4,858 | 5.73 | Reference | | |
| Hypertriglyceridemia | 1,064 | 3.47 | 0.970 | (0.895, 1.052) | 0.465 | 2,021 | 5.09 | 0.911 | (0.861, 0.964) | 0.001 |
| No low HDL-C level | 3,026 | 3.78 | Reference | | | 5,923 | 5.51 | Reference | | |
| Low HDL-C level | 367 | 3.27 | 0.914 | (0.811, 1.029) | 0.137 | 956 | 5.60 | 0.991 | (0.920, 1.066) | 0.800 |
| No obesity | 1,789 | 4.72 | Reference | | | 3,592 | 6.69 | Reference | | |
| Obesity | 1,604 | 3.00 | 0.711 | (0.661, 0.765) | <0.001 | 3,287 | 4.64 | 0.785 | (0.747, 0.825) | <0.001 |
|  |  |  |  |  |  |  |  |  |  |  |
| ***Other covariates at baseline*** | |  |  |  |  |  |  |  |  |  |
| HbA1c < 9% | 3,179 | 3.69 | Reference | | | 6,402 | 5.51 | Reference | | |
| HbA1c ≥ 9% | 214 | 4.23 | 1.178 | (1.005, 1.379) | 0.043 | 477 | 5.69 | 1.204 | (1.085, 1.335) | <0.001 |

Notes: HR = Hazard ratio; CI = Confidence interval; MetS = Metabolic syndrome; T2D = Type 2 diabetes; HbA1c = Glycated hemoglobin; HDL = High-density lipoprotein.

* The models were adjusted by age, sex, smoking status, cardiovascular disease, hyperlipidemia, chronic kidney disease, chronic obstructive pulmonary disease, liver disease, rheumatoid arthritis and other inflammatory polyarthropathies, history of falls (as a proxy indicator for frailty), osteoporosis, hyperparathyroidism, dementia, diabetic retinopathy, severe hypoglycemia, use of antidiabetic medications and systemic glucocorticoids, and baseline HbA1c level.

† MetS-related traits include albuminuria and individual MetS traits (obesity, hypertension, low HDL-cholesterol, and hypertriglyceridemia).

## Supplementary Table 10. Risk of fractures evaluated using pooled logistic regression by obese status, accounting for the development of additional MetS-related traits over time.

| Outcome | Obese | | | Non-obese | | |
| --- | --- | --- | --- | --- | --- | --- |
|  | Adjusted HR^*^ | 95% CI | P-value | Adjusted HR^*^ | 95% CI | P-value |
| **Hip fracture** |  |  |  |  |  |  |
| ***Number of MetS-related traits***^†^ |  |  |  |  |  |  |
| 1 (T2D only) | NA | NA | NA | Reference | | |
| 2 | Reference | | | 1.833 | (1.287, 2.611) | <0.001 |
| 3 | 1.215 | (0.444, 3.323) | 0.705 | 2.472 | (1.740, 3.514) | <0.001 |
| 4 | 0.847 | (0.316, 2.273) | 0.742 | 3.007 | (2.080, 4.347) | <0.001 |
| 5 | 0.750 | (0.279, 2.018) | 0.569 | 2.650 | (1.678, 4.187) | <0.001 |
| 6 | 0.907 | (0.333, 2.465) | 0.848 | NA | NA | NA |
|  |  |  |  |  |  |  |
| **Major osteoporotic fracture** |  |  |  |  |  |  |
| ***Number of MetS-related traits***^†^ |  |  |  |  |  |  |
| 1 (T2D only) | NA | NA | NA | Reference | | |
| 2 | Reference | | | 1.714 | (1.382, 2.126) | <0.001 |
| 3 | 1.258 | (0.684, 2.313) | 0.460 | 2.024 | (1.629, 2.514) | <0.001 |
| 4 | 1.130 | (0.623, 2.049) | 0.687 | 2.209 | (1.745, 2.795) | <0.001 |
| 5 | 0.948 | (0.522, 1.723) | 0.862 | 1.847 | (1.336, 2.553) | <0.001 |
| 6 | 1.102 | (0.602, 2.016) | 0.753 | NA | NA | NA |

Notes: HR = Hazard ratio; CI = Confidence interval; MetS = Metabolic syndrome; T2D = Type 2 diabetes; NA = Not available.

* The models were adjusted by age, sex, smoking status, cardiovascular disease, hyperlipidemia, chronic kidney disease, chronic obstructive pulmonary disease, liver disease, rheumatoid arthritis and other inflammatory polyarthropathies, history of falls (as a proxy indicator for frailty), osteoporosis, hyperparathyroidism, dementia, diabetic retinopathy, severe hypoglycemia, use of antidiabetic medications and systemic glucocorticoids, and baseline HbA1c level.

† MetS-related traits include albuminuria and individual MetS traits (obesity, hypertension, low HDL-cholesterol, and hypertriglyceridemia).

## Supplementary Table 11. Baseline characteristics of patients with complete and incomplete data before and after weighting.

| **Baseline characteristics** | **Complete cohort** | **Incomplete cohort** | **SMD** | **Complete cohort** | **Incomplete cohort** | **SMD** |
| --- | --- | --- | --- | --- | --- | --- |
|  | **(N = 165,289)** | **(N = 569,452)** |  | **(N = 165,289)** | **(N = 569,452)** |  |
| Age at index (years), median (IQR) or n (%) | 60.0 (53.0–68.0) | 64.0 (55.0–73.0) | 0.214 | 61.0 (53.0–69.0) | 63.0 (54.0–72.0) | 0.122 |
| < 50 | 27,176 (16.4) | 79,543 (14.0) |  | 119,963 (17.2) | 105,140 (14.3) |  |
| 50-74 | 118,796 (71.9) | 369,456 (64.9) |  | 468,882 (67.3) | 485,204 (66.1) |  |
| ≥ 75 | 19,317 (11.7) | 120,453 (21.2) |  | 108,423 (15.6) | 143,970 (19.6) |  |
| Male, n (%) | 89,628 (54.2) | 285,684 (50.2) | 0.081 | 87,561 (53.0) | 290,879 (51.1) | 0.038 |
| Pre-existing comorbidities, median (IQR) or n (%) |  |  |  |  |  |  |
| Cardiovascular disease | 29,425 (17.8) | 163,111 (28.6) | 0.259 | 42,341 (25.6) | 149,860 (26.3) | 0.016 |
| Hyperlipidemia | 86,561 (52.4) | 142,441 (25.0) | 0.585 | 54,686 (33.1) | 177,285 (31.1) | 0.042 |
| Chronic kidney disease | 15,102 (9.1) | 93,755 (16.5) | 0.221 | 27,164 (16.4) | 84,613 (14.9) | 0.043 |
| Chronic obstructive pulmonary disease | 2,656 (1.6) | 17,955 (3.2) | 0.102 | 4,467 (2.7) | 16,007 (2.8) | 0.007 |
| Liver disease | 484 (0.3) | 4,018 (0.7) | 0.059 | 1,172 (0.7) | 3,499 (0.6) | 0.012 |
| Rheumatoid arthritis and other inflammatory polyarthropathies | 236 (0.1) | 1,891 (0.3) | 0.039 | 523 (0.3) | 1,651 (0.3) | 0.005 |
| History of falls | 5,072 (3.1) | 18,727 (3.3) | 0.013 | 5,385 (3.3) | 18,443 (3.2) | 0.001 |
| Osteoporosis | 342 (0.2) | 2,085 (0.4) | 0.030 | 657 (0.4) | 1,898 (0.3) | 0.011 |
| Hyperparathyroidism | 120 (0.1) | 619 (0.1) | 0.012 | 143 (0.1) | 575 (0.1) | 0.005 |
| Dementia | 367 (0.2) | 4,717 (0.8) | 0.084 | 992 (0.6) | 3,949 (0.7) | 0.012 |
| Diabetic retinopathy | 2,950 (1.8) | 5,811 (1.0) | 0.065 | 1,967 (1.2) | 6,512 (1.1) | 0.004 |
| Severe hypoglycemia | 6,933 (4.2) | 30,138 (5.3) | 0.052 | 9,640 (5.8) | 28,825 (5.1) | 0.034 |
| Use of medications, n (%) |  |  |  |  |  |  |
| Insulin | 5,483 (3.3) | 19,326 (3.4) | 0.004 | 8,429 (5.1) | 19,493 (3.4) | 0.083 |
| Metformin | 105,881 (64.1) | 207,447 (36.4) | 0.575 | 76,947 (46.6) | 242,833 (42.6) | 0.079 |
| Sulfonylurea | 46,039 (27.9) | 188,830 (33.2) | 0.115 | 55,879 (33.8) | 181,425 (31.9) | 0.041 |
| Thiazolidinedione | 1,152 (0.7) | 1,101 (0.2) | 0.076 | 751 (0.5) | 1,643 (0.3) | 0.027 |
| Dipeptidyl peptidase 4 inhibitors | 2,582 (1.6) | 2,123 (0.4) | 0.122 | 1,735 (1.0) | 3,554 (0.6) | 0.047 |
| SGLT2 inhibitors | 389 (0.2) | 296 (0.1) | 0.048 | 264 (0.2) | 510 (0.1) | 0.020 |
| Glucagon-like peptide-1 receptor agonist | 70 (0.0) | 21 (0.0) | 0.025 | 29 (0.0) | 41 (0.0) | 0.009 |
| α-Glucosidase inhibitors | 160 (0.1) | 4,271 (0.8) | 0.101 | 1,483 (0.9) | 3,443 (0.6) | 0.034 |
| Systemic glucocorticoids | 5,514 (3.3) | 26,887 (4.7) | 0.071 | 8,409 (5.1) | 25,206 (4.4) | 0.031 |

Notes: SMD = Standardised mean difference; IQR = Interquartile range; SGLT2 = Sodium–glucose cotransporter 2.

## Supplementary Table 12. Incidence rates and HRs for fractures associated with albuminuria and MetS traits present at baseline in obese and non-obese patients with T2D, weighted by inverse probability of having complete baseline MetS data.

| Outcome | **Obese** | | | **Non-obese** | | |
| --- | --- | --- | --- | --- | --- | --- |
|  | Adjusted HR^*^ | 95% CI | P-value | Adjusted HR^*^ | 95% CI | P-value |
| **Hip fracture** |  |  |  |  |  |  |
| ***Number of MetS-related traits***^†^ |  |  |  |  |  |  |
| 1 (T2D only) | NA | | | Reference | | |
| 2 | Reference | | | 1.837 | (1.297, 2.600) | <0.001 |
| 3 | 1.104 | (0.623, 1.956) | 0.735 | 2.018 | (1.417, 2.876) | <0.001 |
| 4 | 1.384 | (0.779, 2.458) | 0.268 | 2.264 | (1.513, 3.389) | <0.001 |
| 5 | 1.225 | (0.666, 2.254) | 0.514 | 1.655 | (0.904, 3.030) | 0.103 |
| 6 | 1.359 | (0.648, 2.854) | 0.417 | NA | | |
| ≥3 or ≥2^‡^ | 1.236 | (0.707, 2.163) | 0.457 | 1.942 | (1.385, 2.723) | <0.001 |
| Continuous | 1.075 | (0.958, 1.205) | 0.218 | 1.140 | (1.048, 1.240) | 0.002 |
| ***Individual MetS-related traits***^†^ |  |  |  |  |  |  |
| No albuminuria | Reference | | | Reference | | |
| Albuminuria | 1.317 | (1.058, 1.639) | 0.014 | 1.394 | (1.172, 1.659) | <0.001 |
| No hypertension | Reference | | | Reference | | |
| Hypertension | 1.194 | (0.780, 1.829) | 0.414 | 1.332 | (1.022, 1.736) | 0.034 |
| No hypertriglyceridemia | Reference | | | Reference | | |
| Hypertriglyceridemia | 0.911 | (0.731, 1.135) | 0.406 | 0.988 | (0.820, 1.190) | 0.898 |
| No low HDL-C level | Reference | | | Reference | | |
| Low HDL-C level | 0.999 | (0.744, 1.342) | 0.994 | 0.900 | (0.681, 1.188) | 0.456 |
|  |  |  |  |  |  |  |
| **Fracture** |  |  |  |  |  |  |
| ***Number of MetS-related traits***^†^ |  |  |  |  |  |  |
| 1 (T2D only) | NA | | | Reference | | |
| 2 | Reference | | | 1.452 | (1.176, 1.791) | <0.001 |
| 3 | 0.947 | (0.721, 1.244) | 0.696 | 1.565 | (1.257, 1.948) | <0.001 |
| 4 | 1.024 | (0.778, 1.349) | 0.864 | 1.426 | (1.090, 1.866) | 0.010 |
| 5 | 0.996 | (0.738, 1.345) | 0.980 | 1.269 | (0.825, 1.951) | 0.278 |
| 6 | 0.919 | (0.597, 1.414) | 0.700 | NA | | |
| ≥3 or ≥2^‡^ | 0.983 | (0.756, 1.278) | 0.898 | 1.483 | (1.212, 1.816) | <0.001 |
| Continuous | 1.012 | (0.943, 1.085) | 0.747 | 1.065 | (1.001, 1.133) | 0.045 |
| ***Individual MetS-related traits***^†^ |  |  |  |  |  |  |
| No albuminuria | Reference | | | Reference | | |
| Albuminuria | 1.130 | (0.985, 1.298) | 0.082 | 1.211 | (1.063, 1.380) | 0.004 |
| No hypertension | Reference | | | Reference | | |
| Hypertension | 0.918 | (0.747, 1.130) | 0.420 | 1.225 | (1.031, 1.455) | 0.021 |
| No hypertriglyceridemia | Reference | | | Reference | | |
| Hypertriglyceridemia | 0.929 | (0.814, 1.060) | 0.271 | 0.982 | (0.855, 1.126) | 0.790 |
| No low HDL-C level | Reference | | | Reference | | |
| Low HDL-C level | 1.053 | (0.875, 1.267) | 0.583 | 0.836 | (0.676, 1.034) | 0.098 |

Notes: HR = Hazard ratio; CI = Confidence interval; MetS = Metabolic syndrome; T2D = Type 2 diabetes; HbA1c = Glycated hemoglobin; HDL = High-density lipoprotein; NA = Not available.

* The models were adjusted by age, sex, smoking status, cardiovascular disease, hyperlipidemia, chronic kidney disease, chronic obstructive pulmonary disease, liver disease, rheumatoid arthritis and other inflammatory polyarthropathies, history of falls (as a proxy indicator for frailty), osteoporosis, hyperparathyroidism, dementia, diabetic retinopathy, severe hypoglycemia, use of antidiabetic medications and systemic glucocorticoids, and baseline HbA1c level.

† MetS-related traits include albuminuria and individual MetS traits (obesity, hypertension, low HDL-cholesterol, and hypertriglyceridemia).

‡ ≥2 MetS-related traits indicate the presence of at least one additional MetS-related trait beyond T2D, while ≥3 MetS-related traits indicate at least one additional MetS-related trait beyond T2D and obesity.

## Supplementary Table 13. Incidence rates and HRs for fractures associated with albuminuria and MetS traits present at baseline in obese and non-obese patients with T2D, including patients with prior fracture history.

| Outcome | **Obese (N = 98,353)** | | | | | **Non-obese (N = 70,724)** | | | | |
| --- | --- | --- | --- | --- | --- | --- | --- | --- | --- | --- |
|  | Event | Incidence rate (per 1,000 person-years) | Adjusted HR^*^ | 95% CI | P-value | Event | Incidence rate (per 1,000 person-years) | Adjusted HR^*^ | 95% CI | P-value |
| **Hip fracture** |  |  |  |  |  |  |  |  |  |  |
| ***Number of MetS-related traits***^†^ |  |  |  |  |  |  |  |  |  |  |
| 1 (T2D only) | NA | | | | | 65 | 0.82 | Reference | | |
| 2 | 26 | 0.55 | Reference | | | 403 | 2.44 | 1.875 | (1.402, 2.507) | <0.001 |
| 3 | 212 | 1.00 | 1.140 | (0.715, 1.817) | 0.583 | 414 | 3.87 | 2.226 | (1.657, 2.990) | <0.001 |
| 4 | 304 | 1.61 | 1.568 | (0.988, 2.491) | 0.057 | 145 | 4.43 | 2.336 | (1.682, 3.245) | <0.001 |
| 5 | 117 | 1.40 | 1.240 | (0.757, 2.032) | 0.392 | 24 | 4.16 | 1.884 | (1.137, 3.121) | 0.014 |
| 6 | 29 | 1.80 | 1.667 | (0.907, 3.065) | 0.100 | NA | | | | |
| ≥3 or ≥2^‡^ | 662 | 1.32 | 1.332 | (0.846, 2.095) | 0.215 | 986 | 3.18 | 2.042 | (1.540, 2.708) | <0.001 |
| Continuous | NA | | 1.107 | (1.010, 1.214) | 0.030 | NA | | 1.176 | (1.095, 1.264) | <0.001 |
| ***Individual MetS-related traits***^†^ |  |  |  |  |  |  |  |  |  |  |
| No albuminuria | 390 | 0.99 | Reference | | | 580 | 1.95 | Reference | | |
| Albuminuria | 298 | 1.93 | 1.352 | (1.140, 1.604) | <0.001 | 471 | 5.11 | 1.510 | (1.316, 1.732) | <0.001 |
| No hypertension | 47 | 0.50 | Reference | | | 128 | 1.10 | Reference | | |
| Hypertension | 641 | 1.41 | 1.245 | (0.887, 1.747) | 0.205 | 923 | 3.38 | 1.388 | (1.123, 1.714) | 0.002 |
| No hypertriglyceridemia | 435 | 1.31 | Reference | | | 778 | 2.67 | Reference | | |
| Hypertriglyceridemia | 253 | 1.17 | 0.901 | (0.756, 1.075) | 0.247 | 273 | 2.77 | 1.031 | (0.886, 1.200) | 0.693 |
| No low HDL-C level | 593 | 1.27 | Reference | | | 956 | 2.70 | Reference | | |
| Low HDL-C level | 95 | 1.20 | 1.092 | (0.858, 1.390) | 0.472 | 95 | 2.64 | 0.820 | (0.652, 1.030) | 0.088 |
|  |  |  |  |  |  |  |  |  |  |  |
| **Fracture** |  |  |  |  |  |  |  |  |  |  |
| ***Number of MetS-related traits***^†^ |  |  |  |  |  |  |  |  |  |  |
| 1 (T2D only) | NA | | | | | 185 | 2.34 | Reference | | |
| 2 | 108 | 2.28 | Reference | | | 781 | 4.76 | 1.443 | (1.213, 1.717) | <0.001 |
| 3 | 615 | 2.93 | 0.915 | (0.735, 1.140) | 0.430 | 705 | 6.65 | 1.641 | (1.372, 1.963) | <0.001 |
| 4 | 696 | 3.71 | 1.054 | (0.846, 1.313) | 0.642 | 222 | 6.85 | 1.494 | (1.203, 1.856) | <0.001 |
| 5 | 267 | 3.22 | 0.904 | (0.708, 1.153) | 0.416 | 43 | 7.55 | 1.453 | (1.014, 2.081) | 0.042 |
| 6 | 59 | 3.69 | 1.008 | (0.710, 1.433) | 0.963 | NA | | | | |
| ≥3 or ≥2^‡^ | 1,637 | 3.30 | 0.967 | (0.783, 1.195) | 0.758 | 1,751 | 5.69 | 1.514 | (1.282, 1.789) | <0.001 |
| Continuous | NA | | 1.013 | (0.958, 1.072) | 0.653 | NA | | 1.103 | (1.047, 1.163) | <0.001 |
| ***Individual MetS-related traits***^†^ |  |  |  |  |  |  |  |  |  |  |
| No albuminuria | 1,129 | 2.89 | Reference | | | 1,208 | 4.08 | Reference | | |
| Albuminuria | 616 | 4.01 | 1.134 | (1.017, 1.264) | 0.024 | 728 | 7.97 | 1.274 | (1.148, 1.414) | <0.001 |
| No hypertension | 194 | 2.09 | Reference | | | 310 | 2.67 | Reference | | |
| Hypertension | 1,551 | 3.44 | 0.977 | (0.827, 1.153) | 0.780 | 1,626 | 6.00 | 1.291 | (1.124, 1.483) | <0.001 |
| No hypertriglyceridemia | 1,095 | 3.32 | Reference | | | 1,430 | 4.94 | Reference | | |
| Hypertriglyceridemia | 650 | 3.03 | 0.941 | (0.846, 1.047) | 0.263 | 506 | 5.17 | 1.026 | (0.918, 1.147) | 0.653 |
| No low HDL-C level | 1,518 | 3.26 | Reference | | | 1,767 | 5.03 | Reference | | |
| Low HDL-C level | 227 | 2.88 | 0.995 | (0.855, 1.160) | 0.954 | 169 | 4.74 | 0.804 | (0.676, 0.957) | 0.014 |

Notes: HR = Hazard ratio; CI = Confidence interval; MetS = Metabolic syndrome; T2D = Type 2 diabetes; HbA1c = Glycated hemoglobin; HDL = High-density lipoprotein; NA = Not available.

* The models were adjusted by age, sex, smoking status, cardiovascular disease, hyperlipidemia, chronic kidney disease, chronic obstructive pulmonary disease, liver disease, rheumatoid arthritis and other inflammatory polyarthropathies, history of falls (as a proxy indicator for frailty), history of fractures, osteoporosis, hyperparathyroidism, dementia, diabetic retinopathy, severe hypoglycemia, use of antidiabetic medications and systemic glucocorticoids, and baseline HbA1c level.

† MetS-related traits include albuminuria and individual MetS traits (obesity, hypertension, low HDL-cholesterol, and hypertriglyceridemia).

‡ ≥2 MetS-related traits indicate the presence of at least one additional MetS-related trait beyond T2D, while ≥3 MetS-related traits indicate at least one additional MetS-related trait beyond T2D and obesity.

## Supplementary Table 14. Competing risk regression analyses for fractures associated with the albuminuria and MetS traits present at baseline in obese and non-obese patients with T2D.

| Outcome | **Obese** | | | **Non-obese** | | |
| --- | --- | --- | --- | --- | --- | --- |
|  | Adjusted HR^*^ | 95% CI | P-value | Adjusted HR^*^ | 95% CI | P-value |
| **Hip fracture** |  |  |  |  |  |  |
| ***Number of MetS-related traits***^†^ |  |  |  |  |  |  |
| 1 (T2D only) | NA | | | Reference | | |
| 2 | Reference | | | 1.802 | (1.338, 2.428) | <0.001 |
| 3 | 1.178 | (0.719, 1.931) | 0.515 | 2.111 | (1.558, 2.861) | <0.001 |
| 4 | 1.484 | (0.905, 2.432) | 0.118 | 2.126 | (1.507, 2.999) | <0.001 |
| 5 | 1.245 | (0.734, 2.109) | 0.416 | 1.768 | (1.046, 2.988) | 0.033 |
| 6 | 1.618 | (0.851, 3.077) | 0.142 | NA | | |
| ≥3 or ≥2^‡^ | 1.315 | (0.812, 2.132) | 0.265 | 1.940 | (1.452, 2.592) | <0.001 |
| Continuous | 1.085 | (0.986, 1.195) | 0.095 | 1.154 | (1.074, 1.239) | <0.001 |
| ***Individual MetS-related traits***^†^ |  |  |  |  |  |  |
| No albuminuria | Reference | | | Reference | | |
| Albuminuria | 1.249 | (1.036, 1.506) | 0.020 | 1.450 | (1.247, 1.686) | <0.001 |
| No hypertension | Reference | | | Reference | | |
| Hypertension | 1.243 | (0.867, 1.782) | 0.238 | 1.358 | (1.088, 1.694) | 0.007 |
| No hypertriglyceridemia | Reference | | | Reference | | |
| Hypertriglyceridemia | 0.920 | (0.763, 1.108) | 0.378 | 1.043 | (0.891, 1.221) | 0.600 |
| No low HDL-C level | Reference | | | Reference | | |
| Low HDL-C level | 1.091 | (0.847, 1.405) | 0.500 | 0.774 | (0.607, 0.987) | 0.039 |
|  |  |  |  |  |  |  |
| **Major osteoporotic fracture** | |  |  |  |  |  |
| ***Number of MetS-related traits***^†^ |  |  |  |  |  |  |
| 1 (T2D only) | NA | | | Reference | | |
| 2 | Reference | | | 1.395 | (1.169, 1.664) | <0.001 |
| 3 | 0.932 | (0.742, 1.169) | 0.542 | 1.548 | (1.289, 1.859) | <0.001 |
| 4 | 1.014 | (0.806, 1.276) | 0.903 | 1.384 | (1.104, 1.735) | 0.005 |
| 5 | 0.914 | (0.711, 1.176) | 0.486 | 1.367 | (0.942, 1.984) | 0.100 |
| 6 | 0.962 | (0.667, 1.387) | 0.836 | NA | | |
| ≥3 or ≥2^‡^ | 0.960 | (0.772, 1.195) | 0.718 | 1.444 | (1.219, 1.711) | <0.001 |
| Continuous | 1.000 | (0.944, 1.060) | 0.992 | 1.083 | (1.028, 1.142) | 0.003 |
| ***Individual MetS-related traits***^†^ |  |  |  |  |  |  |
| No albuminuria | Reference | | | Reference | | |
| Albuminuria | 1.085 | (0.967, 1.218) | 0.163 | 1.217 | (1.089, 1.359) | <0.001 |
| No hypertension | Reference | | | Reference | | |
| Hypertension | 0.973 | (0.820, 1.156) | 0.759 | 1.240 | (1.075, 1.430) | 0.003 |
| No hypertriglyceridemia | Reference | | | Reference | | |
| Hypertriglyceridemia | 0.945 | (0.846, 1.056) | 0.321 | 1.042 | (0.929, 1.170) | 0.479 |
| No low HDL-C level | Reference | | | Reference | | |
| Low HDL-C level | 0.995 | (0.850, 1.165) | 0.954 | 0.790 | (0.659, 0.947) | 0.011 |

Notes: HR = Hazard ratio; CI = Confidence interval; MetS = Metabolic syndrome; T2D = Type 2 diabetes; HbA1c = Glycated hemoglobin; HDL = High-density lipoprotein; NA = Not available.

* The models were adjusted by age, sex, smoking status, cardiovascular disease, hyperlipidemia, chronic kidney disease, chronic obstructive pulmonary disease, liver disease, rheumatoid arthritis and other inflammatory polyarthropathies, history of falls (as a proxy indicator for frailty), osteoporosis, hyperparathyroidism, dementia, diabetic retinopathy, severe hypoglycemia, use of antidiabetic medications and systemic glucocorticoids, and baseline HbA1c level.

† MetS-related traits include albuminuria and individual MetS traits (obesity, hypertension, low HDL-cholesterol, and hypertriglyceridemia).

‡ ≥2 MetS-related traits indicate the presence of at least one additional MetS-related trait beyond T2D, while ≥3 MetS-related traits indicate at least one additional MetS-related trait beyond T2D and obesity.

## Supplementary Table 15. Incidence rates and HRs for fractures associated with albuminuria and MetS traits present at baseline in obese and non-obese patients with T2D, excluding patients diagnosed solely by a fasting glucose ≥7.0 mmol/L.

| Outcome | **Obese (N = 94,891)** | | | | | **Non-obese (N = 68,037)** | | | | |
| --- | --- | --- | --- | --- | --- | --- | --- | --- | --- | --- |
|  | Event | Incidence rate (per 1,000 person-years) | Adjusted HR^*^ | 95% CI | P-value | Event | Incidence rate (per 1,000 person-years) | Adjusted HR^*^ | 95% CI | P-value |
| **Hip fracture** |  |  |  |  |  |  |  |  |  |  |
| ***Number of MetS-related traits***^†^ |  |  |  |  |  |  |  |  |  |  |
| 1 (T2D only) | NA | | | | | 63 | 0.81 | Reference | | |
| 2 | 24 | 0.52 | Reference | | | 365 | 2.29 | 1.810 | (1.345, 2.437) | <0.001 |
| 3 | 194 | 0.95 | 1.168 | (0.715, 1.910) | 0.535 | 373 | 3.64 | 2.140 | (1.582, 2.894) | <0.001 |
| 4 | 254 | 1.40 | 1.479 | (0.907, 2.413) | 0.117 | 125 | 3.99 | 2.153 | (1.532, 3.026) | <0.001 |
| 5 | 105 | 1.31 | 1.304 | (0.775, 2.192) | 0.317 | 23 | 4.19 | 1.922 | (1.147, 3.221) | 0.013 |
| 6 | 28 | 1.81 | 1.750 | (0.929, 3.296) | 0.083 | NA | | | | |
| ≥3 or ≥2^‡^ | 581 | 1.21 | 1.322 | (0.819, 2.132) | 0.253 | 886 | 2.97 | 1.960 | (1.469, 2.614) | <0.001 |
| Continuous | NA | | 1.111 | (1.007, 1.225) | 0.036 | NA | | 1.167 | (1.082, 1.258) | <0.001 |
| ***Individual MetS-related traits***^†^ |  |  |  |  |  |  |  |  |  |  |
| No albuminuria | 344 | 0.91 | Reference | | | 526 | 1.83 | Reference | | |
| Albuminuria | 261 | 1.76 | 1.342 | (1.118, 1.610) | 0.002 | 423 | 4.79 | 1.528 | (1.322, 1.765) | <0.001 |
| No hypertension | 43 | 0.47 | Reference | | | 120 | 1.05 | Reference | | |
| Hypertension | 562 | 1.29 | 1.246 | (0.871, 1.781) | 0.229 | 829 | 3.16 | 1.375 | (1.105, 1.711) | 0.004 |
| No hypertriglyceridemia | 384 | 1.20 | Reference | | | 703 | 2.50 | Reference | | |
| Hypertriglyceridemia | 221 | 1.07 | 0.904 | (0.748, 1.092) | 0.295 | 246 | 2.59 | 1.014 | (0.864, 1.188) | 0.868 |
| No low HDL-C level | 520 | 1.15 | Reference | | | 869 | 2.55 | Reference | | |
| Low HDL-C level | 85 | 1.11 | 1.121 | (0.867, 1.448) | 0.384 | 80 | 2.30 | 0.775 | (0.605, 0.993) | 0.044 |
|  |  |  |  |  |  |  |  |  |  |  |
| **Fracture** |  |  |  |  |  |  |  |  |  |  |
| ***Number of MetS-related traits***^†^ |  |  |  |  |  |  |  |  |  |  |
| 1 (T2D only) | NA | | | | | 180 | 2.33 | Reference | | |
| 2 | 100 | 2.16 | Reference | | | 705 | 4.46 | 1.382 | (1.158, 1.650) | <0.001 |
| 3 | 572 | 2.83 | 0.928 | (0.739, 1.164) | 0.517 | 632 | 6.22 | 1.567 | (1.304, 1.882) | <0.001 |
| 4 | 606 | 3.37 | 1.013 | (0.806, 1.273) | 0.910 | 197 | 6.34 | 1.391 | (1.111, 1.743) | 0.004 |
| 5 | 246 | 3.09 | 0.928 | (0.722, 1.194) | 0.563 | 41 | 7.56 | 1.468 | (1.015, 2.122) | 0.041 |
| 6 | 55 | 3.58 | 1.013 | (0.705, 1.457) | 0.943 | NA | | | | |
| ≥3 or ≥2^‡^ | 1,479 | 3.10 | 0.962 | (0.773, 1.196) | 0.726 | 1,575 | 5.32 | 1.446 | (1.220, 1.714) | <0.001 |
| Continuous | NA | | 1.010 | (0.952, 1.071) | 0.745 | NA | | 1.096 | (1.037, 1.158) | 0.001 |
| ***Individual MetS-related traits***^†^ |  |  |  |  |  |  |  |  |  |  |
| No albuminuria | 1,025 | 2.73 | Reference | | | 1,103 | 3.86 | Reference | | |
| Albuminuria | 554 | 3.75 | 1.127 | (1.005, 1.264) | 0.040 | 652 | 7.46 | 1.275 | (1.142, 1.423) | <0.001 |
| No hypertension | 181 | 2.00 | Reference | | | 296 | 2.61 | Reference | | |
| Hypertension | 1,398 | 3.23 | 0.970 | (0.817, 1.151) | 0.726 | 1,459 | 5.61 | 1.253 | (1.087, 1.445) | 0.002 |
| No hypertriglyceridemia | 998 | 3.14 | Reference | | | 1,293 | 4.63 | Reference | | |
| Hypertriglyceridemia | 581 | 2.82 | 0.930 | (0.831, 1.041) | 0.205 | 462 | 4.90 | 1.019 | (0.907, 1.145) | 0.745 |
| No low HDL-C level | 1,370 | 3.06 | Reference | | | 1,604 | 4.73 | Reference | | |
| Low HDL-C level | 209 | 2.75 | 1.018 | (0.868, 1.194) | 0.825 | 151 | 4.38 | 0.794 | (0.661, 0.954) | 0.014 |

Notes: HR = Hazard ratio; CI = Confidence interval; MetS = Metabolic syndrome; T2D = Type 2 diabetes; HbA1c = Glycated hemoglobin; HDL = High-density lipoprotein; NA = Not available.

*The models were adjusted by age, sex, smoking status, cardiovascular disease, hyperlipidemia, chronic kidney disease, chronic obstructive pulmonary disease, liver disease, rheumatoid arthritis and other inflammatory polyarthropathies, history of falls (as a proxy indicator for frailty), osteoporosis, hyperparathyroidism, dementia, diabetic retinopathy, severe hypoglycemia, use of antidiabetic medications and systemic glucocorticoids, and baseline HbA1c level.

† MetS-related traits include albuminuria and individual MetS traits (obesity, hypertension, low HDL-cholesterol, and hypertriglyceridemia).

‡ ≥2 MetS-related traits indicate the presence of at least one additional MetS-related trait beyond T2D, while ≥3 MetS-related traits indicate at least one additional MetS-related trait beyond T2D and obesity.

## Supplementary Table 16. Incidence rates and HRs for fractures associated with albuminuria and MetS traits present at baseline in obese and non-obese patients with T2D, excluding those with post-pancreatitis diabetes mellitus.

| Outcome | **Obese (N = 95,910)** | | | | | **Non-obese (N = 68,644)** | | | | |
| --- | --- | --- | --- | --- | --- | --- | --- | --- | --- | --- |
|  | Event | Incidence rate (per 1,000 person-years) | Adjusted HR^*^ | 95% CI | P-value | Event | Incidence rate (per 1,000 person-years) | Adjusted HR^*^ | 95% CI | P-value |
| **Hip fracture** |  |  |  |  |  |  |  |  |  |  |
| ***Number of MetS-related traits***^†^ | |  |  |  |  |  |  |  |  |  |
| 1 (T2D only) | NA | | | | | 63 | 0.81 | Reference | | |
| 2 | 23 | 0.49 | Reference | | | 367 | 2.28 | 1.802 | (1.338, 2.426) | <0.001 |
| 3 | 196 | 0.95 | 1.223 | (0.739, 2.024) | 0.434 | 382 | 3.67 | 2.141 | (1.584, 2.896) | <0.001 |
| 4 | 261 | 1.41 | 1.570 | (0.951, 2.593) | 0.078 | 127 | 4.00 | 2.177 | (1.551, 3.057) | <0.001 |
| 5 | 106 | 1.30 | 1.330 | (0.781, 2.265) | 0.294 | 23 | 4.11 | 1.887 | (1.126, 3.163) | 0.016 |
| 6 | 28 | 1.78 | 1.799 | (0.945, 3.423) | 0.074 | NA | | | | |
| ≥3 or ≥2^‡^ | 591 | 1.21 | 1.385 | (0.848, 2.263) | 0.193 | 899 | 2.97 | 1.958 | (1.468, 2.611) | <0.001 |
| Continuous | NA | | 1.107 | (1.004, 1.221) | 0.042 | NA | | 1.169 | (1.085, 1.260) | <0.001 |
| ***Individual MetS-related traits***^†^ |  |  |  |  |  |  |  |  |  |  |
| No albuminuria | 349 | 0.91 | Reference | | | 530 | 1.82 | Reference | | |
| Albuminuria | 265 | 1.75 | 1.332 | (1.110, 1.597) | 0.002 | 432 | 4.82 | 1.533 | (1.327, 1.770) | <0.001 |
| No hypertension | 42 | 0.46 | Reference | | | 121 | 1.06 | Reference | | |
| Hypertension | 572 | 1.29 | 1.274 | (0.887, 1.830) | 0.190 | 841 | 3.16 | 1.354 | (1.089, 1.683) | 0.006 |
| No hypertriglyceridemia | 389 | 1.20 | Reference | | | 714 | 2.51 | Reference | | |
| Hypertriglyceridemia | 225 | 1.07 | 0.907 | (0.751, 1.094) | 0.306 | 248 | 2.57 | 1.010 | (0.862, 1.183) | 0.906 |
| No low HDL-C level | 528 | 1.15 | Reference | | | 879 | 2.54 | Reference | | |
| Low HDL-C level | 86 | 1.11 | 1.101 | (0.852, 1.422) | 0.461 | 83 | 2.37 | 0.802 | (0.628, 1.023) | 0.075 |
|  |  |  |  |  |  |  |  |  |  |  |
| **Fracture** |  |  |  |  |  |  |  |  |  |  |
| ***Number of MetS-related traits***^†^ | |  |  |  |  |  |  |  |  |  |
| 1 (T2D only) | NA | | | | | 180 | 2.32 | Reference | | |
| 2 | 99 | 2.12 | Reference | | | 717 | 4.48 | 1.391 | (1.165, 1.661) | <0.001 |
| 3 | 574 | 2.79 | 0.931 | (0.741, 1.169) | 0.538 | 644 | 6.24 | 1.560 | (1.298, 1.873) | <0.001 |
| 4 | 619 | 3.38 | 1.028 | (0.818, 1.293) | 0.810 | 200 | 6.35 | 1.408 | (1.126, 1.762) | 0.003 |
| 5 | 247 | 3.05 | 0.923 | (0.717, 1.189) | 0.535 | 41 | 7.41 | 1.448 | (1.002, 2.094) | 0.049 |
| 6 | 56 | 3.58 | 1.010 | (0.702, 1.453) | 0.959 | NA | | | | |
| ≥3 or ≥2^‡^ | 1,496 | 3.08 | 0.968 | (0.777, 1.205) | 0.770 | 1,602 | 5.33 | 1.450 | (1.223, 1.718) | <0.001 |
| Continuous | NA | | 1.009 | (0.952, 1.070) | 0.763 | NA | | 1.094 | (1.036, 1.156) | 0.001 |
| ***Individual MetS-related traits***^†^ |  |  |  |  |  |  |  |  |  |  |
| No albuminuria | 1,035 | 2.71 | Reference | | | 1,118 | 3.86 | Reference | | |
| Albuminuria | 560 | 3.73 | 1.126 | (1.005, 1.262) | 0.041 | 664 | 7.48 | 1.272 | (1.141, 1.419) | <0.001 |
| No hypertension | 180 | 1.97 | Reference | | | 299 | 2.63 | Reference | | |
| Hypertension | 1,415 | 3.21 | 0.973 | (0.819, 1.156) | 0.757 | 1,483 | 5.61 | 1.236 | (1.072, 1.424) | 0.003 |
| No hypertriglyceridemia | 1,004 | 3.11 | Reference | | | 1,315 | 4.66 | Reference | | |
| Hypertriglyceridemia | 591 | 2.82 | 0.932 | (0.833, 1.043) | 0.220 | 467 | 4.89 | 1.014 | (0.903, 1.139) | 0.812 |
| No low HDL-C level | 1,384 | 3.04 | Reference | | | 1,627 | 4.74 | Reference | | |
| Low HDL-C level | 211 | 2.74 | 1.009 | (0.860, 1.183) | 0.917 | 155 | 4.46 | 0.815 | (0.680, 0.977) | 0.027 |

Notes: HR = Hazard ratio; CI = Confidence interval; MetS = Metabolic syndrome; T2D = Type 2 diabetes; HbA1c = Glycated hemoglobin; HDL = High-density lipoprotein; NA = Not available.

* The models were adjusted by age, sex, smoking status, cardiovascular disease, hyperlipidemia, chronic kidney disease, chronic obstructive pulmonary disease, liver disease, rheumatoid arthritis and other inflammatory polyarthropathies, history of falls (as a proxy indicator for frailty), osteoporosis, hyperparathyroidism, dementia, diabetic retinopathy, severe hypoglycemia, use of antidiabetic medications and systemic glucocorticoids, and baseline HbA1c level.

† MetS-related traits include albuminuria and individual MetS traits (obesity, hypertension, low HDL-cholesterol, and hypertriglyceridemia).

‡ ≥2 MetS-related traits indicate the presence of at least one additional MetS-related trait beyond T2D, while ≥3 MetS-related traits indicate at least one additional MetS-related trait beyond T2D and obesity.

## Supplementary Table 17. Incidence rates and HRs for fractures associated with albuminuria and MetS traits present at baseline in obese and non-obese patients with T2D, where obesity is defined by waist circumference.

| Outcome | **Obese (N = 94,297)** | | | | | **Non-obese (N = 40,841)** | | | | |
| --- | --- | --- | --- | --- | --- | --- | --- | --- | --- | --- |
|  | Event | Incidence rate (per 1,000 person-years) | Adjusted HR^*^ | 95% CI | P-value | Event | Incidence rate (per 1,000 person-years) | Adjusted HR^*^ | 95% CI | P-value |
| **Hip fracture** |  |  |  |  |  |  |  |  |  |  |
| ***Number of MetS-related traits***^†^ |  |  |  |  |  |  |  |  |  |  |
| 1 (T2D only) | NA | | | | | 28 | 0.52 | Reference | | |
| 2 | 28 | 0.55 | Reference | | | 153 | 1.58 | 1.842 | (1.220, 2.780) | 0.004 |
| 3 | 254 | 1.27 | 1.322 | (0.890, 1.962) | 0.167 | 149 | 2.62 | 2.340 | (1.539, 3.558) | <0.001 |
| 4 | 299 | 1.79 | 1.639 | (1.105, 2.433) | 0.014 | 37 | 2.33 | 2.055 | (1.241, 3.402) | 0.005 |
| 5 | 105 | 1.50 | 1.365 | (0.892, 2.089) | 0.152 | 9 | 3.52 | 2.545 | (1.181, 5.483) | 0.017 |
| 6 | 26 | 1.86 | 1.613 | (0.937, 2.776) | 0.084 | NA | | | | |
| ≥3 or ≥2^‡^ | 684 | 1.52 | 1.452 | (0.989, 2.133) | 0.057 | 348 | 2.02 | 2.040 | (1.372, 3.033) | <0.001 |
| Continuous | NA | | 1.078 | (0.992, 1.171) | 0.076 | NA | | 1.213 | (1.082, 1.361) | <0.001 |
| ***Individual MetS-related traits***^†^ |  |  |  |  |  |  |  |  |  |  |
| No albuminuria | 419 | 1.15 | Reference | | | 204 | 1.15 | Reference | | |
| Albuminuria | 293 | 2.16 | 1.313 | (1.123, 1.534) | <0.001 | 172 | 3.56 | 1.742 | (1.394, 2.176) | <0.001 |
| No hypertension | 58 | 0.62 | Reference | | | 51 | 0.66 | Reference | | |
| Hypertension | 654 | 1.60 | 1.205 | (0.910, 1.594) | 0.193 | 325 | 2.19 | 1.456 | (1.059, 2.002) | 0.021 |
| No hypertriglyceridemia | 479 | 1.52 | Reference | | | 307 | 1.76 | Reference | | |
| Hypertriglyceridemia | 233 | 1.26 | 0.896 | (0.761, 1.055) | 0.188 | 69 | 1.34 | 0.846 | (0.646, 1.109) | 0.227 |
| No low HDL-C level | 621 | 1.44 | Reference | | | 344 | 1.67 | Reference | | |
| Low HDL-C level | 91 | 1.31 | 1.036 | (0.824, 1.302) | 0.765 | 32 | 1.61 | 0.907 | (0.622, 1.322) | 0.610 |
|  |  |  |  |  |  |  |  |  |  |  |
| **Major osteoporotic fracture** |  |  |  |  |  |  |  |  |  |  |
| ***Number of MetS-related traits***^†^ |  |  |  |  |  |  |  |  |  |  |
| 1 (T2D only) | NA | | | | | 87 | 1.64 | Reference | | |
| 2 | 125 | 2.45 | Reference | | | 326 | 3.38 | 1.564 | (1.227, 1.994) | <0.001 |
| 3 | 649 | 3.28 | 0.947 | (0.779, 1.150) | 0.582 | 249 | 4.40 | 1.712 | (1.327, 2.210) | <0.001 |
| 4 | 663 | 4.01 | 1.072 | (0.881, 1.305) | 0.485 | 65 | 4.12 | 1.559 | (1.119, 2.170) | 0.009 |
| 5 | 226 | 3.25 | 0.879 | (0.703, 1.100) | 0.260 | 11 | 4.30 | 1.399 | (0.740, 2.644) | 0.302 |
| 6 | 53 | 3.81 | 1.014 | (0.731, 1.405) | 0.935 | NA | | | | |
| ≥3 or ≥2^‡^ | 1,591 | 3.56 | 0.984 | (0.817, 1.186) | 0.867 | 651 | 3.80 | 1.610 | (1.276, 2.030) | <0.001 |
| Continuous | NA | | 0.998 | (0.947, 1.053) | 0.955 | NA | | 1.123 | (1.035, 1.218) | 0.006 |
| ***Individual MetS-related traits***^†^ |  |  |  |  |  |  |  |  |  |  |
| No albuminuria | 1,131 | 3.12 | Reference | | | 472 | 2.68 | Reference | | |
| Albuminuria | 585 | 4.34 | 1.115 | (1.005, 1.238) | 0.041 | 266 | 5.54 | 1.384 | (1.173, 1.633) | <0.001 |
| No hypertension | 218 | 2.35 | Reference | | | 150 | 1.96 | Reference | | |
| Hypertension | 1,498 | 3.70 | 0.987 | (0.850, 1.147) | 0.869 | 588 | 3.98 | 1.249 | (1.026, 1.520) | 0.026 |
| No hypertriglyceridemia | 1,144 | 3.65 | Reference | | | 590 | 3.41 | Reference | | |
| Hypertriglyceridemia | 572 | 3.11 | 0.921 | (0.830, 1.022) | 0.123 | 148 | 2.89 | 0.923 | (0.766, 1.113) | 0.402 |
| No low HDL-C level | 1,506 | 3.51 | Reference | | | 677 | 3.31 | Reference | | |
| Low HDL-C level | 210 | 3.04 | 0.983 | (0.846, 1.142) | 0.823 | 61 | 3.08 | 0.894 | (0.681, 1.174) | 0.421 |

Notes: HR = Hazard ratio; CI = Confidence interval; MetS = Metabolic syndrome; T2D = Type 2 diabetes; HbA1c = Glycated hemoglobin; HDL = High-density lipoprotein; NA = Not available.

*The models were adjusted by age, sex, smoking status, cardiovascular disease, hyperlipidemia, chronic kidney disease, chronic obstructive pulmonary disease, liver disease, rheumatoid arthritis and other inflammatory polyarthropathies, history of falls (as a proxy indicator for frailty), osteoporosis, hyperparathyroidism, dementia, diabetic retinopathy, severe hypoglycemia, use of antidiabetic medications and systemic glucocorticoids, and baseline HbA1c level.

† MetS-related traits include albuminuria and individual MetS traits (obesity, hypertension, low HDL-cholesterol, and hypertriglyceridemia).

‡ ≥2 MetS-related traits indicate the presence of at least one additional MetS-related trait beyond T2D, while ≥3 MetS-related traits indicate at least one additional MetS-related trait beyond T2D and obesity.

## Supplementary Table 18. Incidence rates and HRs for fractures associated with albuminuria and MetS traits present at baseline in obese and non-obese patients with T2D, excluding those with a diagnosis of osteoporosis or systemic glucocorticoid use.

| Outcome | **Obese (N = 92,993)** | | | | | **Non-obese (N = 66,533)** | | | | |
| --- | --- | --- | --- | --- | --- | --- | --- | --- | --- | --- |
|  | Event | Incidence rate (per 1,000 person-years) | Adjusted HR^*^ | 95% CI | P-value | Event | Incidence rate (per 1,000 person-years) | Adjusted HR^*^ | 95% CI | P-value |
| **Hip fracture** |  |  |  |  |  |  |  |  |  |  |
| ***Number of MetS-related traits***^†^ |  |  |  |  |  |  |  |  |  |  |
| 1 (T2D only) | NA | | | | | 60 | 0.78 | Reference | | |
| 2 | 23 | 0.50 | Reference | | | 342 | 2.18 | 1.784 | (1.313, 2.424) | <0.001 |
| 3 | 186 | 0.92 | 1.203 | (0.726, 1.994) | 0.473 | 374 | 3.71 | 2.238 | (1.641, 3.052) | <0.001 |
| 4 | 242 | 1.35 | 1.559 | (0.943, 2.577) | 0.083 | 122 | 3.95 | 2.178 | (1.536, 3.087) | <0.001 |
| 5 | 103 | 1.30 | 1.359 | (0.798, 2.317) | 0.259 | 23 | 4.21 | 1.934 | (1.148, 3.257) | 0.013 |
| 6 | 28 | 1.85 | 1.928 | (1.013, 3.669) | 0.046 | NA | | | | |
| ≥3 or ≥2^‡^ | 559 | 1.18 | 1.381 | (0.845, 2.257) | 0.198 | 861 | 2.93 | 1.981 | (1.472, 2.665) | <0.001 |
| Continuous | NA | | 1.127 | (1.020, 1.245) | 0.019 | NA | | 1.179 | (1.093, 1.273) | <0.001 |
| ***Individual MetS-related traits***^†^ |  |  |  |  |  |  |  |  |  |  |
| No albuminuria | 331 | 0.88 | Reference | | | 499 | 1.76 | Reference | | |
| Albuminuria | 251 | 1.72 | 1.382 | (1.147, 1.665) | <0.001 | 422 | 4.89 | 1.608 | (1.387, 1.863) | <0.001 |
| No hypertension | 40 | 0.45 | Reference | | | 116 | 1.03 | Reference | | |
| Hypertension | 542 | 1.26 | 1.329 | (0.916, 1.929) | 0.135 | 805 | 3.12 | 1.355 | (1.084, 1.693) | 0.008 |
| No hypertriglyceridemia | 368 | 1.17 | Reference | | | 681 | 2.46 | Reference | | |
| Hypertriglyceridemia | 214 | 1.04 | 0.897 | (0.740, 1.088) | 0.269 | 240 | 2.55 | 0.990 | (0.842, 1.164) | 0.903 |
| No low HDL-C level | 498 | 1.12 | Reference | | | 840 | 2.50 | Reference | | |
| Low HDL-C level | 84 | 1.12 | 1.129 | (0.871, 1.465) | 0.359 | 81 | 2.36 | 0.811 | (0.634, 1.037) | 0.094 |
|  |  |  |  |  |  |  |  |  |  |  |
| **Major osteoporotic fracture** |  |  |  |  |  |  |  |  |  |  |
| ***Number of MetS-related traits***^†^ |  |  |  |  |  |  |  |  |  |  |
| 1 (T2D only) | NA | | | | | 171 | 2.24 | Reference | | |
| 2 | 99 | 2.16 | Reference | | | 684 | 4.39 | 1.409 | (1.175, 1.689) | <0.001 |
| 3 | 552 | 2.76 | 0.918 | (0.730, 1.154) | 0.463 | 626 | 6.27 | 1.616 | (1.340, 1.949) | <0.001 |
| 4 | 590 | 3.32 | 1.018 | (0.809, 1.281) | 0.880 | 193 | 6.31 | 1.423 | (1.131, 1.790) | 0.003 |
| 5 | 240 | 3.05 | 0.926 | (0.718, 1.194) | 0.552 | 41 | 7.59 | 1.507 | (1.040, 2.183) | 0.030 |
| 6 | 53 | 3.51 | 1.004 | (0.694, 1.452) | 0.985 | NA | | | | |
| ≥3 or ≥2^‡^ | 1,435 | 3.04 | 0.958 | (0.770, 1.194) | 0.705 | 1,544 | 5.30 | 1.481 | (1.244, 1.762) | <0.001 |
| Continuous | NA | | 1.012 | (0.954, 1.074) | 0.690 | NA | | 1.102 | (1.042, 1.165) | <0.001 |
| ***Individual MetS-related traits***^†^ |  |  |  |  |  |  |  |  |  |  |
| No albuminuria | 999 | 2.68 | Reference | | | 1,074 | 3.80 | Reference | | |
| Albuminuria | 535 | 3.69 | 1.140 | (1.015, 1.280) | 0.027 | 641 | 7.50 | 1.283 | (1.147, 1.434) | <0.001 |
| No hypertension | 176 | 1.97 | Reference | | | 285 | 2.55 | Reference | | |
| Hypertension | 1,358 | 3.17 | 0.983 | (0.826, 1.170) | 0.846 | 1,430 | 5.59 | 1.267 | (1.096, 1.465) | 0.001 |
| No hypertriglyceridemia | 966 | 3.08 | Reference | | | 1,259 | 4.59 | Reference | | |
| Hypertriglyceridemia | 568 | 2.78 | 0.925 | (0.825, 1.037) | 0.181 | 456 | 4.89 | 1.011 | (0.899, 1.137) | 0.855 |
| No low HDL-C level | 1,331 | 3.01 | Reference | | | 1,563 | 4.68 | Reference | | |
| Low HDL-C level | 203 | 2.71 | 1.013 | (0.861, 1.191) | 0.878 | 152 | 4.47 | 0.823 | (0.686, 0.989) | 0.037 |

Notes: HR = Hazard ratio; CI = Confidence interval; MetS = Metabolic syndrome; T2D = Type 2 diabetes; HbA1c = Glycated hemoglobin; HDL = High-density lipoprotein; NA = Not available.

*The models were adjusted by age, sex, smoking status, cardiovascular disease, hyperlipidemia, chronic kidney disease, chronic obstructive pulmonary disease, liver disease, rheumatoid arthritis and other inflammatory polyarthropathies, history of falls (as a proxy indicator for frailty), osteoporosis, hyperparathyroidism, dementia, diabetic retinopathy, severe hypoglycemia, use of antidiabetic medications and systemic glucocorticoids, and baseline HbA1c level.

† MetS-related traits include albuminuria and individual MetS traits (obesity, hypertension, low HDL-cholesterol, and hypertriglyceridemia).

‡ ≥2 MetS-related traits indicate the presence of at least one additional MetS-related trait beyond T2D, while ≥3 MetS-related traits indicate at least one additional MetS-related trait beyond T2D and obesity.

## Supplementary Table 19. Incidence rates and HRs for fractures associated with albuminuria and MetS traits present at baseline in obese and non-obese patients with T2D, overall and stratified by age, excluding fracture events accompanied by diagnostic codes for motor vehicle accidents or accidental falls from height.

| Outcome | **Obese (N = 96,251)** | | | | | **Non-obese (N = 69,038)** | | | | |
| --- | --- | --- | --- | --- | --- | --- | --- | --- | --- | --- |
|  | Event | Incidence rate (per 1,000 person-years) | Adjusted HR^*^ | 95% CI | P-value | Event | Incidence rate (per 1,000 person-years) | Adjusted HR^*^ | 95% CI | P-value |
| **Hip fracture** |  |  |  |  |  |  |  |  |  |  |
| ***Number of MetS-related traits***^†^ |  |  |  |  |  |  |  |  |  |  |
| 1 (T2D only) | NA | | | | | 63 | 0.80 | Reference | | |
| 2 | 24 | 0.51 | Reference | | | 368 | 2.27 | 1.800 | (1.337, 2.423) | <0.001 |
| 3 | 196 | 0.94 | 1.159 | (0.709, 1.894) | 0.557 | 384 | 3.67 | 2.147 | (1.588, 2.903) | <0.001 |
| 4 | 262 | 1.41 | 1.494 | (0.916, 2.435) | 0.108 | 128 | 4.01 | 2.191 | (1.561, 3.074) | <0.001 |
| 5 | 107 | 1.31 | 1.275 | (0.758, 2.144) | 0.359 | 23 | 4.08 | 1.881 | (1.122, 3.151) | 0.016 |
| 6 | 28 | 1.77 | 1.712 | (0.909, 3.224) | 0.096 | NA | | | | |
| ≥3 or ≥2^‡^ | 593 | 1.21 | 1.317 | (0.817, 2.125) | 0.258 | 903 | 2.97 | 1.960 | (1.469, 2.614) | <0.001 |
| Continuous | NA | | 1.106 | (1.004, 1.220) | 0.042 | NA | | 1.171 | (1.087, 1.262) | <0.001 |
| ***Individual MetS-related traits***^†^ |  |  |  |  |  |  |  |  |  |  |
| No albuminuria | 351 | 0.91 | Reference | | | 531 | 1.82 | Reference | | |
| Albuminuria | 266 | 1.75 | 1.332 | (1.111, 1.596) | 0.002 | 435 | 4.84 | 1.540 | (1.334, 1.778) | <0.001 |
| No hypertension | 43 | 0.47 | Reference | | | 121 | 1.05 | Reference | | |
| Hypertension | 574 | 1.29 | 1.246 | (0.872, 1.781) | 0.228 | 845 | 3.16 | 1.356 | (1.090, 1.686) | 0.006 |
| No hypertriglyceridemia | 391 | 1.20 | Reference | | | 717 | 2.51 | Reference | | |
| Hypertriglyceridemia | 226 | 1.07 | 0.904 | (0.750, 1.091) | 0.292 | 249 | 2.57 | 1.012 | (0.864, 1.185) | 0.886 |
| No low HDL-C level | 530 | 1.15 | Reference | | | 883 | 2.55 | Reference | | |
| Low HDL-C level | 87 | 1.12 | 1.113 | (0.863, 1.435) | 0.410 | 83 | 2.35 | 0.797 | (0.625, 1.017) | 0.068 |
|  |  |  |  |  |  |  |  |  |  |  |
| **Major osteoporotic fracture** |  |  |  |  |  |  |  |  |  |  |
| ***Number of MetS-related traits***^†^ |  |  |  |  |  |  |  |  |  |  |
| 1 (T2D only) | NA | | | | | 179 | 2.30 | Reference | | |
| 2 | 100 | 2.14 | Reference | | | 718 | 4.47 | 1.398 | (1.170, 1.669) | <0.001 |
| 3 | 576 | 2.79 | 0.924 | (0.736, 1.159) | 0.492 | 648 | 6.25 | 1.576 | (1.311, 1.893) | <0.001 |
| 4 | 620 | 3.37 | 1.017 | (0.810, 1.278) | 0.883 | 201 | 6.35 | 1.420 | (1.135, 1.777) | 0.002 |
| 5 | 250 | 3.07 | 0.924 | (0.719, 1.189) | 0.540 | 41 | 7.36 | 1.448 | (1.002, 2.094) | 0.049 |
| 6 | 56 | 3.56 | 0.998 | (0.694, 1.436) | 0.993 | NA | | | | |
| ≥3 or ≥2^‡^ | 1,502 | 3.08 | 0.960 | (0.772, 1.194) | 0.716 | 1,608 | 5.33 | 1.460 | (1.231, 1.731) | <0.001 |
| Continuous | NA | | 1.009 | (0.952, 1.070) | 0.752 | NA | | 1.096 | (1.038, 1.158) | 0.001 |
| ***Individual MetS-related traits***^†^ |  |  |  |  |  |  |  |  |  |  |
| No albuminuria | 1,039 | 2.71 | Reference | | | 1,118 | 3.85 | Reference | | |
| Albuminuria | 563 | 3.73 | 1.127 | (1.006, 1.263) | 0.039 | 669 | 7.51 | 1.281 | (1.149, 1.429) | <0.001 |
| No hypertension | 181 | 1.98 | Reference | | | 298 | 2.61 | Reference | | |
| Hypertension | 1,421 | 3.21 | 0.971 | (0.818, 1.152) | 0.733 | 1,489 | 5.62 | 1.241 | (1.077, 1.430) | 0.003 |
| No hypertriglyceridemia | 1,008 | 3.12 | Reference | | | 1,319 | 4.65 | Reference | | |
| Hypertriglyceridemia | 594 | 2.82 | 0.933 | (0.835, 1.044) | 0.228 | 468 | 4.88 | 1.013 | (0.903, 1.138) | 0.824 |
| No low HDL-C level | 1,390 | 3.05 | Reference | | | 1,632 | 4.74 | Reference | | |
| Low HDL-C level | 212 | 2.74 | 1.009 | (0.860, 1.182) | 0.916 | 155 | 4.43 | 0.811 | (0.676, 0.972) | 0.023 |

| *Subgroup: patients aged <50 years* | | | | | | | | | | |
| --- | --- | --- | --- | --- | --- | --- | --- | --- | --- | --- |
| Outcome | **Obese (N =19,114)** | | | | | **Non-obese (N =8,062)** | | | | |
|  | Event | Incidence rate (per 1,000 person-years) | Adjusted HR^*^ | 95% CI | P-value | Event | Incidence rate (per 1,000 person-years) | Adjusted HR^*^ | 95% CI | P-value |
| **Major osteoporotic fracture** |  |  |  |  |  |  |  |  |  |  |
| ***Number of MetS-related traits***^†^ |  |  |  |  |  |  |  |  |  |  |
| 1 (T2D only) | NA | | | | | 19 | 1.03 | Reference | | |
| 2 | 8 | 0.48 | Reference | | | 12 | 0.72 | 0.641 | (0.293, 1.405) | 0.267 |
| 3 | 23 | 0.62 | 1.250 | (0.554, 2.822) | 0.591 | 24 | 2.25 | 2.317 | (1.218, 4.408) | 0.010 |
| 4 | 41 | 1.16 | 2.088 | (0.960, 4.540) | 0.063 | 4 | 1.18 | 1.229 | (0.406, 3.720) | 0.716 |
| 5 | 19 | 0.99 | 1.838 | (0.785, 4.307) | 0.161 | 1 | 1.35 | 1.367 | (0.178, 10.482) | 0.764 |
| 6 | 2 | 0.42 | 0.380 | (0.047, 3.072) | 0.364 | NA | | | | |
| ≥3 or ≥2^‡^ | 85 | 0.88 | 1.620 | (0.774, 3.392) | 0.200 | 41 | 1.30 | 1.255 | (0.699, 2.255) | 0.447 |
| Continuous | NA | | 1.111 | (0.906, 1.363) | 0.310 | NA | | 1.281 | (0.993, 1.653) | 0.056 |
| ***Individual MetS-related traits***^†^ |  |  |  |  |  |  |  |  |  |  |
| No albuminuria | 53 | 0.65 | Reference | | | 44 | 1.09 | Reference | | |
| Albuminuria | 40 | 1.26 | 1.567 | (0.981, 2.505) | 0.060 | 16 | 1.65 | 1.509 | (0.785, 2.903) | 0.217 |
| No hypertension | 26 | 0.72 | Reference | | | 30 | 1.06 | Reference | | |
| Hypertension | 67 | 0.87 | 1.026 | (0.615, 1.712) | 0.920 | 30 | 1.38 | 1.220 | (0.679, 2.190) | 0.506 |
| No hypertriglyceridemia | 48 | 0.78 | Reference | | | 41 | 1.12 | Reference | | |
| Hypertriglyceridemia | 45 | 0.87 | 1.058 | (0.679, 1.647) | 0.805 | 19 | 1.42 | 1.121 | (0.621, 2.023) | 0.705 |
| No low HDL-C level | 75 | 0.84 | Reference | | | 49 | 1.12 | Reference | | |
| Low HDL-C level | 18 | 0.75 | 0.839 | (0.484, 1.455) | 0.533 | 11 | 1.73 | 1.379 | (0.674, 2.824) | 0.379 |

| *Subgroup: patients aged* ≥*50 years* | | | | | | | | | | |
| --- | --- | --- | --- | --- | --- | --- | --- | --- | --- | --- |
| Outcome | **Obese (N =77,137)** | | | | | **Non-obese (N =60,976)** | | | | |
|  | Event | Incidence rate (per 1,000 person-years) | Adjusted HR^*^ | 95% CI | P-value | Event | Incidence rate (per 1,000 person-years) | Adjusted HR^*^ | 95% CI | P-value |
| **Hip fracture** |  |  |  |  |  |  |  |  |  |  |
| ***Number of MetS-related traits***^†^ |  |  |  |  |  |  |  |  |  |  |
| 1 (T2D only) | NA | | | | | 60 | 1.00 | Reference | | |
| 2 | 24 | 0.79 | Reference | | | 367 | 2.53 | 1.811 | (1.339, 2.449) | <0.001 |
| 3 | 196 | 1.15 | 1.109 | (0.678, 1.811) | 0.681 | 378 | 4.03 | 2.122 | (1.561, 2.883) | <0.001 |
| 4 | 256 | 1.71 | 1.394 | (0.855, 2.273) | 0.183 | 127 | 4.45 | 2.185 | (1.550, 3.080) | <0.001 |
| 5 | 103 | 1.65 | 1.164 | (0.690, 1.961) | 0.569 | 23 | 4.70 | 1.892 | (1.126, 3.179) | 0.016 |
| 6 | 28 | 2.52 | 1.655 | (0.878, 3.118) | 0.119 | NA | | | | |
| ≥3 or ≥2^‡^ | 583 | 1.48 | 1.238 | (0.768, 1.997) | 0.381 | 895 | 3.29 | 1.957 | (1.460, 2.623) | <0.001 |
| Continuous | NA | | 1.090 | (0.987, 1.203) | 0.089 | NA | | 1.165 | (1.080, 1.257) | <0.001 |
| ***Individual MetS-related traits***^†^ |  |  |  |  |  |  |  |  |  |  |
| No albuminuria | 349 | 1.15 | Reference | | | 524 | 2.08 | Reference | | |
| Albuminuria | 258 | 2.15 | 1.294 | (1.077, 1.554) | 0.006 | 431 | 5.38 | 1.533 | (1.327, 1.771) | <0.001 |
| No hypertension | 42 | 0.75 | Reference | | | 115 | 1.33 | Reference | | |
| Hypertension | 565 | 1.54 | 1.198 | (0.835, 1.718) | 0.328 | 840 | 3.42 | 1.371 | (1.099, 1.711) | 0.005 |
| No hypertriglyceridemia | 386 | 1.46 | Reference | | | 710 | 2.86 | Reference | | |
| Hypertriglyceridemia | 221 | 1.38 | 0.895 | (0.741, 1.082) | 0.254 | 245 | 2.94 | 1.007 | (0.859, 1.181) | 0.930 |
| No low HDL-C level | 522 | 1.41 | Reference | | | 875 | 2.89 | Reference | | |
| Low HDL-C level | 85 | 1.58 | 1.123 | (0.868, 1.453) | 0.376 | 80 | 2.77 | 0.776 | (0.606, 0.995) | 0.045 |
|  |  |  |  |  |  |  |  |  |  |  |
| **Major osteoporotic fracture** |  |  |  |  |  |  |  |  |  |  |
| ***Number of MetS-related traits***^†^ |  |  |  |  |  |  |  |  |  |  |
| 1 (T2D only) | NA | | | | | 157 | 2.65 | Reference | | |
| 2 | 91 | 3.02 | Reference | | | 701 | 4.87 | 1.461 | (1.212, 1.762) | <0.001 |
| 3 | 543 | 3.21 | 0.864 | (0.681, 1.096) | 0.229 | 620 | 6.67 | 1.592 | (1.312, 1.932) | <0.001 |
| 4 | 567 | 3.82 | 0.926 | (0.728, 1.176) | 0.527 | 196 | 6.93 | 1.462 | (1.158, 1.846) | 0.001 |
| 5 | 228 | 3.67 | 0.842 | (0.646, 1.097) | 0.203 | 38 | 7.86 | 1.449 | (0.989, 2.123) | 0.057 |
| 6 | 53 | 4.81 | 0.992 | (0.681, 1.444) | 0.967 | NA | | | | |
| ≥3 or ≥2^‡^ | 1,391 | 3.56 | 0.887 | (0.705, 1.115) | 0.304 | 1,555 | 5.76 | 1.505 | (1.256, 1.802) | <0.001 |
| Continuous | NA | | 0.997 | (0.937, 1.061) | 0.933 | NA | | 1.089 | (1.029, 1.152) | 0.003 |
| ***Individual MetS-related traits***^†^ |  |  |  |  |  |  |  |  |  |  |
| No albuminuria | 967 | 3.20 | Reference | | | 1,065 | 4.26 | Reference | | |
| Albuminuria | 515 | 4.33 | 1.108 | (0.984, 1.247) | 0.091 | 647 | 8.15 | 1.276 | (1.141, 1.425) | <0.001 |
| No hypertension | 154 | 2.77 | Reference | | | 265 | 3.08 | Reference | | |
| Hypertension | 1,328 | 3.63 | 0.930 | (0.774, 1.117) | 0.436 | 1,447 | 5.94 | 1.246 | (1.076, 1.444) | 0.003 |
| No hypertriglyceridemia | 944 | 3.60 | Reference | | | 1,267 | 5.13 | Reference | | |
| Hypertriglyceridemia | 538 | 3.39 | 0.914 | (0.813, 1.027) | 0.131 | 445 | 5.39 | 1.006 | (0.893, 1.133) | 0.921 |
| No low HDL-C level | 1,290 | 3.51 | Reference | | | 1,570 | 5.22 | Reference | | |
| Low HDL-C level | 192 | 3.59 | 1.044 | (0.883, 1.233) | 0.616 | 142 | 4.96 | 0.788 | (0.653, 0.952) | 0.013 |

Notes: HR = Hazard ratio; CI = Confidence interval; MetS = Metabolic syndrome; T2D = Type 2 diabetes; HbA1c = Glycated hemoglobin; HDL = High-density lipoprotein; NA = Not available. Results for hip fracture were omitted because the number of events in each MetS trait category did not exceed 10 among younger patients.

*The models were adjusted by age, sex, smoking status, cardiovascular disease, hyperlipidemia, chronic kidney disease, chronic obstructive pulmonary disease, liver disease, rheumatoid arthritis and other inflammatory polyarthropathies, history of falls (as a proxy indicator for frailty), osteoporosis, hyperparathyroidism, dementia, diabetic retinopathy, severe hypoglycemia, use of antidiabetic medications and systemic glucocorticoids, and baseline HbA1c level.

† MetS-related traits include albuminuria and individual MetS traits (obesity, hypertension, low HDL-cholesterol, and hypertriglyceridemia).

‡ ≥2 MetS-related traits indicate the presence of at least one additional MetS-related trait beyond T2D, while ≥3 MetS-related traits indicate at least one additional MetS-related trait beyond T2D and obesity.

## Supplementary Table 20. Incidence rates and HRs for fractures associated with albuminuria and MetS traits present at baseline in obese and non-obese patients with T2D, excluding the COVID-19 pandemic period.

| Outcome | **Obese (N =96,251)** | | | | | **Non-obese (N =69,038)** | | | | |
| --- | --- | --- | --- | --- | --- | --- | --- | --- | --- | --- |
|  | Event | Incidence rate (per 1,000 person-years) | Adjusted HR^*^ | 95% CI | P-value | Event | Incidence rate (per 1,000 person-years) | Adjusted HR^*^ | 95% CI | P-value |
| **Hip fracture** |  |  |  |  |  |  |  |  |  |  |
| ***Number of MetS-related traits***^†^ |  |  |  |  |  |  |  |  |  |  |
| 1 (T2D only) | NA | | | | | 55 | 0.85 | Reference | | |
| 2 | 19 | 0.49 | Reference | | | 312 | 2.31 | 1.750 | (1.270, 2.410) | <0.001 |
| 3 | 160 | 0.93 | 1.326 | (0.747, 2.354) | 0.335 | 326 | 3.71 | 2.033 | (1.468, 2.815) | <0.001 |
| 4 | 220 | 1.43 | 1.738 | (0.982, 3.077) | 0.058 | 110 | 4.07 | 2.111 | (1.464, 3.045) | <0.001 |
| 5 | 98 | 1.43 | 1.624 | (0.892, 2.957) | 0.113 | 21 | 4.35 | 1.925 | (1.114, 3.327) | 0.019 |
| 6 | 24 | 1.82 | 2.074 | (1.013, 4.244) | 0.046 | NA | | | | |
| ≥3 or ≥2^‡^ | 502 | 1.23 | 1.547 | (0.884, 2.707) | 0.127 | 769 | 3.02 | 1.887 | (1.384, 2.573) | <0.001 |
| Continuous | NA | | 1.153 | (1.037, 1.283) | 0.008 | NA | | 1.165 | (1.073, 1.264) | <0.001 |
| ***Individual MetS-related traits***^†^ |  |  |  |  |  |  |  |  |  |  |
| No albuminuria | 298 | 0.93 | Reference | | | 451 | 1.85 | Reference | | |
| Albuminuria | 223 | 1.76 | 1.284 | (1.052, 1.568) | 0.014 | 373 | 4.91 | 1.519 | (1.298, 1.776) | <0.001 |
| No hypertension | 35 | 0.46 | Reference | | | 103 | 1.08 | Reference | | |
| Hypertension | 486 | 1.31 | 1.386 | (0.925, 2.077) | 0.114 | 721 | 3.22 | 1.355 | (1.068, 1.719) | 0.012 |
| No hypertriglyceridemia | 317 | 1.17 | Reference | | | 609 | 2.55 | Reference | | |
| Hypertriglyceridemia | 204 | 1.16 | 0.992 | (0.810, 1.216) | 0.940 | 215 | 2.65 | 1.032 | (0.869, 1.225) | 0.721 |
| No low HDL-C level | 444 | 1.16 | Reference | | | 755 | 2.61 | Reference | | |
| Low HDL-C level | 77 | 1.18 | 1.172 | (0.893, 1.537) | 0.253 | 69 | 2.31 | 0.762 | (0.582, 0.997) | 0.048 |
|  |  |  |  |  |  |  |  |  |  |  |
| **Major osteoporotic fracture** |  |  |  |  |  |  |  |  |  |  |
| ***Number of MetS-related traits***^†^ |  |  |  |  |  |  |  |  |  |  |
| 1 (T2D only) | NA | | | | | 154 | 2.38 | Reference | | |
| 2 | 83 | 2.14 | Reference | | | 591 | 4.41 | 1.321 | (1.090, 1.603) | 0.005 |
| 3 | 480 | 2.81 | 0.950 | (0.739, 1.222) | 0.692 | 564 | 6.47 | 1.549 | (1.270, 1.889) | <0.001 |
| 4 | 508 | 3.31 | 1.012 | (0.786, 1.304) | 0.926 | 173 | 6.45 | 1.368 | (1.072, 1.745) | 0.012 |
| 5 | 213 | 3.13 | 0.950 | (0.719, 1.254) | 0.716 | 35 | 7.32 | 1.405 | (0.944, 2.092) | 0.094 |
| 6 | 47 | 3.58 | 1.020 | (0.685, 1.518) | 0.923 | NA | | | | |
| ≥3 or ≥2^‡^ | 1,248 | 3.08 | 0.975 | (0.766, 1.242) | 0.838 | 1,363 | 5.39 | 1.403 | (1.167, 1.687) | <0.001 |
| Continuous | NA | | 1.008 | (0.945, 1.075) | 0.817 | NA | | 1.098 | (1.034, 1.166) | 0.002 |
| ***Individual MetS-related traits***^†^ |  |  |  |  |  |  |  |  |  |  |
| No albuminuria | 869 | 2.73 | Reference | | | 941 | 3.89 | Reference | | |
| Albuminuria | 462 | 3.66 | 1.084 | (0.956, 1.230) | 0.210 | 576 | 7.64 | 1.285 | (1.141, 1.447) | <0.001 |
| No hypertension | 153 | 2.01 | Reference | | | 250 | 2.63 | Reference | | |
| Hypertension | 1,178 | 3.20 | 0.972 | (0.805, 1.173) | 0.764 | 1,267 | 5.70 | 1.238 | (1.060, 1.446) | 0.007 |
| No hypertriglyceridemia | 830 | 3.09 | Reference | | | 1,112 | 4.69 | Reference | | |
| Hypertriglyceridemia | 501 | 2.86 | 0.948 | (0.838, 1.072) | 0.394 | 405 | 5.03 | 1.039 | (0.916, 1.178) | 0.555 |
| No low HDL-C level | 1,149 | 3.03 | Reference | | | 1,387 | 4.82 | Reference | | |
| Low HDL-C level | 182 | 2.81 | 1.033 | (0.869, 1.227) | 0.715 | 130 | 4.38 | 0.781 | (0.640, 0.953) | 0.015 |

Notes: HR = Hazard ratio; CI = Confidence interval; MetS = Metabolic syndrome; T2D = Type 2 diabetes; HbA1c = Glycated hemoglobin; HDL = High-density lipoprotein; NA = Not available.

* The models were adjusted by age, sex, smoking status, cardiovascular disease, hyperlipidemia, chronic kidney disease, chronic obstructive pulmonary disease, liver disease, rheumatoid arthritis and other inflammatory polyarthropathies, history of falls (as a proxy indicator for frailty), osteoporosis, hyperparathyroidism, dementia, diabetic retinopathy, severe hypoglycemia, use of antidiabetic medications and systemic glucocorticoids, and baseline HbA1c level.

† MetS-related traits include albuminuria and individual MetS traits (obesity, hypertension, low HDL-cholesterol, and hypertriglyceridemia).

‡ ≥2 MetS-related traits indicate the presence of at least one additional MetS-related trait beyond T2D, while ≥3 MetS-related traits indicate at least one additional MetS-related trait beyond T2D and obesity.

## Supplementary Table 21. Incidence rates and HRs for fractures associated with albuminuria and MetS traits present at baseline in obese and non-obese patients with T2D, with additional adjustment for diabetes duration.

| Outcome | **Obese (N =96,251)** | | | | | **Non-obese (N =69,038)** | | | | |
| --- | --- | --- | --- | --- | --- | --- | --- | --- | --- | --- |
|  | Event | Incidence rate (per 1,000 person-years) | Adjusted HR^*^ | 95% CI | P-value | Event | Incidence rate (per 1,000 person-years) | Adjusted HR^*^ | 95% CI | P-value |
| **Hip fracture** |  |  |  |  |  |  |  |  |  |  |
| ***Number of MetS-related traits***^†^ |  |  |  |  |  |  |  |  |  |  |
| 1 (T2D only) | NA | | | | | 63 | 0.80 | Reference | | |
| 2 | 24 | 0.51 | Reference | | | 368 | 2.27 | 1.799 | (1.336, 2.421) | <0.001 |
| 3 | 196 | 0.94 | 1.159 | (0.709, 1.894) | 0.557 | 384 | 3.67 | 2.141 | (1.584, 2.896) | <0.001 |
| 4 | 262 | 1.41 | 1.494 | (0.916, 2.436) | 0.108 | 128 | 4.01 | 2.190 | (1.560, 3.073) | <0.001 |
| 5 | 107 | 1.31 | 1.275 | (0.758, 2.145) | 0.359 | 23 | 4.08 | 1.877 | (1.120, 3.145) | 0.017 |
| 6 | 28 | 1.77 | 1.712 | (0.909, 3.224) | 0.096 | NA | | | | |
| ≥3 or ≥2^‡^ | 593 | 1.21 | 1.317 | (0.817, 2.124) | 0.259 | 903 | 2.97 | 1.957 | (1.467, 2.610) | <0.001 |
| Continuous | NA | | 1.106 | (1.003, 1.220) | 0.042 | NA | | 1.170 | (1.086, 1.261) | <0.001 |
| ***Individual MetS-related traits***^†^ |  |  |  |  |  |  |  |  |  |  |
| No albuminuria | 351 | 0.91 | Reference | | | 531 | 1.82 | Reference | | |
| Albuminuria | 266 | 1.75 | 1.333 | (1.112, 1.598) | 0.002 | 435 | 4.84 | 1.536 | (1.330, 1.774) | <0.001 |
| No hypertension | 43 | 0.47 | Reference | | | 121 | 1.05 | Reference | | |
| Hypertension | 574 | 1.29 | 1.246 | (0.872, 1.782) | 0.227 | 845 | 3.16 | 1.354 | (1.089, 1.683) | 0.006 |
| No hypertriglyceridemia | 391 | 1.20 | Reference | | | 717 | 2.51 | Reference | | |
| Hypertriglyceridemia | 226 | 1.07 | 0.904 | (0.749, 1.090) | 0.291 | 249 | 2.57 | 1.013 | (0.865, 1.187) | 0.870 |
| No low HDL-C level | 530 | 1.15 | Reference | | | 883 | 2.55 | Reference | | |
| Low HDL-C level | 87 | 1.12 | 1.113 | (0.863, 1.435) | 0.411 | 83 | 2.35 | 0.798 | (0.625, 1.018) | 0.069 |
| ***Other covariates at baseline*** |  |  |  |  |  |  |  |  |  |  |
| Time since T2D diagnosis^‡^ | 617 | 1.15 | 0.998 | (0.977, 1.020) | 0.875 | 966 | 2.53 | 1.004 | (0.992, 1.016) | 0.531 |
|  |  |  |  |  |  |  |  |  |  |  |
| **Major osteoporotic fracture** |  |  |  |  |  |  |  |  |  |  |
| ***Number of MetS-related traits***^†^ |  |  |  |  |  |  |  |  |  |  |
| 1 (T2D only) | NA | | | | | 180 | 2.31 | Reference | | |
| 2 | 100 | 2.14 | Reference | | | 718 | 4.47 | 1.408 | (1.178, 1.683) | <0.001 |
| 3 | 576 | 2.79 | 0.920 | (0.732, 1.156) | 0.474 | 649 | 6.26 | 1.589 | (1.321, 1.911) | <0.001 |
| 4 | 622 | 3.38 | 1.018 | (0.809, 1.281) | 0.879 | 201 | 6.35 | 1.437 | (1.148, 1.800) | 0.002 |
| 5 | 250 | 3.07 | 0.924 | (0.717, 1.190) | 0.539 | 41 | 7.36 | 1.431 | (0.985, 2.078) | 0.060 |
| 6 | 56 | 3.56 | 0.989 | (0.685, 1.426) | 0.951 | NA | | | | |
| ≥3 or ≥2^‡^ | 1,504 | 3.09 | 0.959 | (0.770, 1.194) | 0.706 | 1,609 | 5.34 | 1.471 | (1.240, 1.746) | <0.001 |
| Continuous | NA | | 1.010 | (0.952, 1.071) | 0.744 | NA | | 1.097 | (1.039, 1.160) | <0.001 |
| ***Individual MetS-related traits***^†^ |  |  |  |  |  |  |  |  |  |  |
| No albuminuria | 1,040 | 2.71 | Reference | | | 1,120 | 3.86 | Reference | | |
| Albuminuria | 564 | 3.74 | 1.132 | (1.010, 1.270) | 0.034 | 669 | 7.51 | 1.275 | (1.143, 1.423) | <0.001 |
| No hypertension | 181 | 1.98 | Reference | | | 299 | 2.62 | Reference | | |
| Hypertension | 1,423 | 3.22 | 0.963 | (0.811, 1.144) | 0.669 | 1,490 | 5.62 | 1.241 | (1.077, 1.431) | 0.003 |
| No hypertriglyceridemia | 1,009 | 3.12 | Reference | | | 1,320 | 4.66 | Reference | | |
| Hypertriglyceridemia | 595 | 2.83 | 0.932 | (0.833, 1.044) | 0.222 | 469 | 4.89 | 1.021 | (0.909, 1.147) | 0.724 |
| No low HDL-C level | 1,392 | 3.05 | Reference | | | 1,634 | 4.75 | Reference | | |
| Low HDL-C level | 212 | 2.74 | 1.012 | (0.862, 1.187) | 0.885 | 155 | 4.43 | 0.812 | (0.677, 0.974) | 0.025 |
| ***Other covariates at baseline*** |  |  |  |  |  |  |  |  |  |  |
| Time since T2D diagnosis^§^ | 1,604 | 3.00 | 0.998 | (0.984, 1.012) | 0.763 | 1,789 | 4.72 | 1.009 | (0.999, 1.018) | 0.079 |

Notes: HR = Hazard ratio; CI = Confidence interval; MetS = Metabolic syndrome; T2D = Type 2 diabetes; HbA1c = Glycated hemoglobin; HDL = High-density lipoprotein; NA = Not available.

* The models were adjusted by age, sex, time since T2D diagnosis, smoking status, cardiovascular disease, hyperlipidemia, chronic kidney disease, chronic obstructive pulmonary disease, liver disease, rheumatoid arthritis and other inflammatory polyarthropathies, history of falls (as a proxy indicator for frailty), osteoporosis, hyperparathyroidism, dementia, diabetic retinopathy, severe hypoglycemia, use of antidiabetic medications and systemic glucocorticoids, and baseline HbA1c level.

† MetS-related traits include albuminuria and individual MetS traits (obesity, hypertension, low HDL-cholesterol, and hypertriglyceridemia).

‡ ≥2 MetS-related traits indicate the presence of at least one additional MetS-related trait beyond T2D, while ≥3 MetS-related traits indicate at least one additional MetS-related trait beyond T2D and obesity.

§ The hazard ratio was calculated from the model in which time since T2D diagnosis was additionally included as a covariate when MetS-related traits were treated as individual components, and represents the change in risk per one-year increase in duration.

## Supplementary Table 22. Incidence rates and HRs for fractures associated with albuminuria and MetS traits present at baseline in obese and non-obese patients with T2D, with additional adjustment for neuropathy and the use of calcium and vitamin D supplements.

| Outcome | **Obese (N =96,251)** | | | | | **Non-obese (N =69,038)** | | | | |
| --- | --- | --- | --- | --- | --- | --- | --- | --- | --- | --- |
|  | Event | Incidence rate (per 1,000 person-years) | Adjusted HR^*^ | 95% CI | P-value | Event | Incidence rate (per 1,000 person-years) | Adjusted HR^*^ | 95% CI | P-value |
| **Hip fracture** |  |  |  |  |  |  |  |  |  |  |
| ***Number of MetS-related traits***^†^ | |  |  |  |  |  |  |  |  |  |
| 1 (T2D only) | NA | | | | | 63 | 0.80 | Reference | | |
| 2 | 24 | 0.51 | Reference | | | 368 | 2.27 | 1.799 | (1.336, 2.421) | <0.001 |
| 3 | 196 | 0.94 | 1.158 | (0.708, 1.893) | 0.558 | 384 | 3.67 | 2.146 | (1.587, 2.902) | <0.001 |
| 4 | 262 | 1.41 | 1.492 | (0.915, 2.432) | 0.109 | 128 | 4.01 | 2.192 | (1.562, 3.077) | <0.001 |
| 5 | 107 | 1.31 | 1.270 | (0.755, 2.137) | 0.367 | 23 | 4.08 | 1.883 | (1.124, 3.155) | 0.016 |
| 6 | 28 | 1.77 | 1.705 | (0.905, 3.212) | 0.099 | NA | | | | |
| ≥3 or ≥2^‡^ | 593 | 1.21 | 1.315 | (0.815, 2.121) | 0.261 | 903 | 2.97 | 1.959 | (1.469, 2.613) | <0.001 |
| Continuous | NA | | 1.103 | (1.001, 1.216) | 0.048 | NA | | 1.172 | (1.087, 1.263) | <0.001 |
| ***Individual MetS-related traits***^†^ | |  |  |  |  |  |  |  |  |  |
| No albuminuria | 351 | 0.91 | Reference | | | 531 | 1.82 | Reference | | |
| Albuminuria | 266 | 1.75 | 1.328 | (1.108, 1.592) | 0.002 | 435 | 4.84 | 1.540 | (1.334, 1.778) | <0.001 |
| No hypertension | 43 | 0.47 | Reference | | | 121 | 1.05 | Reference | | |
| Hypertension | 574 | 1.29 | 1.240 | (0.867, 1.772) | 0.239 | 845 | 3.16 | 1.354 | (1.089, 1.684) | 0.006 |
| No hypertriglyceridemia | 391 | 1.20 | Reference | | | 717 | 2.51 | Reference | | |
| Hypertriglyceridemia | 226 | 1.07 | 0.903 | (0.749, 1.089) | 0.286 | 249 | 2.57 | 1.012 | (0.864, 1.186) | 0.878 |
| No low HDL-C level | 530 | 1.15 | Reference | | | 883 | 2.55 | Reference | | |
| Low HDL-C level | 87 | 1.12 | 1.115 | (0.865, 1.438) | 0.401 | 83 | 2.35 | 0.798 | (0.625, 1.018) | 0.069 |
|  |  |  |  |  |  |  |  |  |  |  |
| **Major osteoporotic fracture** |  |  |  |  |  |  |  |  |  |  |
| ***Number of MetS-related traits***^†^ | |  |  |  |  |  |  |  |  |  |
| 1 (T2D only) | NA | | | | | 180 | 2.31 | Reference | | |
| 2 | 100 | 2.14 | Reference | | | 718 | 4.47 | 1.408 | (1.178, 1.683) | <0.001 |
| 3 | 576 | 2.79 | 0.918 | (0.730, 1.153) | 0.461 | 649 | 6.26 | 1.593 | (1.325, 1.916) | <0.001 |
| 4 | 622 | 3.38 | 1.016 | (0.808, 1.278) | 0.893 | 201 | 6.35 | 1.439 | (1.149, 1.802) | 0.002 |
| 5 | 250 | 3.07 | 0.918 | (0.713, 1.183) | 0.508 | 41 | 7.36 | 1.433 | (0.987, 2.082) | 0.059 |
| 6 | 56 | 3.56 | 0.987 | (0.684, 1.425) | 0.945 | NA | | | | |
| ≥3 or ≥2^‡^ | 1,504 | 3.09 | 0.956 | (0.768, 1.190) | 0.688 | 1,609 | 5.34 | 1.473 | (1.241, 1.748) | <0.001 |
| Continuous | NA | | 1.009 | (0.951, 1.070) | 0.777 | NA | | 1.098 | (1.040, 1.161) | <0.001 |
| ***Individual MetS-related traits***^†^ | |  |  |  |  |  |  |  |  |  |
| No albuminuria | 1,040 | 2.71 | Reference | | | 1,120 | 3.86 | Reference | | |
| Albuminuria | 564 | 3.74 | 1.130 | (1.008, 1.268) | 0.036 | 669 | 7.51 | 1.281 | (1.148, 1.429) | <0.001 |
| No hypertension | 181 | 1.98 | Reference | | | 299 | 2.62 | Reference | | |
| Hypertension | 1,423 | 3.22 | 0.961 | (0.809, 1.142) | 0.650 | 1,490 | 5.62 | 1.243 | (1.078, 1.433) | 0.003 |
| No hypertriglyceridemia | 1,009 | 3.12 | Reference | | | 1,320 | 4.66 | Reference | | |
| Hypertriglyceridemia | 595 | 2.83 | 0.930 | (0.830, 1.041) | 0.205 | 469 | 4.89 | 1.018 | (0.907, 1.144) | 0.757 |
| No low HDL-C level | 1,392 | 3.05 | Reference | | | 1,634 | 4.75 | Reference | | |
| Low HDL-C level | 212 | 2.74 | 1.015 | (0.865, 1.191) | 0.855 | 155 | 4.43 | 0.812 | (0.677, 0.973) | 0.024 |

Notes: HR = Hazard ratio; CI = Confidence interval; MetS = Metabolic syndrome; T2D = Type 2 diabetes; HbA1c = Glycated hemoglobin; HDL = High-density lipoprotein; NA = Not available.

* The models were adjusted by age, sex, smoking status, cardiovascular disease, hyperlipidemia, chronic kidney disease, chronic obstructive pulmonary disease, liver disease, rheumatoid arthritis and other inflammatory polyarthropathies, history of falls (as a proxy indicator for frailty), osteoporosis, hyperparathyroidism, dementia, diabetic retinopathy, neuropathy, severe hypoglycemia, use of antidiabetic medications, systemic glucocorticoids, calcium and vitamin D supplements, and baseline HbA1c level.

† MetS-related traits include albuminuria and individual MetS traits (obesity, hypertension, low HDL-cholesterol, and hypertriglyceridemia).

‡ ≥2 MetS-related traits indicate the presence of at least one additional MetS-related trait beyond T2D, while ≥3 MetS-related traits indicate at least one additional MetS-related trait beyond T2D and obesity.

## Supplementary Table 23. Incidence rates and HRs for fractures associated with albuminuria and MetS traits present at baseline in obese and non-obese patients with T2D, using the definition of MetS proposed by the International Diabetes Federation.

| Outcome | **Obese (N =132,050)** | | | | | **Non-obese (N =57,107)** | | | | |
| --- | --- | --- | --- | --- | --- | --- | --- | --- | --- | --- |
|  | Event | Incidence rate (per 1,000 person-years) | Adjusted HR^*^ | 95% CI | P-value | Event | Incidence rate (per 1,000 person-years) | Adjusted HR^*^ | 95% CI | P-value |
| **Hip fracture** |  |  |  |  |  |  |  |  |  |  |
| ***Number of MetS-related traits***^†^ | |  |  |  |  |  |  |  |  |  |
| 1 (T2D only) | NA | | | | | 83 | 1.28 | Reference | | |
| 2 | 78 | 1.23 | Reference | | | 213 | 1.69 | 1.147 | (0.880, 1.495) | 0.310 |
| 3 | 327 | 1.50 | 1.039 | (0.807, 1.336) | 0.768 | 186 | 1.79 | 1.112 | (0.830, 1.489) | 0.476 |
| 4 | 450 | 1.56 | 1.022 | (0.792, 1.318) | 0.870 | 75 | 2.11 | 0.992 | (0.703, 1.401) | 0.965 |
| 5 | 271 | 1.70 | 0.890 | (0.679, 1.167) | 0.401 | NA | | | | |
| ≥3 or ≥2^‡^ | 1,048 | 1.58 | 1.007 | (0.791, 1.281) | 0.957 | 474 | 1.78 | 1.120 | (0.868, 1.445) | 0.383 |
| Continuous | NA | | 0.948 | (0.882, 1.019) | 0.151 | NA | | 0.984 | (0.888, 1.091) | 0.762 |
| ***Individual MetS-related traits***^†^ | |  |  |  |  |  |  |  |  |  |
| No hypertension | 352 | 1.16 | Reference | | | 205 | 1.15 | Reference | | |
| Hypertension | 774 | 1.82 | 1.025 | (0.895, 1.174) | 0.722 | 352 | 2.31 | 1.284 | (1.064, 1.549) | 0.009 |
| No hypertriglyceridemia | 401 | 1.77 | Reference | | | 265 | 1.87 | Reference | | |
| Hypertriglyceridemia | 725 | 1.44 | 0.800 | (0.677, 0.944) | 0.008 | 292 | 1.55 | 0.844 | (0.655, 1.086) | 0.188 |
| No low HDL-C level | 585 | 1.52 | Reference | | | 391 | 1.70 | Reference | | |
| Low HDL-C level | 541 | 1.57 | 0.999 | (0.883, 1.130) | 0.988 | 166 | 1.66 | 0.887 | (0.730, 1.078) | 0.228 |
|  |  |  |  |  |  |  |  |  |  |  |
| **Major osteoporotic fracture** |  |  |  |  |  |  |  |  |  |  |
| ***Number of MetS-related traits***^†^ | |  |  |  |  |  |  |  |  |  |
| 1 (T2D only) | NA | | | | | 180 | 2.80 | Reference | | |
| 2 | 193 | 3.06 | Reference | | | 409 | 3.27 | 1.077 | (0.897, 1.295) | 0.426 |
| 3 | 774 | 3.58 | 1.053 | (0.896, 1.237) | 0.530 | 368 | 3.55 | 1.080 | (0.882, 1.323) | 0.458 |
| 4 | 1,030 | 3.59 | 1.015 | (0.862, 1.195) | 0.859 | 120 | 3.39 | 0.833 | (0.646, 1.074) | 0.159 |
| 5 | 621 | 3.93 | 0.947 | (0.796, 1.126) | 0.536 | NA | | | | |
| ≥3 or ≥2^‡^ | 2,425 | 3.67 | 1.021 | (0.875, 1.190) | 0.795 | 897 | 3.39 | 1.052 | (0.882, 1.255) | 0.571 |
| Continuous | NA | | 0.967 | (0.922, 1.014) | 0.162 | NA | | 0.955 | (0.886, 1.029) | 0.227 |
| ***Individual MetS-related traits***^†^ | |  |  |  |  |  |  |  |  |  |
| No hypertension | 889 | 2.94 | Reference | | | 466 | 2.64 | Reference | | |
| Hypertension | 1,729 | 4.11 | 1.063 | (0.974, 1.159) | 0.172 | 611 | 4.02 | 1.137 | (0.996, 1.297) | 0.058 |
| No hypertriglyceridemia | 887 | 3.95 | Reference | | | 491 | 3.48 | Reference | | |
| Hypertriglyceridemia | 1,731 | 3.47 | 0.872 | (0.782, 0.973) | 0.014 | 586 | 3.13 | 0.948 | (0.791, 1.136) | 0.560 |
| No low HDL-C level | 1,381 | 3.62 | Reference | | | 769 | 3.35 | Reference | | |
| Low HDL-C level | 1,237 | 3.61 | 0.962 | (0.887, 1.043) | 0.346 | 308 | 3.10 | 0.829 | (0.720, 0.954) | 0.009 |

Notes: HR = Hazard ratio; CI = Confidence interval; MetS = Metabolic syndrome; T2D = Type 2 diabetes; HbA1c = Glycated hemoglobin; HDL = High-density lipoprotein; NA = Not available.

* The models were adjusted by age, sex, smoking status, cardiovascular disease, hyperlipidemia, chronic kidney disease, chronic obstructive pulmonary disease, liver disease, rheumatoid arthritis and other inflammatory polyarthropathies, history of falls (as a proxy indicator for frailty), osteoporosis, hyperparathyroidism, dementia, diabetic retinopathy, severe hypoglycemia, use of antidiabetic medications and systemic glucocorticoids, and baseline HbA1c level.

† MetS-related traits include albuminuria and individual MetS traits (obesity, hypertension, low HDL-cholesterol, and hypertriglyceridemia).

‡ ≥2 MetS-related traits indicate the presence of at least one additional MetS-related trait beyond T2D, while ≥3 MetS-related traits indicate at least one additional MetS-related trait beyond T2D and obesity.

## Supplementary Table 24. Incidence rates and HRs for fractures associated with albuminuria and MetS traits present at baseline in obese and non-obese patients with T2D, using the definition of MetS proposed by the International Diabetes Federation and adding albuminuria as an additional component of MetS.

| Outcome | **Obese (N =94,300)** | | | | | **Non-obese (N =40,845)** | | | | |
| --- | --- | --- | --- | --- | --- | --- | --- | --- | --- | --- |
|  | Event | Incidence rate (per 1,000 person-years) | Adjusted HR^*^ | 95% CI | P-value | Event | Incidence rate (per 1,000 person-years) | Adjusted HR^*^ | 95% CI | P-value |
| **Hip fracture** |  |  |  |  |  |  |  |  |  |  |
| ***Number of MetS-related traits***^†^ | |  |  |  |  |  |  |  |  |  |
| 1 (T2D only) | NA | | | | | 23 | 0.65 | Reference | | |
| 2 | 35 | 1.02 | Reference | | | 113 | 1.45 | 1.974 | (1.253, 3.111) | 0.003 |
| 3 | 130 | 1.05 | 0.934 | (0.640, 1.362) | 0.722 | 132 | 1.81 | 2.091 | (1.316, 3.321) | 0.002 |
| 4 | 265 | 1.46 | 1.189 | (0.826, 1.712) | 0.351 | 91 | 2.77 | 2.572 | (1.573, 4.205) | <0.001 |
| 5 | 215 | 1.71 | 1.130 | (0.776, 1.646) | 0.522 | 17 | 2.54 | 1.752 | (0.910, 3.372) | 0.093 |
| 6 | 67 | 1.88 | 1.012 | (0.658, 1.558) | 0.955 | NA | | | | |
| ≥3 or ≥2^‡^ | 677 | 1.45 | 1.075 | (0.757, 1.528) | 0.685 | 353 | 1.86 | 2.072 | (1.337, 3.211) | 0.001 |
| Continuous | NA | | 1.032 | (0.954, 1.115) | 0.433 | NA | | 1.150 | (1.032, 1.281) | 0.012 |
| ***Individual MetS-related traits***^†^ | |  |  |  |  |  |  |  |  |  |
| No albuminuria | 419 | 1.15 | Reference | | | 204 | 1.15 | Reference | | |
| Albuminuria | 293 | 2.16 | 1.331 | (1.139, 1.555) | <0.001 | 172 | 3.56 | 1.806 | (1.449, 2.251) | <0.001 |
| No hypertension | 231 | 1.07 | Reference | | | 139 | 1.13 | Reference | | |
| Hypertension | 481 | 1.69 | 0.998 | (0.843, 1.183) | 0.985 | 237 | 2.32 | 1.238 | (0.984, 1.559) | 0.069 |
| No hypertriglyceridemia | 239 | 1.63 | Reference | | | 173 | 1.87 | Reference | | |
| Hypertriglyceridemia | 473 | 1.34 | 0.824 | (0.662, 1.026) | 0.083 | 203 | 1.53 | 0.799 | (0.576, 1.108) | 0.178 |
| No low HDL-C level | 386 | 1.43 | Reference | | | 270 | 1.69 | Reference | | |
| Low HDL-C level | 326 | 1.41 | 0.972 | (0.833, 1.133) | 0.714 | 106 | 1.62 | 0.904 | (0.714, 1.144) | 0.401 |
|  |  |  |  |  |  |  |  |  |  |  |
| **Major osteoporotic fracture** |  |  |  |  |  |  |  |  |  |  |
| ***Number of MetS-related traits***^†^ | |  |  |  |  |  |  |  |  |  |
| 1 (T2D only) | NA | | | | | 80 | 2.28 | Reference | | |
| 2 | 88 | 2.59 | Reference | | | 215 | 2.77 | 1.153 | (0.886, 1.500) | 0.288 |
| 3 | 350 | 2.85 | 1.068 | (0.842, 1.354) | 0.588 | 263 | 3.63 | 1.373 | (1.050, 1.796) | 0.020 |
| 4 | 651 | 3.60 | 1.279 | (1.015, 1.612) | 0.037 | 150 | 4.59 | 1.491 | (1.105, 2.012) | 0.009 |
| 5 | 462 | 3.70 | 1.144 | (0.899, 1.455) | 0.274 | 26 | 3.90 | 0.961 | (0.601, 1.536) | 0.867 |
| 6 | 138 | 3.90 | 1.048 | (0.790, 1.389) | 0.745 | NA | | | | |
| ≥3 or ≥2^‡^ | 1,601 | 3.45 | 1.162 | (0.929, 1.452) | 0.188 | 654 | 3.46 | 1.258 | (0.983, 1.611) | 0.069 |
| Continuous | NA | | 1.010 | (0.961, 1.063) | 0.688 | NA | | 1.081 | (0.999, 1.169) | 0.052 |
| ***Individual MetS-related traits***^†^ | |  |  |  |  |  |  |  |  |  |
| No albuminuria | 1,115 | 3.07 | Reference | | | 469 | 2.66 | Reference | | |
| Albuminuria | 574 | 4.25 | 1.105 | (0.995, 1.227) | 0.062 | 265 | 5.52 | 1.431 | (1.215, 1.685) | <0.001 |
| No hypertension | 581 | 2.70 | Reference | | | 322 | 2.62 | Reference | | |
| Hypertension | 1,108 | 3.91 | 1.097 | (0.985, 1.222) | 0.093 | 412 | 4.06 | 1.134 | (0.966, 1.330) | 0.124 |
| No hypertriglyceridemia | 541 | 3.71 | Reference | | | 324 | 3.52 | Reference | | |
| Hypertriglyceridemia | 1,148 | 3.26 | 0.922 | (0.801, 1.061) | 0.258 | 410 | 3.10 | 0.964 | (0.769, 1.209) | 0.753 |
| No low HDL-C level | 929 | 3.47 | Reference | | | 526 | 3.30 | Reference | | |
| Low HDL-C level | 760 | 3.30 | 0.936 | (0.847, 1.034) | 0.193 | 208 | 3.19 | 0.879 | (0.743, 1.041) | 0.135 |

Notes: HR = Hazard ratio; CI = Confidence interval; MetS = Metabolic syndrome; T2D = Type 2 diabetes; HbA1c = Glycated hemoglobin; HDL = High-density lipoprotein; NA = Not available.

* The models were adjusted by age, sex, smoking status, cardiovascular disease, hyperlipidemia, chronic kidney disease, chronic obstructive pulmonary disease, liver disease, rheumatoid arthritis and other inflammatory polyarthropathies, history of falls (as a proxy indicator for frailty), osteoporosis, hyperparathyroidism, dementia, diabetic retinopathy, severe hypoglycemia, use of antidiabetic medications and systemic glucocorticoids, and baseline HbA1c level.

† MetS-related traits include albuminuria and individual MetS traits (obesity, hypertension, low HDL-cholesterol, and hypertriglyceridemia).

‡ ≥2 MetS-related traits indicate the presence of at least one additional MetS-related trait beyond T2D, while ≥3 MetS-related traits indicate at least one additional MetS-related trait beyond T2D and obesity.

## Supplementary Table 25. Incidence rates and HRs for fractures associated with albuminuria and MetS traits present at baseline in obese and non-obese patients with T2D, with albuminuria defined by the two most recent urine measurements within one year before the index date.

| Outcome | **Obese (N =48,727)** | | | | | **Non-obese (N =34,848)** | | | | |
| --- | --- | --- | --- | --- | --- | --- | --- | --- | --- | --- |
|  | Event | Incidence rate (per 1,000 person-years) | Adjusted HR^*^ | 95% CI | P-value | Event | Incidence rate (per 1,000 person-years) | Adjusted HR^*^ | 95% CI | P-value |
| **Hip fracture** |  |  |  |  |  |  |  |  |  |  |
| ***Number of MetS-related traits***^†^ | |  |  |  |  |  |  |  |  |  |
| 1 (T2D only) | NA | | | | | 38 | 1.07 | Reference | | |
| 2 | 11 | 0.53 | Reference | | | 157 | 2.01 | 1.063 | (0.732, 1.543) | 0.750 |
| 3 | 74 | 0.75 | 1.007 | (0.480, 2.113) | 0.985 | 179 | 3.57 | 1.506 | (1.034, 2.193) | 0.033 |
| 4 | 109 | 1.23 | 1.456 | (0.700, 3.029) | 0.315 | 46 | 3.27 | 1.286 | (0.816, 2.027) | 0.279 |
| 5 | 38 | 1.00 | 1.211 | (0.553, 2.653) | 0.632 | 6 | 2.36 | 0.796 | (0.331, 1.914) | 0.610 |
| 6 | 8 | 1.07 | 1.440 | (0.534, 3.884) | 0.471 | NA | | | | |
| ≥3 or ≥2^‡^ | 229 | 0.98 | 1.222 | (0.597, 2.504) | 0.583 | 388 | 2.68 | 1.231 | (0.863, 1.755) | 0.252 |
| Continuous | NA | | 1.126 | (0.968, 1.310) | 0.123 | NA | | 1.106 | (0.989, 1.236) | 0.078 |
| ***Individual MetS-related traits***^†^ | |  |  |  |  |  |  |  |  |  |
| No albuminuria | 130 | 0.72 | Reference | | | 238 | 1.73 | Reference | | |
| Albuminuria | 110 | 1.51 | 1.606 | (1.215, 2.123) | <0.001 | 188 | 4.38 | 1.589 | (1.287, 1.962) | <0.001 |
| No hypertension | 22 | 0.57 | Reference | | | 57 | 1.13 | Reference | | |
| Hypertension | 218 | 1.01 | 0.814 | (0.497, 1.335) | 0.415 | 369 | 2.84 | 1.148 | (0.844, 1.563) | 0.378 |
| No hypertriglyceridemia | 161 | 1.04 | Reference | | | 332 | 2.43 | Reference | | |
| Hypertriglyceridemia | 79 | 0.80 | 0.819 | (0.609, 1.102) | 0.187 | 94 | 2.16 | 0.868 | (0.679, 1.108) | 0.256 |
| No low HDL-C level | 209 | 0.95 | Reference | | | 400 | 2.41 | Reference | | |
| Low HDL-C level | 31 | 0.94 | 1.318 | (0.885, 1.965) | 0.175 | 26 | 1.82 | 0.737 | (0.489, 1.111) | 0.145 |
|  |  |  |  |  |  |  |  |  |  |  |
| **Major osteoporotic fracture** |  |  |  |  |  |  |  |  |  |  |
| ***Number of MetS-related traits***^†^ | |  |  |  |  |  |  |  |  |  |
| 1 (T2D only) | NA | | | | | 94 | 2.67 | Reference | | |
| 2 | 42 | 2.02 | Reference | | | 312 | 4.02 | 1.040 | (0.816, 1.324) | 0.753 |
| 3 | 238 | 2.41 | 0.936 | (0.661, 1.326) | 0.712 | 281 | 5.64 | 1.213 | (0.944, 1.559) | 0.131 |
| 4 | 274 | 3.12 | 1.104 | (0.780, 1.563) | 0.576 | 79 | 5.66 | 1.111 | (0.808, 1.528) | 0.517 |
| 5 | 100 | 2.66 | 0.963 | (0.656, 1.414) | 0.849 | 8 | 3.16 | 0.557 | (0.268, 1.159) | 0.117 |
| 6 | 23 | 3.09 | 1.102 | (0.647, 1.876) | 0.720 | NA | | | | |
| ≥3 or ≥2^‡^ | 635 | 2.74 | 1.009 | (0.722, 1.409) | 0.960 | 680 | 4.73 | 1.096 | (0.871, 1.379) | 0.436 |
| Continuous | NA | | 1.035 | (0.949, 1.129) | 0.437 | NA | | 1.024 | (0.943, 1.113) | 0.571 |
| ***Individual MetS-related traits***^†^ | |  |  |  |  |  |  |  |  |  |
| No albuminuria | 424 | 2.36 | Reference | | | 493 | 3.61 | Reference | | |
| Albuminuria | 253 | 3.49 | 1.256 | (1.063, 1.484) | 0.007 | 281 | 6.60 | 1.264 | (1.076, 1.486) | 0.004 |
| No hypertension | 76 | 1.97 | Reference | | | 131 | 2.62 | Reference | | |
| Hypertension | 601 | 2.81 | 0.912 | (0.703, 1.183) | 0.487 | 643 | 4.98 | 1.149 | (0.933, 1.413) | 0.190 |
| No hypertriglyceridemia | 440 | 2.85 | Reference | | | 601 | 4.43 | Reference | | |
| Hypertriglyceridemia | 237 | 2.42 | 0.873 | (0.737, 1.035) | 0.117 | 173 | 4.00 | 0.873 | (0.729, 1.046) | 0.140 |
| No low HDL-C level | 590 | 2.69 | Reference | | | 728 | 4.42 | Reference | | |
| Low HDL-C level | 87 | 2.64 | 1.128 | (0.889, 1.432) | 0.322 | 46 | 3.24 | 0.723 | (0.530, 0.987) | 0.041 |

Notes: HR = Hazard ratio; CI = Confidence interval; MetS = Metabolic syndrome; T2D = Type 2 diabetes; HbA1c = Glycated hemoglobin; HDL = High-density lipoprotein; NA = Not available.

*The models were adjusted by age, sex, smoking status, cardiovascular disease, hyperlipidemia, chronic kidney disease, chronic obstructive pulmonary disease, liver disease, rheumatoid arthritis and other inflammatory polyarthropathies, history of falls (as a proxy indicator for frailty), osteoporosis, hyperparathyroidism, dementia, diabetic retinopathy, severe hypoglycemia, use of antidiabetic medications and systemic glucocorticoids, and baseline HbA1c level.

† MetS-related traits include albuminuria and individual MetS traits (obesity, hypertension, low HDL-cholesterol, and hypertriglyceridemia).

‡ ≥2 MetS-related traits indicate the presence of at least one additional MetS-related trait beyond T2D, while ≥3 MetS-related traits indicate at least one additional MetS-related trait beyond T2D and obesity.

## Supplementary Table 26. Incidence rates and HRs for fractures associated with albuminuria and MetS traits present at baseline in obese and non-obese patients with T2D, categorised by albuminuria severity.

| Outcome | **Obese (N =96,251)** | | | | | **Non-obese (N =69,038)** | | | | |
| --- | --- | --- | --- | --- | --- | --- | --- | --- | --- | --- |
|  | Event | Incidence rate (per 1,000 person-years) | Adjusted HR^*^ | 95% CI | P-value | Event | Incidence rate (per 1,000 person-years) | Adjusted HR^*^ | 95% CI | P-value |
| **Hip fracture** |  |  |  |  |  |  |  |  |  |  |
| ***Individual MetS-related traits***^†^ | |  |  |  |  |  |  |  |  |  |
| Normoalbuminuria | 351 | 0.91 | Reference | | | 531 | 1.82 | Reference | | |
| Microalbuminuria | 201 | 1.56 | 1.250 | (1.030, 1.517) | 0.024 | 332 | 4.38 | 1.465 | (1.259, 1.704) | <0.001 |
| Macroalbuminuria | 65 | 2.88 | 1.779 | (1.296, 2.443) | <0.001 | 103 | 7.33 | 1.982 | (1.548, 2.540) | <0.001 |
| No hypertension | 43 | 0.47 | Reference | | | 121 | 1.05 | Reference | | |
| Hypertension | 574 | 1.29 | 1.242 | (0.869, 1.776) | 0.234 | 845 | 3.16 | 1.350 | (1.085, 1.679) | 0.007 |
| No hypertriglyceridemia | 391 | 1.20 | Reference | | | 717 | 2.51 | Reference | | |
| Hypertriglyceridemia | 226 | 1.07 | 0.900 | (0.746, 1.085) | 0.270 | 249 | 2.57 | 1.009 | (0.862, 1.182) | 0.911 |
| No low HDL-C level | 530 | 1.15 | Reference | | | 883 | 2.55 | Reference | | |
| Low HDL-C level | 87 | 1.12 | 1.114 | (0.864, 1.437) | 0.405 | 83 | 2.35 | 0.797 | (0.624, 1.016) | 0.067 |
|  |  |  |  |  |  |  |  |  |  |  |
| **Major osteoporotic fracture** | |  |  |  |  |  |  |  |  |  |
| ***Individual MetS-related traits***^†^ | |  |  |  |  |  |  |  |  |  |
| Normoalbuminuria | 1,021 | 2.66 | Reference | | | 1,111 | 3.83 | Reference | | |
| Microalbuminuria | 435 | 3.39 | 1.062 | (0.940, 1.201) | 0.334 | 521 | 6.94 | 1.240 | (1.105, 1.392) | <0.001 |
| Macroalbuminuria | 121 | 5.40 | 1.554 | (1.255, 1.925) | <0.001 | 142 | 10.19 | 1.532 | (1.248, 1.880) | <0.001 |
| No hypertension | 180 | 1.97 | Reference | | | 296 | 2.59 | Reference | | |
| Hypertension | 1,397 | 3.16 | 0.960 | (0.808, 1.140) | 0.642 | 1,478 | 5.57 | 1.242 | (1.077, 1.432) | 0.003 |
| No hypertriglyceridemia | 993 | 3.07 | Reference | | | 1,309 | 4.62 | Reference | | |
| Hypertriglyceridemia | 584 | 2.78 | 0.928 | (0.829, 1.039) | 0.196 | 465 | 4.84 | 1.016 | (0.905, 1.142) | 0.784 |
| No low HDL-C level | 1,367 | 2.99 | Reference | | | 1,621 | 4.71 | Reference | | |
| Low HDL-C level | 210 | 2.71 | 1.012 | (0.863, 1.188) | 0.881 | 153 | 4.37 | 0.811 | (0.676, 0.972) | 0.024 |

Notes: HR = Hazard ratio; CI = Confidence interval; MetS = Metabolic syndrome; T2D = Type 2 diabetes; HbA1c = Glycated hemoglobin; HDL = High-density lipoprotein.

* The models were adjusted by age, sex, smoking status, cardiovascular disease, hyperlipidemia, chronic kidney disease, chronic obstructive pulmonary disease, liver disease, rheumatoid arthritis and other inflammatory polyarthropathies, history of falls (as a proxy indicator for frailty), osteoporosis, hyperparathyroidism, dementia, diabetic retinopathy, severe hypoglycemia, use of antidiabetic medications and systemic glucocorticoids, and baseline HbA1c level.

† MetS-related traits include albuminuria and individual MetS traits (obesity, hypertension, low HDL-cholesterol, and hypertriglyceridemia).
